# Repression of EGFR by new biguanide 4C potentiated ovarian cancer to PARP inhibitors through down-regulation of BRCA2 and Rad51

**DOI:** 10.1038/s41419-026-08556-w

**Published:** 2026-03-18

**Authors:** Di Xiao, Jia Yao, Xin Yang, Yijun Xie, Xiaochen Zhou, Duo Li, Mei Peng, Wei Wang, Hui Zou, Xiaoping Yang

**Affiliations:** 1 Key Laboratory of Chemical Biology & Traditional Chinese Medicine Research of Ministry of Education, Key Laboratory of Study and Discovery of Small Targeted Molecules of Hunan Province, Engineering Research Center of Reproduction and Translational Medicine of Hunan Province, Key Laboratory of Protein Chemistry and Developmental Biology of Fish of Ministry of Education, Institute of Interdisciplinary Studies, Cancer Institute, School of Pharmaceutical Sciences, Health Science Center, Hunan Normal University, Changsha, Hunan China; 2 TCM and Ethnomedicine Innovation and Development International Laboratory, Innovative Material Medical Research Institute, School of Pharmacy, Hunan University of Chinese Medicine, Changsha, China

**Keywords:** Ovarian cancer, Drug development, Ubiquitylation, Homologous recombination

## Abstract

EGFR, one of the most successful therapeutic targets, has recently been found to exert a novel function for regulating homologous recombination (HR). Activation of HR is the critical event of treatment failure of PARPi in BRCA1/2 wild-type ovarian cancer (OC). Besides, the antitumor effects of biguanides have also been a focus of attention. Here, we discovered that the new biguanide **4C** inhibited HR and sensitized BRCA1/2 wild-type OC cells to PARPi by targeting EGFR. Mechanistically, EGFR promoted nuclear accumulation of both BRCA2 and Rad51, and HR activation by competitively inhibiting the binding of BRCA2 and Rad51 to E3 ubiquitin ligase c-Cbl, thereby reducing cancer cell sensitivity to PARPi following ATM-mediated DNA damage signal transmission from the nucleus to the cytoplasm. Interestingly, EGFR was downregulated by **4C**, which in turn enhanced the interaction of BRCA2 and Rad51 with c-Cbl. Consequently, BRCA2 and Rad51 were then ubiquitinated and degraded to inhibit HR and increase the sensitivity of OC to PARPi. Thus, these findings reveal that the combination of **4C** with PARPi leading to “synthetic lethality” is an effective strategy for treating BRCA1/2 wild-type OC.

## Introduction

Ovarian cancer (OC) is among the most lethal gynecological malignancies, with about 75% of patients diagnosed at stage III or IV whose 5-year survival rate is less than 10%, primarily due to the lack of effective targeted therapies [1–3]. In recent years, PARP inhibitors (PARPi) including Olaparib have made solid progress and have been granted “breakthrough therapy designation” in the clinical treatment of OC [4, 5]. However, they only cause “ synthetic lethality “ and are effective in about 15% of OC in the case of homologous recombination (HR) deficiency in the presence of BRCA1/2 mutations [6, 7]. It is well known that HR is often activated to promote resistance during PARPi treatment in BRCA1/2 wild-type cancer cells [8]. Thus, using the principle of “ synthetic lethality “ to induce HR deficiency to make BRCA1/2 wild-type patients sensitive to PARPi has great clinical application potential.

Currently, preclinical studies have evaluated several strategies to improve the activity of PARPi in BRCA1/2 wild-type cancers by inhibiting key proteins in HR, such as ATM and Rad51. For example, Riches LC et al. demonstrated that blocking HR with the ATM inhibitor AZD0156 prevented the repair of Olaparib-induced DNA damage, thereby enhancing the anticancer activity of Olaparib [9]. Similarly, Falchi F et al.‘s results showed when combined with a Rad51 inhibitor in non-mutated cells, Olaparib exhibited excellent anticancer activity comparable to Olaparib alone in BRCA-deficient cells [10]. However, despite preclinical evidence supporting the combination of PARPi with HR inhibitors, these approaches have not been approved thus far in clinic. Therefore, there is a clinical necessity to explore novel therapeutic approaches to broaden the application of PARPi in BRCA1/2 wild-type OC.

After DNA damage caused by H_2_O_2_, Ultraviolet (UV), drugs, and Interventional Radiotherapy (IR) in tumor cells, it has been observed that EGFR can transfer from the cell membrane to the nucleus and participate in DNA damage repair [11–13]. The latest finding also demonstrates that silencing EGFR can impair HR activity to block DNA damage repair [14]. However, the potential enhancement of PARPi sensitivity through the inhibition of EGFR-mediated HR remains unclear. In this study, we present that silencing EGFR effectively inhibits HR and enhances the sensitivity of PARPi, which reinforces the substantial clinical potential via targeting EGFR as a strategy to potentiate the response of OC to PARPi. However, targeting EGFR with already clinically approved small molecule kinase inhibitors is only suitable for patients with EGFR mutations, so clinical trials of these EGFR inhibitors have shown only modest activity in OC because EGFR mutations are absent in OC patients [15, 16]. Notably, previous studies in our laboratory found that proguanil, one representative of biguanides, downregulated the expression of wild-type EGFR to inhibit cancer cell proliferation [17]. Furthermore, we reported that in a series of newly synthesized derivatives of proguanil, **4C** (Supplementary Fig. 1A) exhibits superactivity in OC [18, 19]. However, whether **4C** could enhance the sensitivity of BRCA1/2 wild-type OC to PARPi by targeting EGFR to inhibit HR remains unknown. Additionally, the precise mechanism of EGFR regulating HR needs to be fully elucidated. In this study, we found that after down-regulating EGFR, **4C** enhanced the interaction between BRCA2 and Rad51 with c-Cbl. As a result, BRCA2 and Rad51 were then ubiquitinated and degraded, leading to the inhibition of HR and increased sensitivity of OC to PARPi. Thus, this combination therapy proved to be highly effective in enhancing the effectiveness and broadening the applicability of PARPi in treating BRCA1/2 wild-type OC.

## Materials and methods

### Cell culture and reagents

The HR-proficient OC cell lines SKOV3, OVCAR3, A2780, and Normal ovarian cell line IOSE80, Human umbilical cord epithelial cells (HUVEC) were obtained from iCell Bioscience Inc (Shanghai, China) and validated for authentication using the short tandem repeat (STR) method. Cells were cultured in RPMI-1640 with 10% fetal bovine serum (FBS). All cells were incubated at 37 °C in a 5% CO_2_ atmosphere. Antibodies and chemicals used in this study are listed in Supplementary Table 2.

### Human urothelial carcinoma tissues

OC tissue samples were used as previously described. Consents from all patients were obtained. Approval of this study was obtained from Hunan Normal University (IRB No: 2018027A).

### MTT assay

Cells were seeded onto 96-well plates (6.0 × 10^3^/well), then treated or not with Drugs (Olaparib, **4C**, or the combination) for 72 h at increasing concentrations. At the end of the time point, cells were incubated with MTT solution (2 mg/mL, 50 μL per well) for 4 h at 37°C. The supernatant was then discarded, the MTT was dissolved with 150 μL of DMSO, and absorbance was read at 490 nm by using a microplate reader (Biotek, SYNERGY HTX, VT, USA).

### Colony formation assay

Cells were seeded in 24-well plates at a density of 2.0 × 10^3^ cells per well. After 12 h of incubation, the cells were treated with specified drugs and allowed to grow for 6–8 days. Following the incubation period, the cells were fixed with a 10% formaldehyde solution. Subsequently, the colonies were visualized by staining with 0.1% crystal violet. The absorbance of the stained colonies was measured at 550 nm using a microplate reader (Biotek, USA). The obtained data were normalized to the control group.

### Western blot analysis

Protein extracts were separated by SDS-PAGE and transferred onto PVDF membranes. The membranes were then incubated with primary antibodies as listed in Supplementary Table 2. Following primary antibody incubation, the membranes were probed with peroxidase-conjugated anti-mouse or anti-rabbit secondary antibodies. The antigen-antibody reaction was visualized using a ChemiDoc system (Bio-Rad, Hercules, CA, USA). The band intensities on the blots were quantified using ImageJ software. The original images of all western blots were displayed in the original data file.

### Immunoprecipitation

In total, 10^7^ cells were collected and lysed using the lysis buffer obtained from Beyotime (Shanghai, China). The lysate was incubated on ice for 30 min and then centrifuged to collect the supernatant. The supernatant was subsequently blocked with protein A/G obtained from Santa Cruz (Dallas, TX) for 1 h. After another round of centrifugation, appropriate antibodies or normal IgG were added to the supernatant and incubated overnight at 4 °C. Subsequently, the protein A/G beads were added once again to capture the antibody-bound complexes, followed by centrifugation to collect the beads. The beads were then washed four times using IP buffer. Sample loading buffer (2×) was mixed with the beads and boiled for 10 min. Finally, the supernatant containing the eluted proteins was used for subsequent western blot analysis.

### RNA extraction and quantitative real-time-PCR (RT-PCR)

RNA was extracted using Trizol reagent for subsequent RT-PCR analysis. Complementary DNA (cDNA) was synthesized using a high-capacity cDNA reverse transcription kit obtained from Vazyme (Nanjing, China). RT-PCR was performed using TaqMan Gene Expression Master Mix (Vazyme, Nanjing, China) according to the manufacturer’s protocol. The primer sequences used for RT-PCR are listed in Supplementary Table 3.

### siRNA transfection

Cells were transfected with commercially available siRNA or negative control (NC) siRNA obtained from Ribobio (Shanghai, China) using Lipofectamine 6000 transfection reagent (Invitrogen, Eugene, USA). Briefly, cells were seeded in six-well plates at a density of 1.0 × 10^5^ cells per well. After 12 h, the cells were transfected with 50 nM RNAi oligonucleotides and 50 nM Negative Control siRNA using Lipofectamine 6000 in the absence of FBS for 5 h. Following a washing step with PBS, the medium was replaced with RPMI-1640 medium and incubated for an additional 24 h. Cell protein was collected, and the specific silencing effect was confirmed by Western blot analysis. The sequences of the siRNA used are provided in Supplementary Table 4.

### Knockdown or overexpression of EGFR by transfection with lentiviral vectors

Lentiviral vectors for EGFR knockdown (shCtrl, shEGFR-1, shEGFR-2, Supplementary Table 5) and EGFR overexpression (overexpression Ctrl (OC), overexpression EGFR (OE) were constructed by GeneChem Co., Ltd. (Shanghai, China). Briefly, cells were seeded in six-well plates at a density of 2.0 × 10^5^ cells per well. After 12 h, the lentiviruses were added to the wells with 1 mL of 5 A medium and 40 μl of transfection reagent (GeneChem, Shanghai, China) to incubate the cells for 12 h. Following a washing step with PBS, the medium was replaced with 5A medium and incubated for an additional 72 h. Finally, puromycin was added to the cell culture flask to eliminate untransfected cells.

### Immunofluorescence

Cells were plated on glass coverslips, washed three times with PBS, and fixed in a 4% paraformaldehyde solution. After another wash with PBS, cells were permeabilized using 0.2% Triton X-100. Following another wash with PBS, cells were incubated with 4% BSA for 30 min. The primary antibody was then added, and cells were incubated overnight at 4 °C. After three washes with PBS, a secondary antibody labeled with DyLight 549 (Proteintech, Chicago, USA) or Alexfluor 488 (Proteintech, USA) was added to the glass coverslips and incubated for 1 h. Nuclei were stained with DAPI and then fixed with glycerine. Finally, the samples were visualized and photographed under a fluorescence microscope. Immunofluorescence staining analysis was performed using ImageJ software, and the data were normalized to the vehicle control.

### HR assay

The cells (3 × 10^5^/well) were transfected with the HR reporter plasmid pDRGFP-expressing or siRNA in six-well plastic culture plates. The next day, cells were transfected with the Sce-I-expressing plasmid (pCBASce-I; AddGene) using Lipofectamine 6000 according to the manufacturer’s instructions. Immediately after transfection, cells were treated with 4 C or PARPi. Subsequently, cells were collected and subjected to flow cytometry analysis. The relative HR capacity was determined by calculating the ratio of the percentage of GFP-positive cells in Sce-I-transfected cells to the basal percentage of GFP signal in the mock control.

### Proteomic analysis

SKOV3 cells were inoculated in a 75-cm^2^ culture bottle and treated with **4C** for 24 h. The cells were then washed twice with PBS and collected. The label-free quantitative proteomics and bioinformatics analyses were performed by Bioprofile Technology Co., Ltd. (China, Shanghai). Each group was tested independently three times. R software was used to analyze and plot the data. When the selected protein accumulates or decreases, log2(FC)> = 1 and *P* < 0.05 were chosen as the criteria. The GO term enrichment analysis was performed via the “cluster profiler” and “org.Hs.eg.db” R packages.

### Mass spectrum analysis

The peptide samples were analyzed using Thermo Fisher Exactive^TM^ plus Obitrap mass spectrometry. Mass spectrometry analysis was performed at Bioprofile Technology Co., Ltd. (China, Shanghai) in the positive-ion mode, employing data-dependent acquisition (DDA).

### Animals

Female BALB/c nude mice aged 4–6 weeks (*n* = 92) were procured from Hunan SJA Laboratory Animal Co., Ltd (Changsha, China). The experimental protocol was approved by the Ethics Committee of Hunan Normal University (D2023029). The mice were randomly divided into different groups. Subcutaneous xenograft models were established by injecting shCtrl SKOV3 or ShEGFR- SKOV3 cell suspensions into the mice. Random assignment of animals was performed for each treatment group. Tumor volumes were calculated using the formula: 1/2 × long diameter × short diameter^2^. Once the tumor reached a size of 70–100 mm^3^, the mice were treated with vehicle control, **4C** (2% DMSO + 98% PBS) via intraperitoneal injection, PARPi (0.5% CMC-Na) via gavage and a combination of 4 C and Olaparib five times a week. Tumor volumes and mouse weights were measured every two days. After 21 days of treatment, the mice were euthanized. The liver and kidney tissues were embedded in paraffin, sectioned, and subjected to histological analysis using H&E staining. Additionally, all tumors were fixed in formalin for immunohistochemistry. A tumor metastasis model was established by injecting SKOV3 cells expressing luciferase via the tail vein. Five days later, mice were anesthetized and intraperitoneally injected with 150 mg/kg D-luciferin potassium for in vivo bioluminescence imaging to monitor metastasis formation and subsequently assign the animals to experimental groups. The mice received daily treatments as follows: vehicle control, 4C (2% DMSO + 98% PBS) via intraperitoneal injection, Olaparib (0.5% CMC-Na) via oral gavage, and a combination of 4C and Olaparib. After 14 days of treatment, the mice were again injected intraperitoneally with 150 mg/kg D-luciferin potassium for in vivo imaging to evaluate the effects of the 4C and Olaparib combination on metastatic lesions. The investigators assessing the results were blinded to the group allocation.

### Histology

Tissues were fixed in a 4% paraformaldehyde solution for 24 h, followed by embedding in paraffin and sectioning into 7 μm slices. The sections were then stained with haematoxylin and eosin (H&E) and evaluated for disease grading. For immunohistochemistry staining, the sections were deparaffinized and rehydrated using xylene, followed by a series of ethanol washes (100%, 95%, and 75%). Subsequently, the sections were treated with 3% H_2_O_2_ for 20 min to block endogenous peroxidase activity, washed with PBS, and subjected to heat-induced antigen retrieval in Tris-EDTA solution for 5 min using a pressure cooker. The sections were then incubated overnight at 4 °C with the corresponding primary antibodies. After washing with PBS, the sections were incubated with Reagent 2 and Reagent 3 (Goat hypersensitivity two-step detection kit, ZSGB-BIO, Beijing, China) according to the manufacturer’s instructions. The sections were stained using the DAB Substrate Kit (Cell Signaling, Beverly, MA, USA) and counterstained with Gill’s haematoxylin (Solarbio, Beijing, China). Finally, the sections were dehydrated, mounted using neutral resins (Solarbio, China), and subjected to immunohistochemistry staining analysis using ImageJ software. The data were normalized to the vehicle control.

### Statistical analyses

The data of three independent experiments were expressed as mean ± SD. Statistical analysis was performed with SPSS 20.0. Normality was assessed by the Shapiro–Wilk test, and homogeneity of variances was confirmed with Levene’s test prior to parametric analyses. Comparisons between two groups were analyzed by a two-tailed Student’s *t* test with Bonferroni correction for multiple comparisons where applicable. All experiments were repeated at least three times. *P* < 0.05 was thought as statistically significant.

## Results

### EGFR is a critical mediator of PARPi resistance in BRCA1/2 wild-type OC cells

**Fig. 1:**
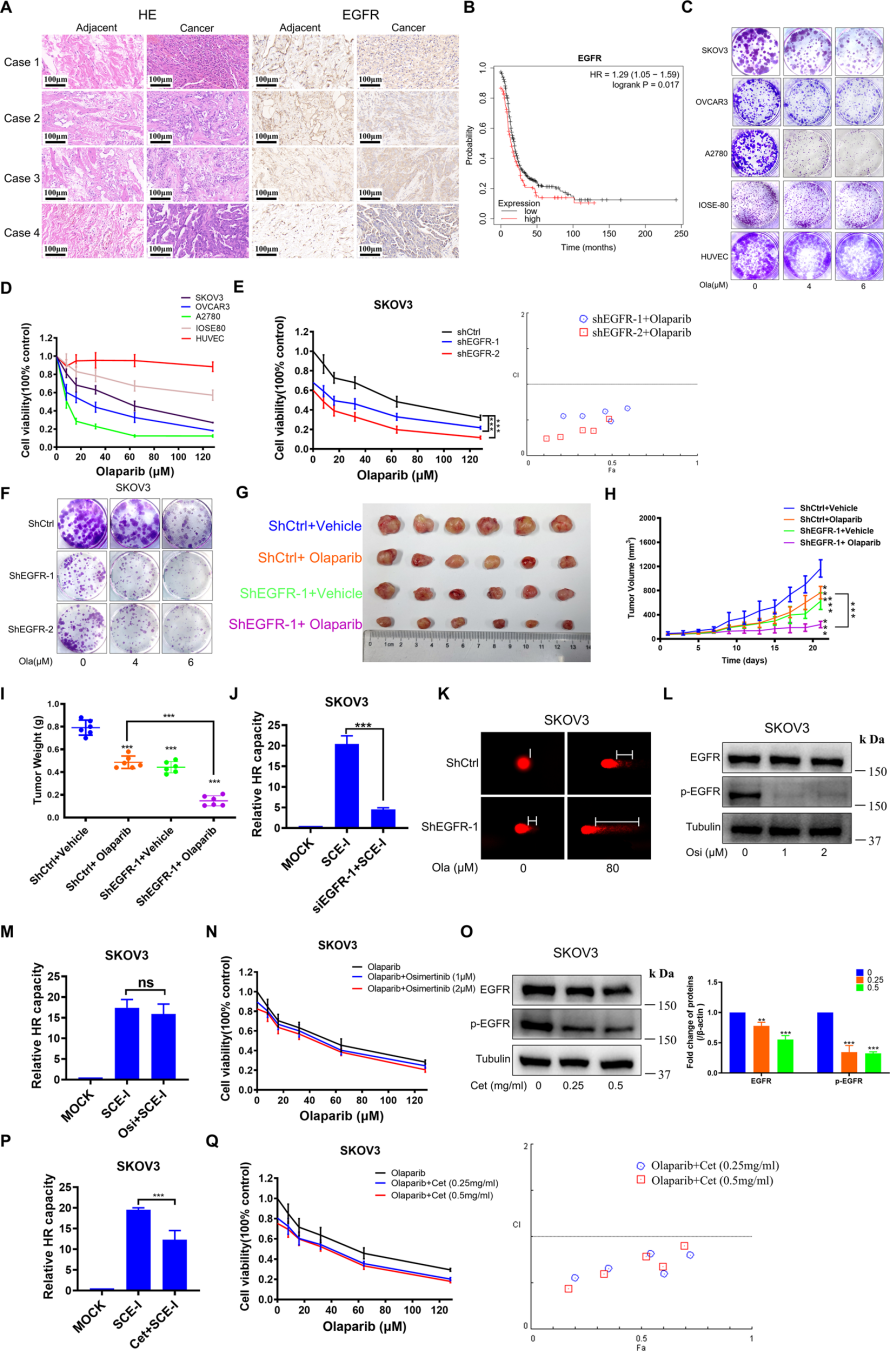
The effect of EGFR expression on the sensitivity of OC cells to Olaparib. **A** Representative images showing HE and IHC. **B** Kaplan–Meier plot illustrating overall survival of OC patients. **C**, **D** The cell viability after Olaparib treatment was evaluated by colony formation or MTT assay. **E**, **F** The cell viability of SKOV3 after Olaparib treatment was evaluated by MTT (**E**) and colony formation (**F**) assay. **G**–**I** Olaparib was administered intragastrically at a dose of 100 mg/kg five times a week. Tumors volumes were measured every 2 days (**H**), and after 21 days tumors were harvested for photography (**G**) and weight measurements (**I**). Results are presented as mean ± SD (6 mice/group). ***P < 0.001. **J** HR-specific repair of DNA damage in SKOV3 was analyzed using the DR-GFP chromosomal reporter system and detected by a flow cytometer. **K** The DNA damage of SKOV3 treated with Olaparib for 24 h was measured by the comet assay. **L**, **O** SKOV3 subjected to Osimertinib (**L**) or Cetuximab (**O**) for 24 h, and the changes of the indicated proteins were analyzed by WB. **M**, **P** SKOV3 cells subjected to Osimertinib (2 μM) or Cetuximab (0.5 mg/kg) for 24 h, and HR-specific repair of DSBs was analyzed using the flow cytometer. **N** The cell viability of SKOV3 treated with Osimertinib and Olaparib was evaluated by MTT assay. **Q** The cell viability of SKOV3 treated with Cetuximab and Olaparib was evaluated by MTT assay. Ola Olaparib, Osi Osimertinib, Cet Cetuximab (*n* = 3, ns, no significant difference, **P < 0.01, ***P < 0.001).

In order to assess the clinical relevance of EGFR in OC, we explored EGFR expression in OC tissues compared to normal tissues by analyzing clinically-sourced OC tissues and publicly available datasets (https://ualcan.path.uab.edu/analysis.html). The findings showed that EGFR was highly expressed in 75% of patients **(**Fig. 1A, Supplementary Fig. 1B, and Table S1), consistent with the high expression of EGFR in OC observed in available datasets (Supplementary Fig. 1C) and Wen et al’s observations [15]. Moreover, the Kaplan–Meier (http://kmplot.com/analysis/) survival analysis revealed that EGFR expression was negatively correlated with overall survival of OC patients (Fig. 1B). In addition, our results showed that EGFR was highly expressed in OC cell lines SKOV3 and OVCAR3 compared with normal ovarian epithelial cells (Supplementary Fig. 1D, E). Notably, knockdown of EGFR significantly reduced the proliferation of OC cells, while overexpression of EGFR promoted cell growth (Supplementary Fig. 1F, G). Those results indicated that EGFR may represent a potential therapeutic target for OC.

Russo et al. recently demonstrated that silencing EGFR can block HR [14]. However, it remains unclear whether impeding HR by downregulation EGFR can enhance the sensitivity of PARPi, including Olaparib. Therefore, we first analyzed the sensitivity of BRCA1/2 wild-type OC cell lines with different EGFR expressions to Olaparib. Strikingly, cells with intrinsically low EGFR expression (A2780) or those (SKOV3, OVCAR3) subjected to EGFR knockdown exhibited significantly increased sensitivity to Olaparib (Fig. 1C–F and Supplementary Fig. 1H), suggesting a strong correlation between EGFR expression and PARPi sensitivity in OC cells. These conclusions are consistent with a previous publication, which observed that EGFR wild-type tumors are insensitive to PARPi [20]. To further validate this conclusion, we overexpressed EGFR in the low-EGFR A2780 cells and found that EGFR overexpression led to enhanced resistance to olaparib, with the IC_50_ increasing from 9.2 to 16.3 μM (Supplementary Fig. 1I, J). Next, we also found a significant suppression of tumor growth in shEGFR groups compared to shCtrl groups upon treatment with Olaparib (Fig. 1G–I). These findings suggested that the expression of EGFR is a critical factor affecting Olaparib sensitivity both in vitro and in vivo. To further explore the underlying mechanisms, we evaluated HR capability and DNA damage when knocking down EGFR. The findings suggest that the efficiency of HR was dramatically reduced while DNA damage was largely increased after Olaparib treatment in the knocking down EGFR group (Fig. 1J, K and Supplementary Fig. 1K, L). These results demonstrate the therapeutic potential of enhancing Olaparib efficacy in BRCA1/2 wild-type OC through EGFR inhibition. However, we observed that cetuximab, but not Osimertinib, effectively impaired HR efficiency and sensitized cells to Olaparib **(**Fig. 1L–Q and Supplementary Fig. 1M, N). Given that Cetuximab downregulates total EGFR, whereas Osimertinib merely inhibits kinase activity without altering EGFR expression (Fig. 1L, O), we propose that suppression of HR activation requires depletion of total EGFR rather than sole inhibition of its tyrosine kinase function.

### New biguanide 4C bound to EGFR and promoted its ubiquitination and degradation to inhibit OC cell proliferation

**Fig. 2:**
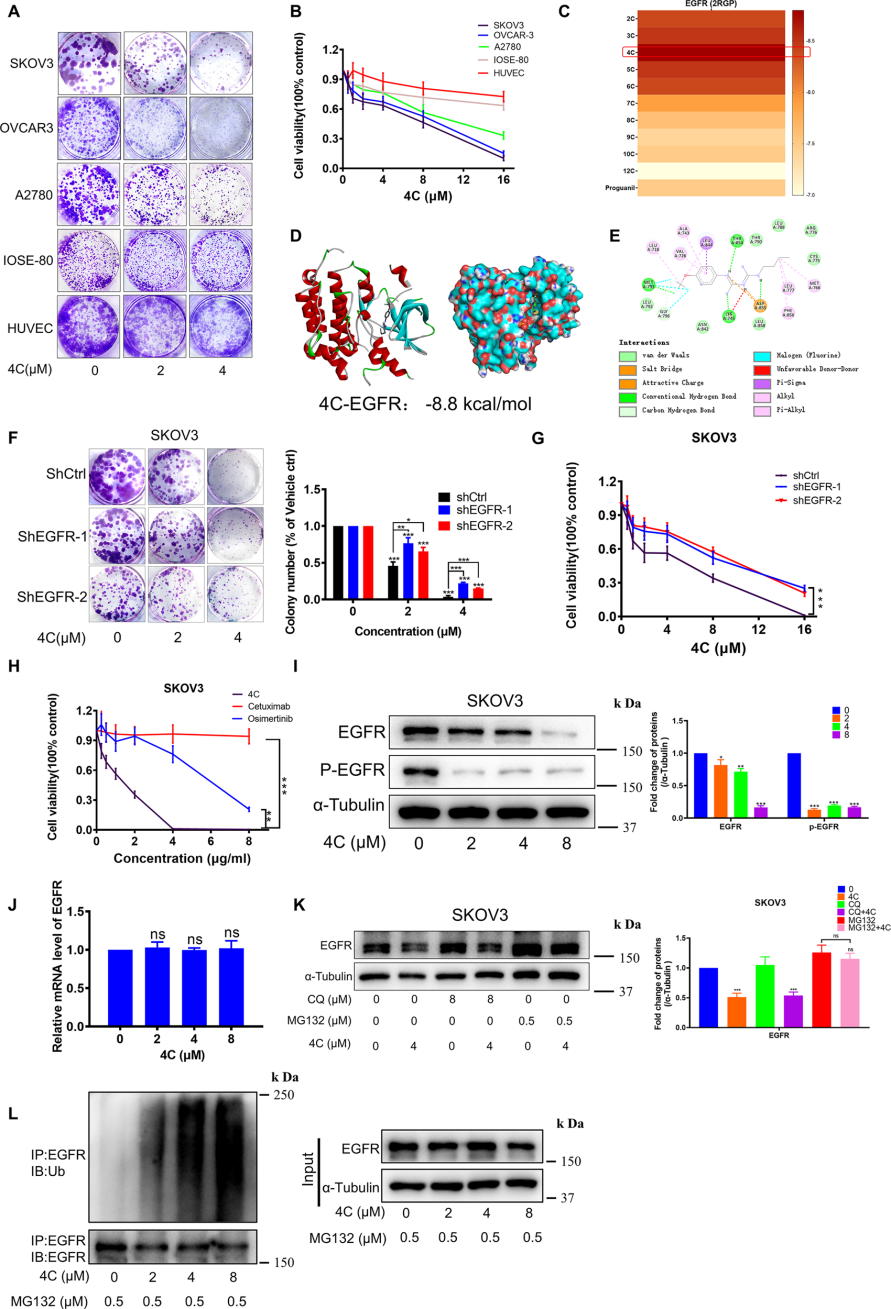
4C inhibited OC cell proliferation by targeting EGFR. **A**, **B** The cell viability after **4C** treatment was evaluated by MTT or colony formation assay. **C** The binding energy of different biguanides to EGFR (PDB ID − 2RGP) was calculated by Vina docking. **D**, **E** The 3D (**D**) and the 2D (**E**) interaction figures of ligand (**4C**) with EGFR (PDB ID − 2RGP). **F**, **G** The cell viability after **4C** treatment were evaluated by colony formation (**F**) or MTT (**G**) assay. **H** The cell viability after indicated drugs treatment were evaluated by MTT assay. **I**, **J** SKOV3 were starved for 6 h and subjected to **4C** for 24 h, and the changes of the indicated proteins or mRNA were analyzed by WB or RT-PCR. **K** SKOV3 were starved for 6 h and then pretreated with chloroquine or MG132 for 12 h, and cells were then treated with **4C** for 12 h. The changes in EGFR were detected by WB. **L** SKOV3 were starved for 6 h and then pretreated with MG132 for 12 h, and cells were then treated with **4C** for 12 h and lysed for immunoprecipitation using anti-EGFR antibody, followed by WB with the indicated antibodies. (*n* = 3, ns, no significant difference, *P < 0.05, **P < 0.01, ***P < 0.001).

Previous studies in our laboratory found that proguanil inhibits cancer cell proliferation by reducing total EGFR [17]. To further improve the antiproliferation activity of proguanil, we synthesized a series of derivatives and found that **4C** had the best biological activity in OC, whereas the normal cells, IOSE80, and HUVEC exhibited resistance to **4C (**Fig. 2A, B), indicating that **4C** is safe while killing OC cells. Vina molecular docking results showed that **4C** had the highest binding affinity with EGFR (−8.8 kcal/mol) among all compounds, and formed a hydrogen bond with Lys745, Met 793, Thr854 amino acids of EGFR (Fig. 2C–E). More importantly, we found that in SKOV3 and OVAR3 cells with high levels of EGFR expression, the anti-proliferative effect of **4C** was significantly weakened after shEGFR (Fig. 2F, G and Supplementary Fig. 2A). Conversely, overexpression of EGFR significantly enhanced sensitivity to **4C** (Supplementary Fig. 2B, C). These results indicated that EGFR is the target of **4C**. Previous studies have shown that Cetuximab and Osimertinib suppress p-EGFR in OC cells. Consequently, we proceeded to compare the antitumor activity of **4C** with these EGFR inhibitors. Interestingly, **4C** exhibited superior antitumor activity compared to the EGFR inhibitors Cetuximab and Osimertinib (Fig. 2H and Supplementary Fig. 2D). Additionally, we found that **4C** not only significantly inhibited p-EGFR but also downregulated the expression of total EGFR (Fig. 2I and Supplementary Fig. 2E). As commonly understood, inhibiting protein synthesis or enhancing degradation are the two main methods to decrease protein expression. We first examined the impact of **4C** on the expression of EGFR mRNA and found that **4C** had no effect on it (Fig. 2J and Supplementary Fig. 2F), suggesting that **4C** does not inhibit EGFR expression at the transcriptional level but rather through post-translational modification. Protein degradation after translation mainly occurs through two pathways: the ubiquitin-proteasome pathway and the lysosomal proteolysis-mediated pathway. Our investigation revealed that MG132, a proteasome inhibitor, was able to reverse EGFR downregulation caused by **4C**, while the lysosomal inhibitor did not show the same effect (Fig. 2K and Supplementary Fig. 2G). When EGFR degradation was blocked by MG132, we found that **4C** increased ubiquitination of EGFR (Fig. 2L and Supplementary Fig. 2H). All these results indicated that **4C** bound to EGFR and promoted its ubiquitination and degradation in the proteasome to inhibit OC cell proliferation. We further evaluated the specificity of **4C** targeting EGFR. Molecular docking analysis demonstrated that **4C** exhibited the highest binding affinity with EGFR compared to other tyrosine kinase receptors (Supplementary Fig. 2I). To investigate whether **4C** has potential off-target effects, we analyzed its impact on the expression of representative receptors IGF-1R and HER2. Our data confirmed that **4C** had no effect on the expression of either IGF-1R or HER2 (Supplementary Fig. 2J). To further validate EGFR-targeting specificity across genetic contexts, we treated H1975 cells which harbor EGFR L858R/T790M mutations with **4C**. Significantly, **4C** similarly downregulated mutant EGFR expression (Supplementary Fig. 2K), demonstrating that EGFR degradation by **4C** occurs independently of mutation status. This mutation-agnostic activity suggests potential therapeutic utility of **4C** also in EGFR-mutant cancers, including treatment-resistant NSCLC.

### 4C inhibited HR-mediated repair of DNA damage by down-regulating EGFR

**Fig. 3:**
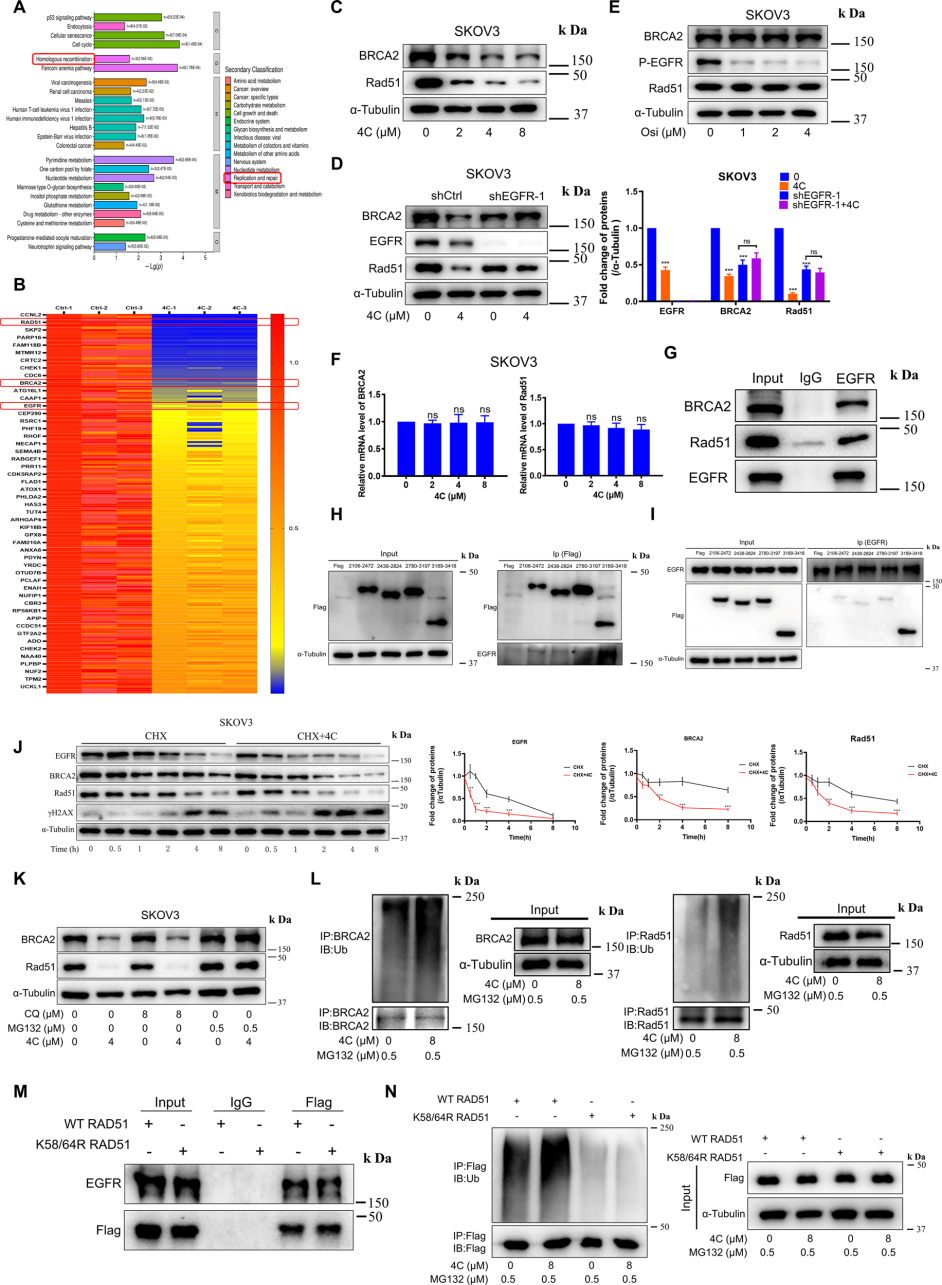
4C promoted the ubiquitination and degradation of BRCA2 and Rad51. **A**, **B** KEGG pathway enrichment or Heatmap analysis of proteins downregulated after **4C** treatment for 24 h in SKOV3 cells. **C**–**F** Cells were starved for 6 h and subjected to **4C** or Osimertinib for 24 h, and the changes of the indicated proteins or mRNA were analyzed by WB and RT-PCR. **G** SKOV3 were lysed to immunoprecipitation using anti-EGFR antibody, followed by WB with the indicated antibodies. **H**, **I** SKOV3 transfected with Flag-BRCA2 truncated and then lysed for immunoprecipitation using anti-Flag (**H**) or anti-EGFR (**I**) antibody, followed by WB with indicated antibodies. **J** SKOV3 cells were treated with CHX (20 µg ml^−1^) or CHX (20 µg ml^−1^) + **4C** (8 µM) for the time indicated. The changes of indicated proteins were detected by WB. **K**, **L** SKOV3 cells were starved for 6 h and then pretreated with chloroquine or MG132 for 12 h, and cells were then treated with **4C** for 12 h. The changes of indicated proteins were detected by WB. **M** CO-IP analysis of indicated protein interaction in SKOV3. **N** CO-IP analysis of the ubiquitination of Rad51 in SKOV3. (*n* = 3, ns, no significant difference, *P < 0.05, **P < 0.01, ***P < 0.001).

**Fig. 4:**
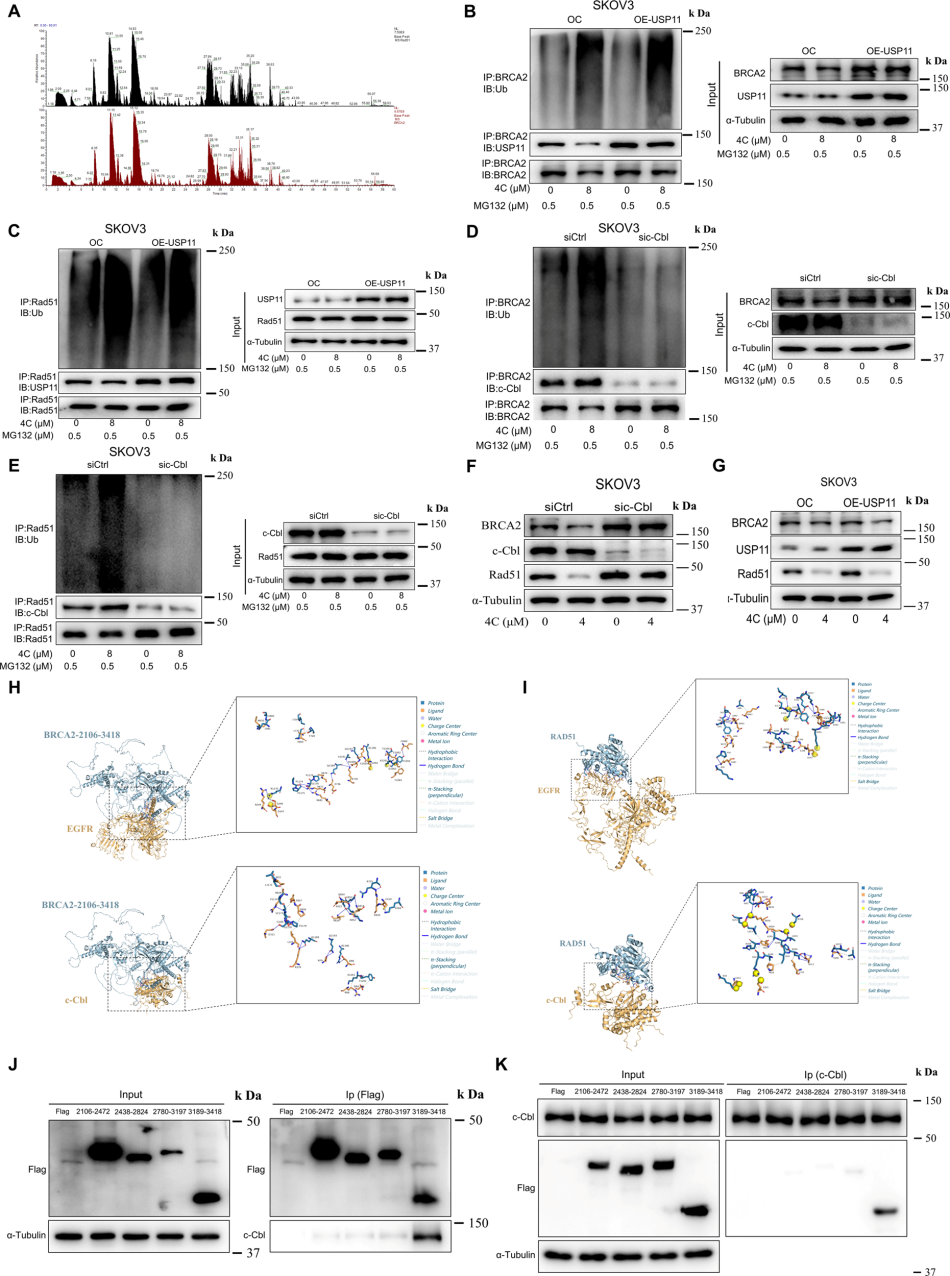
EGFR stabilized BRCA2 and Rad51 via competitive inhibition of Cbl-mediated ubiquitination. **A** The interacting proteins of BRCA2 or Rad51 in SKOV3 were identified by IP-MS. **B**–**E** SKOV3 were transfected with USP11 plasmid or sic-Cbl. After 48 h, cells were starved for 6 h and treated with **4C** for 12 h and then lysed to immunoprecipitation using anti-BRCA2 or anti-Rad51, followed by WB with Ub. **F**, **G** SKOV3 transfected with USP11 plasmid or siCbl and subjected to the indicated dose of **4C** for 24 h, the changes of the indicated proteins were tested by WB. **H** The combination of BRCA2 with EGFR or c-Cbl is predicted by HDOCK. In brief, BRCA2-2106-3418 (The three-dimensional structure of BRCA2 (2106-3418) was predicted using AlphaFold2), c-Cbl (UniProt ID: P22681), EGFR (UniProt ID: P00533) serve as protein structure files. Appropriate docking parameters were set, and HDOCK was used for protein-protein docking. The results include two indicators: Docking Score and confidence score Confidence_score = 1.0/[1.0 + e0.02*(Docking_Score+150)]. **I** The combination of Rad51 with EGFR or c-Cbl is predicted by HDOCK. Rad51 (UniProt ID: Q06609) serve as protein structure files. **J**, **K** SKOV3 transfected with Flag-BRCA2 truncated and then lysed to immunoprecipitation using anti-Flag (**J**) or anti-c-Cbl (**K**) antibody, followed by WB with indicated antibodies.

To investigate the downstream signaling pathway after downregulation of EGFR by **4C**, the proteomic analysis was employed. The results revealed that HR signaling pathway was enriched (Fig. 3A and Supplementary Fig. 3A). The heatmap analysis and WB results further demonstrated that BRCA2 and Rad51, which are closely related to HR [21], were significantly downregulated after **4C** treatment (Fig. 3B, C and Supplementary Fig. 3B). Interestingly, BRCA2 and Rad51 were also reduced upon knockdown of EGFR (Supplementary Fig. 3C, D), which is consistent with **4C** treatment. Furthermore, we found that the downregulation of BRCA2 and Rad51 induced by **4C** was significantly diminished when EGFR was silenced (Fig. 3D and Supplementary Fig. 3E), implying that **4C** reduced the expression of BRCA2 and Rad51 by targeting EGFR. However, the EGFR inhibitor Osimertinib had no effect on BRCA2 and Rad51 (Fig. 3E and Supplementary Fig. 3F), further highlighting that the mechanism of **4C**-downregulated EGFR is distinct from EGFR TKIs. Although cetuximab also downregulated the expression of BRCA2 and Rad51 at the IC_50_ concentration, its effect is significantly weaker than that of **4C** (Supplementary Fig. 3G). Subsequently, the specific mechanism of how **4C** regulates BRCA2 and Rad51 via EGFR was explored. WB and PCR results indicated that the protein expression of BRCA2 and Rad51 was downregulated by **4C** treatment or EGFR knockdown, but there was no effect on their transcriptional levels (Fig. 3C, F and Supplementary Fig. 3B–D, H), suggesting that BRCA2 and Rad51 are regulated by EGFR via post-translational modification. Thus, we speculated that the stability of BRCA2 and Rad51 was promoted by binding to EGFR. Consistent with our reasoning, there was an interaction between EGFR and BRCA2 or Rad51 (Fig. 3G and Supplementary Fig. 3I). Furthermore, Flag-tagged truncations of BRCA2 were designed to investigate the interaction domains of BRCA2 bound to EGFR. The results demonstrated that EGFR interacts with the 3189–3418 truncation of BRCA2 (Fig. 3H, I and Supplementary Fig. 3J, K). On the other hand, **4C** significantly shortened the half-life of BRCA2 and Rad51 when novel protein synthesis was blocked by cycloheximide (CHX) (Fig. 3J and Supplementary Fig. 3L). Interestingly, **4C**-induced downregulation of BRCA2 and Rad51 was rescued by MG132 but not chloroquine (Fig. 3K and Supplementary Fig. 3M), further indicating that **4C** promoted BRCA2 and Rad51 degradation in a proteasome-dependent manner. Moreover, the ubiquitination of BRCA2 and Rad51 were detected after **4C** treatment in the presence of MG132 for blocking the protein degradation. As shown in Figs. 3L and 4C dramatically increased ubiquitination of BRCA2 and Rad51. Studies have shown that K58 and K64 of Rad51 are ubiquitylated sites in human cells [22, 23]. Our results showed that although K58/64R Rad51 had similar interaction ability with EGFR as WT Rad51, **4C** could not enhance the ubiquitination of K58/64 R Rad51 compared with WT Rad51 (Fig. 3M, N and Supplementary Fig. 3N, O), confirming that K58 and K64 are the major modification sites of Rad51 promoted by **4C**. Collectively, the data indicate that **4C** induced BRCA2 and Rad51 ubiquitination and degradation in the proteasome by targeting EGFR.

### 4C promoted BRCA2 and Rad51 binding to c-Cbl, and then ubiquitination and degradation

As it is well known that EGFR does not directly participate in the ubiquitination or deubiquitination of substrates, the proteins directly involved in the ubiquitination of BRCA2 and Rad51 were investigated via IP-MS. The results show that there was strong binding among BRCA2, Rad51, c-Cbl, and USP11, suggesting that either E3 ubiquitin ligase c-Cbl or the deubiquitinase USP11 may be directly involved in ubiquitination of BRCA2 and Rad51 after **4C** treatment (Fig. 4A). Interestingly, we observed that silencing c-Cbl rather than overexpression of USP11 significantly blocked **4C**-induced ubiquitination of BRCA2 and Rad51 (Fig. 4B–E and Supplementary Fig. 4A, B). Consistently, the expression of BRCA2 and Rad51 increased after silencing c-Cbl (Supplementary Fig. 4C). Studies have shown that the binding of c-Cbl to the substrate is a key step to promote protein ubiquitination, so we further examined the influence of **4C** on the binding of c-Cbl with BRCA2 or Rad51 and discovered that **4C** enhanced their interaction (Fig. 4B–E and Supplementary Fig. 4A, B). Interestingly, we found that the degradation of BRCA2 and Rad51induced by **4C** also rescued by silencing c-Cbl rather than overexpressing USP11 (Fig. 4F, G and Supplementary Fig. 4D, E). These findings suggested that c-Cbl but not USP11 participated in the event of the ubiquitination and degradation of BRCA2 and Rad51 after **4C** treatment. Since BRCA2 and Rad51 are stabilized by EGFR, as described previously, we further explored whether stabilization of EGFR on BRCA2 and Rad51 is associated with c-Cbl. Protein-protein docking results revealed that there is a competitive binding between EGFR and c-Cbl to BRCA2, Rad51 (Fig. 4H, I). In addition, both c-Cbl and EGFR have a binding to the same truncations of BRCA2 (3189-3418) (Fig. 4J, K and Supplementary Fig. 4F, G). These results indicated that EGFR stabilized BRCA2 and Rad51 by competitively inhibiting c-Cbl interaction with BRCA2 and Rad51, and **4C** promoted the interaction of BRCA2 and Rad51 with c-Cbl to ubiquitination and then degradation.

### 4C increased the sensitivity of PARPi in BRCA1/2 wild-type OC cells by blocking PARPi-induced nucleus transfer of BRCA2 and Rad51

**Fig. 5:**
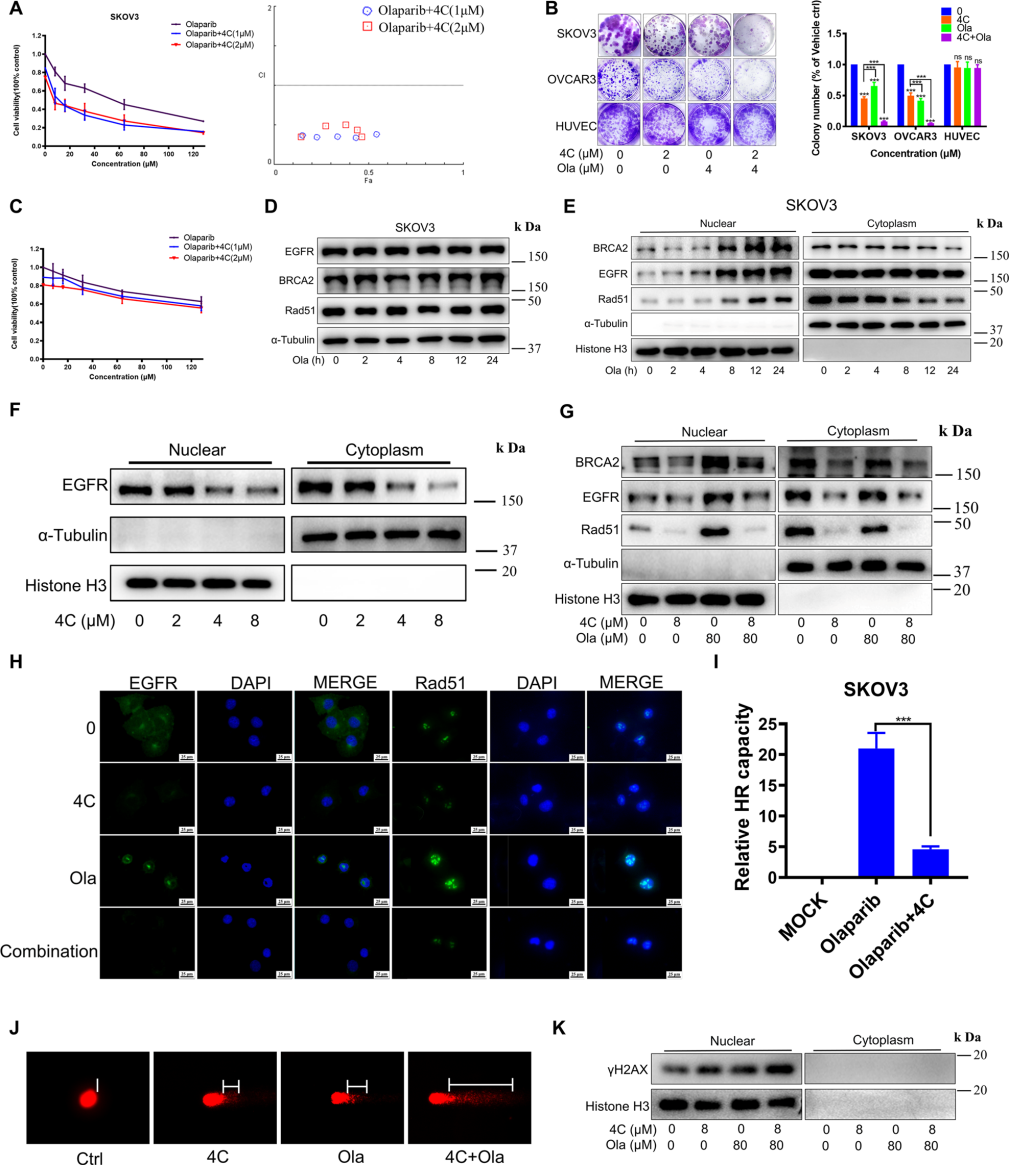
The effect and mechanism of 4 C combined with PARPi leads to “synthetic lethality” in BRCA wild-type OC. **A**–**C** The cell viability after **4C** and Olaparib treatment were evaluated by MTT and Colony formation assay. The combination index (CI) was calculated using CompuSyn software. **D** WB analysis of indicated proteins after Olaparib (80 µM) treatment for indicated times. **E**–**G** SKOV3 were treated with Olaparib or **4C** and separated into nuclear and cytoplasmic fractions. These fractions were then detected by WB. **H** SKOV3 were subjected to Olaparib (80 μM) and **4C** (8 μM) for 24 h, and the changes of indicated proteins were detected by immunofluorescence. **I** SKOV3 were subjected to Olaparib (80 μM) and **4C** (8 μM) for 24 h, and HR was analyzed using flow cytometer. **J** SKOV3 were subjected to Olaparib (80 μM) and **4C** (8 μM) for 24 h, and the degree of DNA damage was measured by comet assay. **K** SKOV3 were subjected to **4C** and Olaparib and cell lysates were separated into nuclear and cytoplasmic fractions. These fractions were then detected by WB. Ola, Olaparib. (*n* = 3, ns, no significant difference, ***P < 0.001).

Since **4C** significantly inhibited HR in BRCA1/2 wild-type OC cells, we would like to determine whether **4C** sensitizes OC cells to PARPi. As expected, our research demonstrated a synergistic effect of **4C** and PARPi with all combination index (CI) are below 0.6 (Fig. 5A, B and Supplementary Fig. 5A). Further, Talazoparib and Niraparib, two other PARPi, exhibited similar synergy patterns with **4C**, implying the mechanistic relationship between **4C** and PARP inhibition (Supplementary Fig. 5B–D). In contrast, we did not observe any combination synergy in non-tumorigenic cells IOSE-80 with low EGFR expression (Fig. 5C), indicating the importance of EGFR expression on combination effects. Building on our previous finding that EGFR downregulation enhanced Olaparib sensitivity by promoting ubiquitination-mediated degradation of BRCA2 and RAD51 to suppress HR, we further assessed whether Olaparib treatment upregulates EGFR, BRCA2, and RAD51 expression to activate HR and confer resistance. Unexpectedly, Olaparib had no effect on these total proteins (Fig. 5D and Supplementary Fig. 5E). Recognizing that DNA damage responses occur primarily in the nucleus, we detected whether Olaparib differentially affected the subcellular distribution of these proteins. As expected, nuclear-cytoplasmic fractionation assays revealed that EGFR, BRCA2, and Rad51 increased in the nucleus but decreased in the cytoplasm after Olaparib treatment (Fig. 5E and Supplementary Fig. 5F), indicating that Olaparib promoted the nuclear translocation of EGFR, BRCA2, and Rad51. In contrast, **4C** or knockdown EGFR inhibited nuclear transfer of EGFR, BRCA2, and Rad51, thereby blocking PARPi-induced activation of HR (Fig. 5F–I and Supplementary Fig. 5G–I). Next, the Comet assay was used to directly assess whether PARPi-induced DNA damage was enhanced in the presence of **4C**. Results showed that this combination significantly increased comet tail length, further confirming the increase of DNA damage in the combination setting (Fig. 5J and Supplementary Fig. 5J). Correspondingly, the expression of γH2AX was further increased when subjected to combination treatment with **4C** and PARPi (Fig. 5K and Supplementary Fig. 5K). These results implied that **4C** significantly increased PARPi-induced DNA damage by inhibiting nuclear transfer of BRCA2 and Rad51 and the activation of HR, leading to “synthetic lethality” in BRCA1/2 wild-type OC.

### 4C had no effect on Olaparib-induced export of ATM from the nucleus to the cytoplasm

**Fig. 6:**
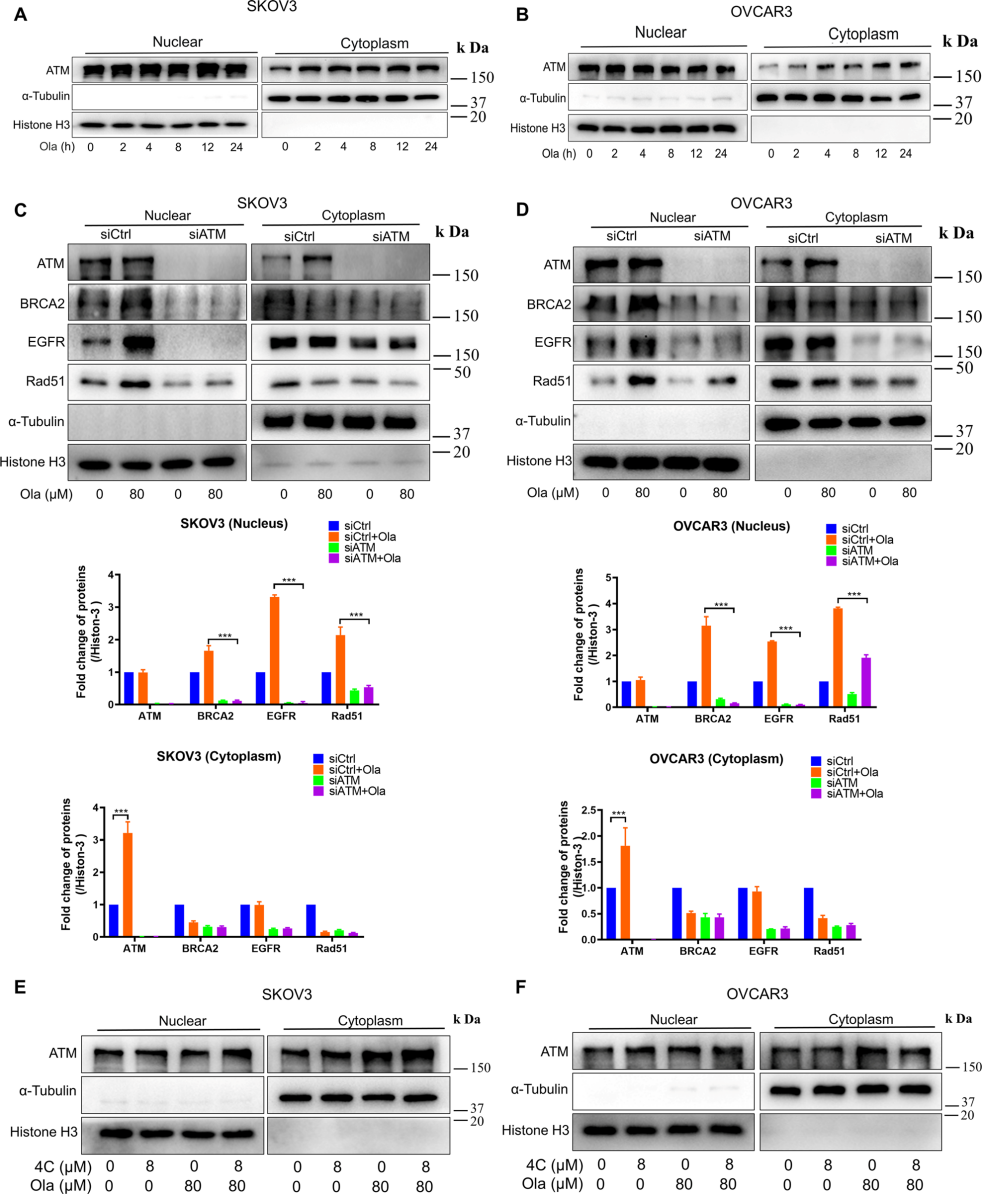
4C had no effect on PARPi-induced export of ATM from the nucleus to the cytoplasm. **A**, **B** SKOV3 and OVCAR3 were subjected to Olaparib (80 μM), and cell lysates were separated into nuclear and cytoplasmic fractions. These fractions were then detected by WB with the indicated antibodies. **C**, **D** SKOV3 and OVCAR3 cells expressing siCtrl or siATM were subjected to Olaparib, and cell lysates were separated into nuclear and cytoplasmic fractions. These fractions were then detected by WB with the indicated antibodies. **E**, **F** SKOV3 and OVCAR3 cells were subjected to Olaparib and **4C** and cell lysates were separated into nuclear and cytoplasmic fractions. These fractions were then detected by western blot with the indicated antibodies. (*n* = 3, ***P < 0.001).

The next question is, after DNA damage induced by PARPi, how does the damage signal get transmitted from the nucleus to the cytoplasm to promote nuclear transfer of EGFR, BRCA2, and Rad51? As we all know, ATM plays a crucial role in maintaining genome stability as an early sensor after DNA damage [24–26]. Also, ATM is activated and transferred to the cytoplasm, where it promotes the activation of pro-survival signaling pathways in response to DNA damage [27, 28]. To test whether the transfer of ATM from the nucleus to the cytoplasm also acts as a signal transmitter after PARPi-induced DNA damage, the distribution of ATM was detected. Our findings showed that the expression of ATM in the cytoplasm increased with no significant difference in the nucleus after Olaparib treatment (Fig. 6A, B). Furthermore, we explored whether ATM plays any role in the nuclear localization of EGFR, BRCA2 and Rad51 after Olaparib treatment. Interestingly, after silencing ATM, Olaparib-induced nuclear localization of these three proteins was reduced compared to the siCtrl group (Fig. 6C, D). In contrast, **4C** had no effect on Olaparib-induced export of ATM from the nucleus to the cytoplasm, indicating that ATM is the upstream signal of EGFR (Fig. 6E, F).

### 4C enhanced the sensitivity of BRCA wild-type OC cells to PARPi in vivo

**Fig. 7:**
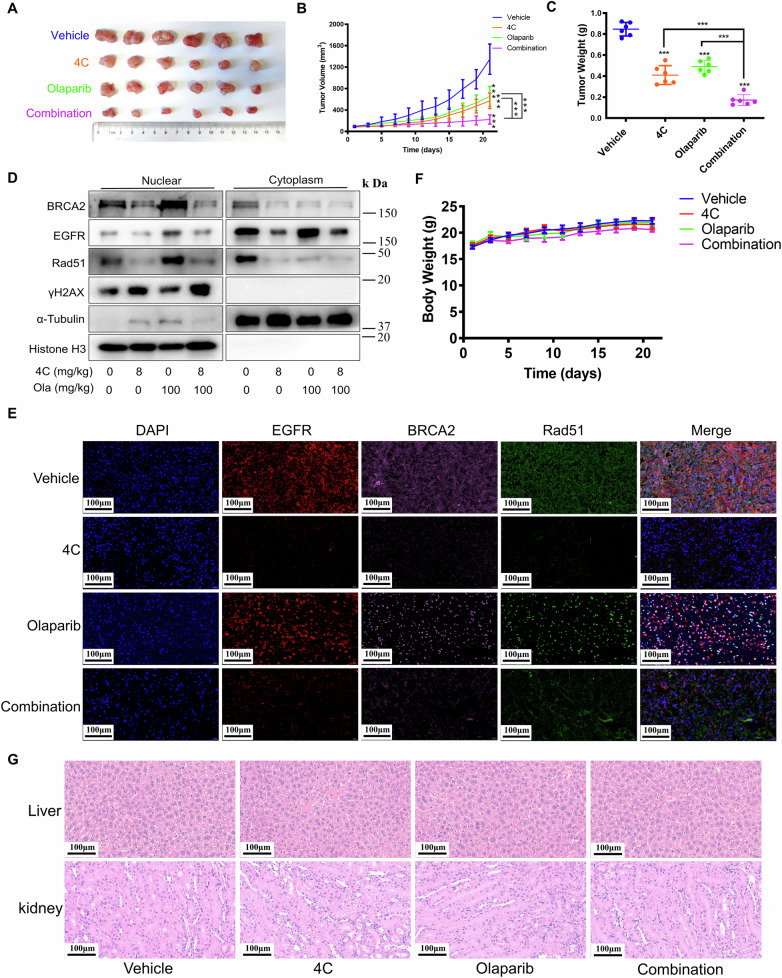
The effect and mechanism of 4C combined with PARPi leads to “synthetic lethality” in vivo of BRCA wild-type OC. **A**–**C** SKOV3 cells were inoculated subcutaneously into the flank of mice. When the tumor volume reached 70–100 mm^3^, mice were treated with **4C** (8 mg/kg i.p. five times a week) and Olaparib (100 mg/kg i.g. five times a week) alone or in combination. Tumor images (**A**), tumor volumes (**B**) and tumor weight (**C**) were then assessed (6 mice/group), ***P < 0.001. **D** Tumor tissues after subjected to **4C** and Olaparib being separated into nuclear and cytoplasmic fractions. These fractions were then detected by WB with the indicated antibodies. **E** The expression of indicated proteins in tumor tissues after being subjected to **4C** and Olaparib were detected by immunofluorescence. **F** The changes in body weight in each group of mice. **G** Representative images of HE analysis for liver and kidney organs of mice. (*n* = 6, ***P < 0.001).

Given the synergistic effects observed between **4C** and PARPi in vitro, we further examined whether **4C** enhanced the sensitivity of PARPi (Olaparib, Niraparib) in vivo. As expected, mice treated with **4C** or PARPi (Olaparib, Niraparib) alone led to a modest decrease in tumor size (Fig. 7A, B and Supplementary Fig. 6A, B). Interestingly, the combination of **4C** and PARPi (Olaparib, Niraparib) resulted in tumors to almost stop growing (Fig. 7A, B and Supplementary Fig. 6A, B), demonstrating a synergistic effect that was superior to monotherapy. Upon completion of the treatment, tumors were collected from the mice. The average weight of tumors in the combination group was notably lower compared to any of the monotherapy groups (Fig. 7C and Supplementary Fig. 6C). These findings suggest that **4C** significantly enhanced the sensitivity of PARPi in vivo. It is well established that cancer metastasis is a major contributor to patient mortality. Therefore, we further investigated whether the combination of **4C** and Olaparib similarly inhibits ovarian cancer metastasis. Consistently, **4C** significantly enhanced the anti-metastatic efficacy of Olaparib, reducing the formation of metastatic nodules (Supplementary Fig. 7).

The harvested tumors were used to measure whether **4C** or PARPi has any effect on the distribution of BRCA2 and Rad51. The results revealed that PARPi promoted the transfer of BRCA2 and Rad51 from cytoplasm to the nucleus in the tumors (Fig. 7D, E and Supplementary Fig. 6D, E), implying that this nuclear import may also be critical for the insensitivity of BRCA wild-type OC cells to PARPi in vivo. Interestingly, we observed that **4C** inhibited PARPi-induced transfer of BRCA2 and Rad51from cytoplasm to nucleus as well (Fig. 7D, E and Supplementary Fig. S6D, E). Importantly, although **4C** improved the sensitivity of OC tumors to PARPi in vivo, neither loss of body weight nor toxicity of liver and kidney in nude mice in all treatment groups was observed (Fig. 7F, G and Supplementary Fig. 6F, G), indicating the presence of excellent safety in these treatments. These results further support the potential clinical application of the combination of **4C** and PARPi, providing hope for the treatment of BRCA wild-type patients.

## Discussion

Although PARPi have shown a dramatic success in the clinical treatment of BRCA-mutated patients, the majority of BRCA wild-type OC do not benefit from these drugs [29, 30]. It is widely recognized that the enhancement of HR is crucial for cell survival and insensitivity of BRCA wild-type cancer cells to PARPi. At the same time, studies show that BRCA2 and Rad51 play the most important roles in HR [31]. We initially consider that the reasons of PARPi insensitivity in BRCA1/2 wild-type OC cells may be related to the promotion of HR by up-regulating the expression of BRCA2 and Rad51, the two critical proteins of HR. Contrary to expectations, we observed that PARPi-induced re-localization of BRCA2 and Rad51 from cytoplasm to nucleus, rather than up-regulation of their expressions, was the critical event in promoting HR and PARPi resistance. However, since BRCA2 and Rad51 also play a vital role in maintaining the genetic stability of normal cells, there is a huge challenge to enhance the sensitivity of PARPi to impair HR by directly inhibiting the nuclear import of BRCA2 and Rad51 [32]. Indeed, several early clinical trials aimed at enhancing PARPi sensitivity by directly inhibiting BRCA2 and Rad51 ultimately failed. Current HR inhibitors targeting upstream DNA damage response pathways, such as the ATM inhibitor AZD0156, have shown promise in preclinical models for sensitizing tumors to PARPi [33]. They may also cause systemic toxicity, potentially limiting their clinical application. Crucially, our study identifies EGFR, which is highly expressed in cancer cells but not in normal cells, as a novel and more tumor-selective regulator of HR and PARPi sensitivity in BRCA1/2 wild-type OC. Interestingly, downregulation of the highly expressed EGFR in cancer cells provides an indirect strategy to suppress HR. This approach represents a comparatively safer therapeutic alternative to direct HR inhibition. Consistent with this advantage, the combination of **4C** and Olaparib demonstrated no increased toxicity in normal cells in vitro or in vivo. Besides, Lee HJ also indicated that EGFR plays a role in mediating HR, promoting cell resistance to radiation-induced DNA damage [34]. However, the specific mechanism by which EGFR regulates HR has not yet been fully elucidated. In this study, we reveal that EGFR regulates HR as a new posttranscriptional modifier of BRCA2 and Rad51, and knocking down EGFR led to the ubiquitination and degradation of BRCA2 and Rad51 to enhance the sensitivity of PARPi in BRCA1/2 wild-type OC both in vitro and in vivo, implying a significant advancement for the use of PARPi in BRCA1/2 wild-type OC patients.

Our laboratory is committed to exploring the antitumor effects of biguanides and their antitumor mechanisms [35–39]. Based on the intermediate derivatization methods, a series of biguanide derivates were obtained, and **4C** was found to have excellent antitumor activity in OC. As we all know, the classical mechanism by which biguanides inhibit tumor cell proliferation is activation of AMPK [40–44]. On the other hand, our laboratory and published studies updated the novel mechanisms [17, 45]. In this study, we demonstrate that EGFR is the target of **4C** through calculating the binding energy of **4C** and EGFR, detecting the changes of EGFR after **4C** treatment and activity of **4C** after knockdown EGFR. Based on the facts that knocking down EGFR could reduce their expression and nuclear transfer of BRCA2 and Rad51 and then enhance the sensitivity of OC to PARPi, we examined the effect of **4C** on PARPi sensitivity. Our results demonstrated that the combination of **4C** and PARPi shows a strong synergy. Mechanistically, Olaparib promoted the binding of BRCA2 and Rad51 to EGFR and then transferred into the nucleus to induce HR, while **4C** inhibited this PARPi-induced HR. Thus, the combination of **4C** and PARPi caused an increase of DNA damage and “synthetic lethality”. It is important to note that the commonly used classical EGFR TKIs in clinical practice, such as Osimertinib, do not enhance the activity of Olaparib in BRCA1/2 wild-type OC in the same way as **4C**. One potential reason for this failure is that these TKIs primarily inhibit the kinase activity of EGFR without reducing its total protein levels. Our data indicate that changes in total EGFR, rather than its kinase activity, are essential for the downregulation of BRCA2, Rad51, and HR activity. Although cetuximab demonstrates some capacity to downregulate the expression of EGFR, BRCA2, and Rad51, **4C** exhibits superior bioactivity and a stronger ability to inhibit HR in OC cells compared to cetuximab. Furthermore, the data presented in this study suggested that silencing E3 ubiquitin ligase c-Cbl, rather than overexpressing deubiquitinating enzyme USP11 rescued **4C**-induced ubiquitination and degradation of BRCA2 and Rad51. On the other hand, compelling studies have shown that inhibiting HR can heighten the responsiveness of cancer cells to DNA damage treatments. Hence, the combination of **4C** with DNA-damaging agents and radiation therapy in BRCA1/2 wild-type cancer is worth further exploration. Additionally, in cancer patients with BRCA1/2 mutations, resistance often develops through various mechanisms, such as secondary mutations to restore the function of BRCA1/2 and Rad51 after PARPi treatment [46, 47]. Therefore, **4C** may re-sensitize PARPi-resistant cells to PARPi by inhibiting restored-HR. Finally, although we validated the efficacy and safety of the combination of **4C** and PARPi in a xenograft tumor model, further investigations, such as in situ tumor models and safety studies in large animals, are deserved.

**Fig. 8:**
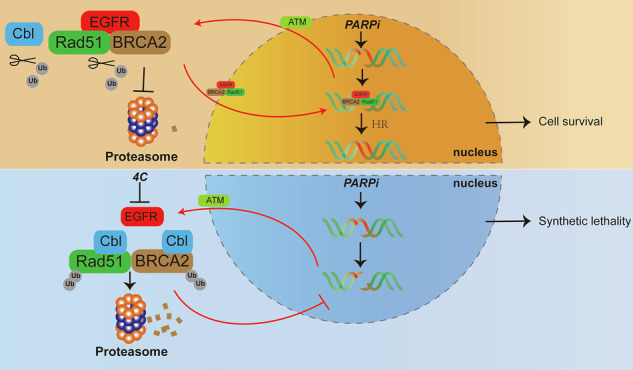
Schematic model. ATM acts as a messenger to transmit PARPi-induced DNA damage signals to the cytoplasm, and then BRCA2 and Rad51 interaction with EGFR and nuclear translocation to activate HR. **4C** enhanced BRCA2 and Rad51 interaction with c-Cbl and then ubiquitination and degradation in the proteasome by targeting EGFR. As such, a combination of **4C** with PARPi leads to “synthetic lethality” of BRCA1/2 wild-type OC (Created with BioRender.com).

In conclusion, we found that EGFR is highly expressed in BRCA1/2 wild-type OC, which is one of the main reasons for the insensitivity to PARPi. Following DNA damage induced by PARPi, ATM transmitted the DNA damage signal to the cytoplasm, where EGFR bound to BRCA2 and Rad51. This complex then translocated to the nucleus, activating HR and promoting the repair of PARPi-induced DNA damage. This repair mechanism is a key factor contributing to PARPi insensitivity in BRCA1/2 wild-type OC. Interestingly, **4C** enhanced BRCA2 and Rad51 interaction with c-Cbl and then ubiquitination and degradation in the proteasome, thereby inhibiting HR to increase the sensitivity of BRCA wild-type OC to PARPi by downregulation of EGFR (Fig. 8). Thus, **4C** can enhance the sensitivity of BRCA1/2 wild-type OC cells with high EGFR expression to PARPi without causing toxicity to normal cells with low EGFR expression.

## Supplementary information

Supplementary Information

Original Data File

## Data Availability

The datasets generated during the current study are available from the corresponding author on reasonable request.
