# Supplementary material for: Repression of EGFR by new biguanide 4C potentiated ovarian cancer to PARP inhibitors through down-regulation of BRCA2 and Rad51
Source: Cell Death Dis. 2026 Mar 18;17(1):317. doi: 10.1038/s41419-026-08556-w (PMC13039286; doi:10.1038/s41419-026-08556-w)
Supplement: Supplementary file 1 — Supplementary Information [file 41419_2026_8556_MOESM1_ESM.docx]

**Supplementary Information**

Repression of EGFR by new biguanide 4C potentiated ovarian cancer to PARP inhibitors through down-regulation of BRCA2 and Rad51

Di Xiao^1,2,3^, Jia Yao^1,3^, Xin Yang^1^, Yijun Xie^1^, Xiaochen Zhou^1^, Duo Li^1^, Mei Peng^1^, Wei Wang^2*^, Hui Zou ^1*^, Xiaoping Yang^1*^

^1^Key Laboratory of Chemical Biology & Traditional Chinese Medicine Research of Ministry of Education, Key Laboratory of Study and Discovery of Small Targeted Molecules of Hunan Province, Engineering Research Center of Reproduction and Translational Medicine of Hunan Province, Key Laboratory of Protein Chemistry and Developmental Biology of Fish of Ministry of Education, Institute of Interdisciplinary Studies, Cancer Institute, School of Pharmaceutical Sciences, Health Science Center, Hunan Normal University, Changsha, Hunan, China

^2^TCM and Ethnomedicine Innovation and Development International Laboratory, Innovative Material Medical Research Institute, School of Pharmacy, Hunan University of Chinese Medicine, Changsha, China. ^3^These authors contributed equally: Di Xiao, Jia Yao.

*Corresponding authors: Tel/Fax: 86-137-8710-8416.

E-mail addresses：[wangwei402@hotmail.com](mailto:wangwei402@hotmail.com) (W. Wang), zouhui@hunnu.edu.cn (H. Zou), xiaoping.yang@hunnu.edu.cn (X. Yang)

**Contents**

Supplementary Figures……………………………………………………………3-14

Supplementary Tables……………………………………………………………15-17

**SFig. 1**


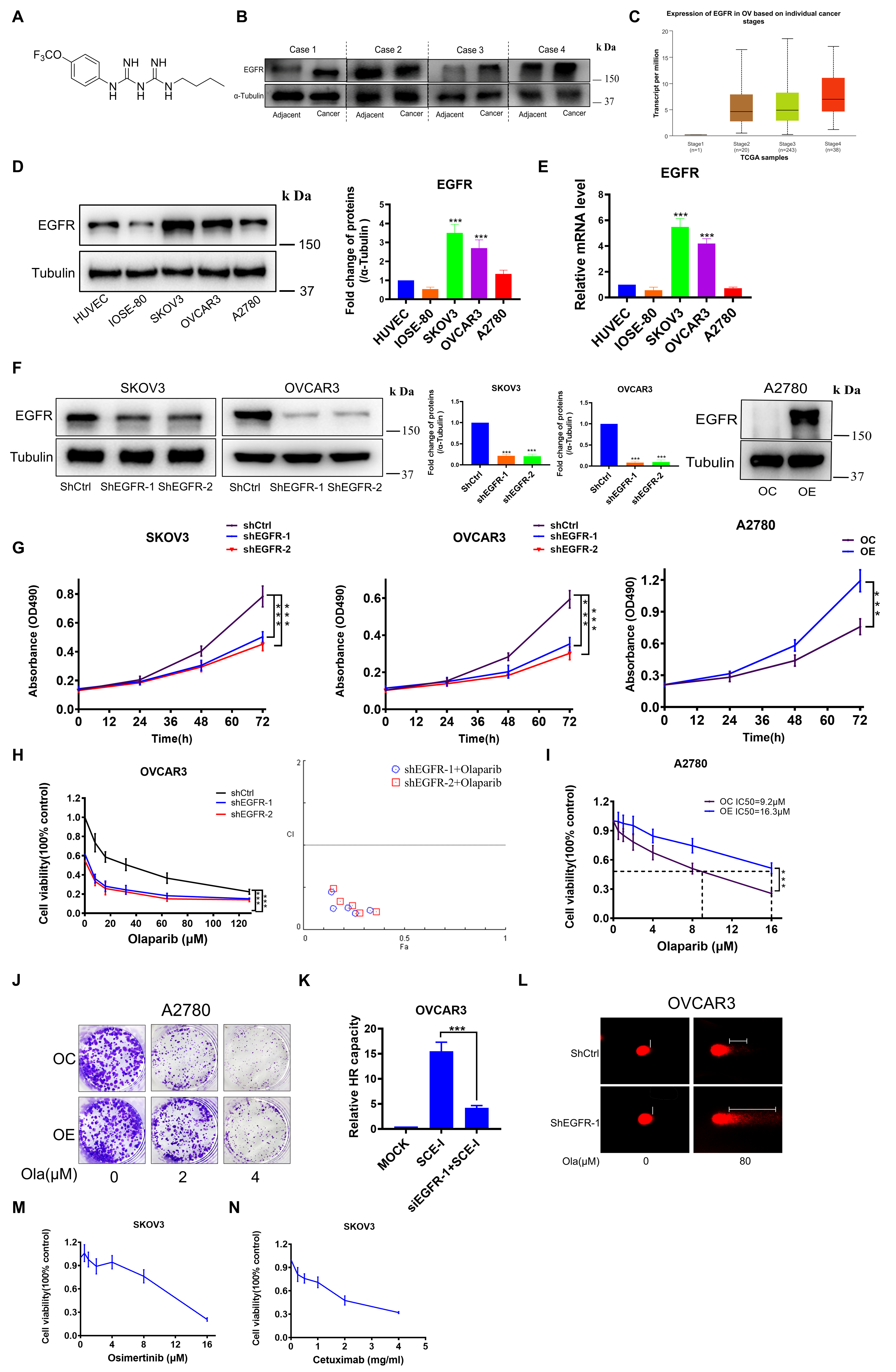


**SFig.1. The effect of EGFR** **expression on the sensitivity of OC cells to Olaparib.** (A) Chemical structure of **4C**. (B) The expression of EGFR were detected by WB. (C) EGFR expression in OC and normal tissues using publicly available datasets. (D, E) The protein (D) and mRNA expression (E) of EGFR in different types of OC cell lines, HUVEC and IOSE-80. (F) The effect of silencing or overexpressing EGFR was detected by WB. (G) The proliferation of shEGFR cells and Overexpression EGFR (OE) cells were detected by MTT assay. (H The cell viability of OVCAR3 after Olaparib treatment was evaluated by MTT. (I, J) A2780 cells transfected with empty vector (OC) or overexpressed EGFR (OE) and then treated with Olaparib. The cell viability was evaluated by MTT (I) and colony formation (J) assay. (K) Cells transfected with siCtrl or siEGFR and HR-specific repair of DNA damage was analyzed using flow cytometer. (L) Cells were treated with Olaparib for 24 h and the degree of DNA damage was measured by comet assay. (M-N) The cell viability of SKOV3 treated with Osimertinib (M) and Cetuximab (N) were evaluated by MTT assay. Ola, Olaparib. Osi, Osimertinib. (n=3, ns, no significant difference, ***P<0.001).

**SFig. 2**


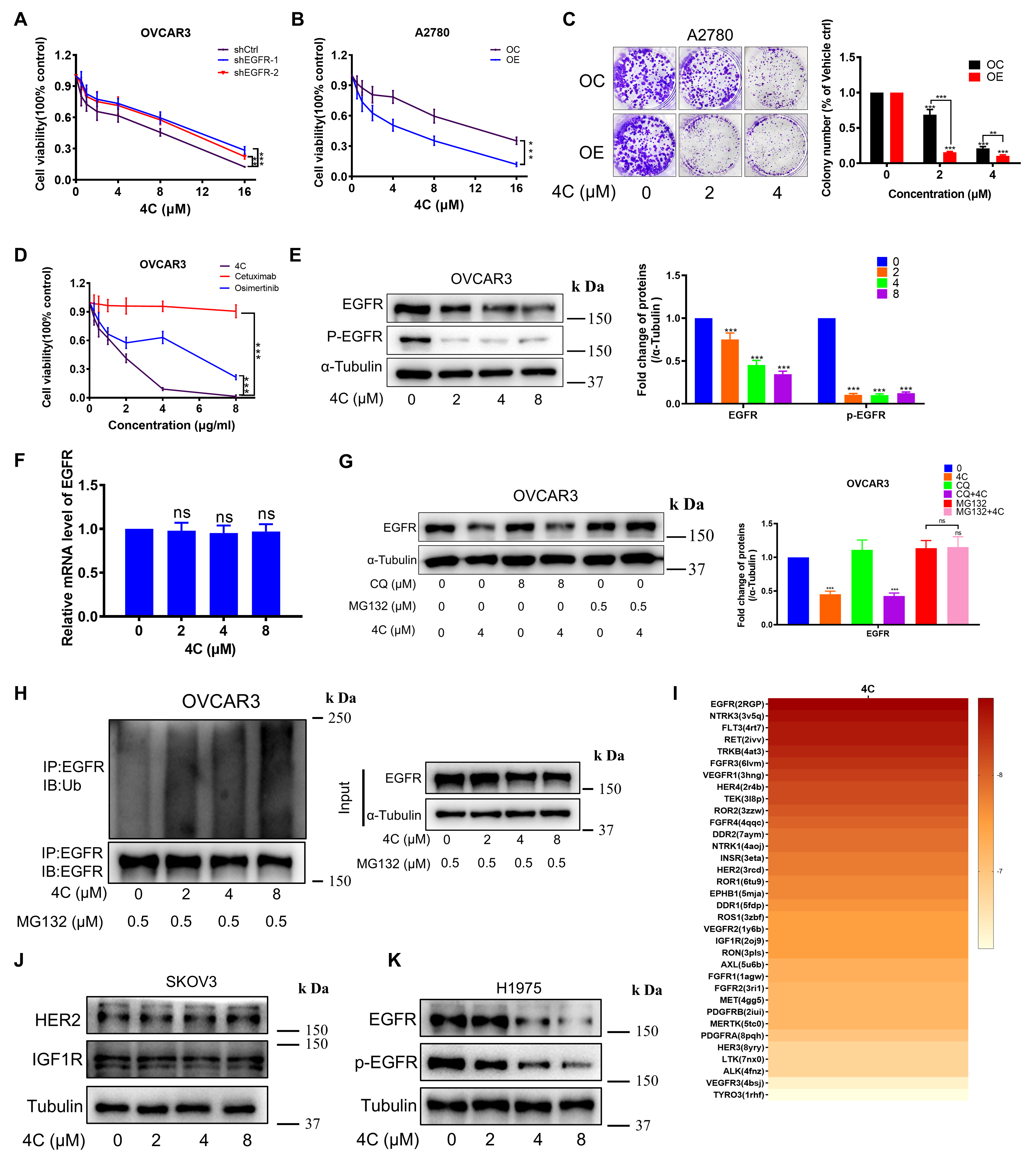


**SFig.2. 4C inhibited OC cell proliferation by targeting EGFR.** (A) The cell viability after **4C** treatment were evaluated by MTT assay. (B, C) A2780 cells transfected with empty vector (OC) or overexpressed EGFR (OE) and then treated with **4C**. The cell viability was evaluated by MTT (B) and colony formation (C) assay. (D) The cell viability after indicated drugs treatment were evaluated by MTT assay. (E, F) OVCAR3 were starved for 6h and treated with **4C** for 24h, and the changes of the indicated proteins or mRNA were analyzed by WB or RT-PCR. (G) OVCAR3 were starved for 6h and then pretreated with chloroquine or MG132 for 12h, and cells were then treated with **4C** for 12 hours. The changes of EGFR were detected by WB. (H) OVCAR3 were starved for 6h and then pretreated with MG132 for 12h, and cells were then treated with **4C** for 12 hours and lysed to immunoprecipitation using anti-EGFR antibody, followed by western blot with indicated antibodies. (I) The binding energy of 4Cs to kinase receptors was calculated by Vina docking. (J-K) Cells were starved for 6h and treated with **4C** for 24h, and the changes of the indicated proteins were analyzed by WB. (n=3, ns, no significant difference, **P<0.01, ***P<0.001).

**SFig. 3**


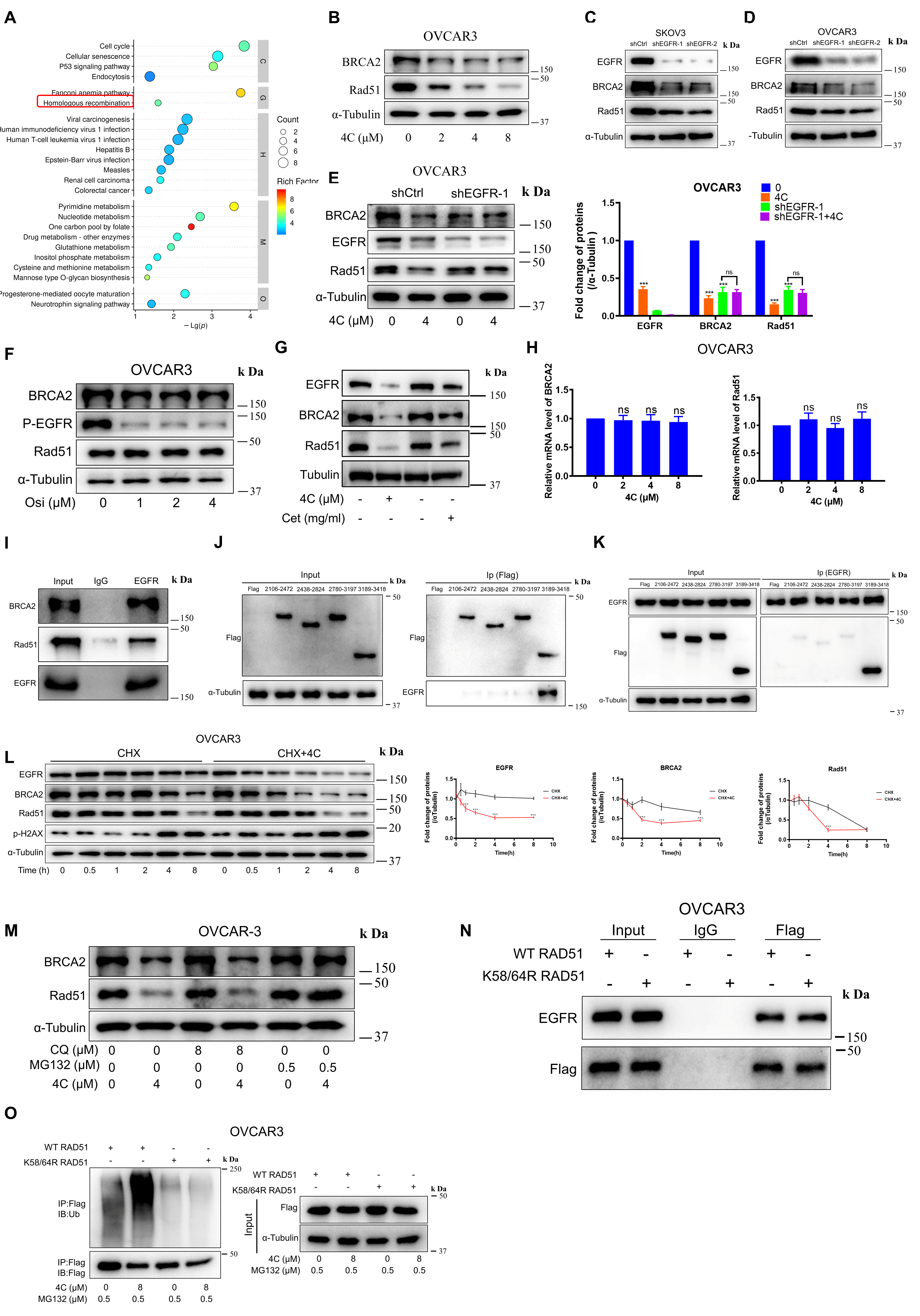


**SFig.3. 4C promoted ubiquitination and degradation of BRCA2 and Rad51.** (A) GO pathway enrichment analysis of downregulated proteins after **4C** treatment. (B) OVCAR3 were starved for 6h and treated with the **4C** for 24h, and the changes of the indicated proteins were analyzed by WB. (C, D) Cells were transfected with lentiviral vectors and screened by puromycin. The changes of the indicated proteins were analyzed by WB. (E, F) OVCAR3 were starved for 6h and treated with **4C** or Osimertinib for 24h, and the changes of the indicated proteins were analyzed by WB. (G) SKOV3 cells were treated with either **4C** or Cetuximab at their respective IC_50_ concentrations for 24 h, and the levels of the indicated proteins were analyzed by WB. (H) OVCAR3 were starved for 6h and treated with **4C** for 24h, and the changes of the indicated mRNA were analyzed by RT-PCR. (I) OVCAR3 were lysed to immunoprecipitation using anti-EGFR antibody, followed by WB with indicated antibodies. (J, K) OVCAR3 transfected with Flag- BRCA2 truncate and then lysed to immunoprecipitation using anti-Flag (J) or anti-EGFR (K) antibody, followed by WB with indicated antibodies. (L) OVCAR3 cells were treated with CHX (20µg ml^-1^) or CHX (20µg ml^-1^) + **4C** (8µM). The changes of indicated proteins were detected by WB. (M) OVCAR3 were starved for 6h and then pretreated with chloroquine or MG132 for 12 hours, and cells were then treated with **4C** for 12 hours. The changes of indicated proteins were detected by WB. (N) CO-IP analysis of indicated protein interaction. (O) CO-IP analysis of the ubiquitination of Rad51. (n=3, ns, no significant difference, *P<0.05, ***P<0.001).

**SFig. 4**


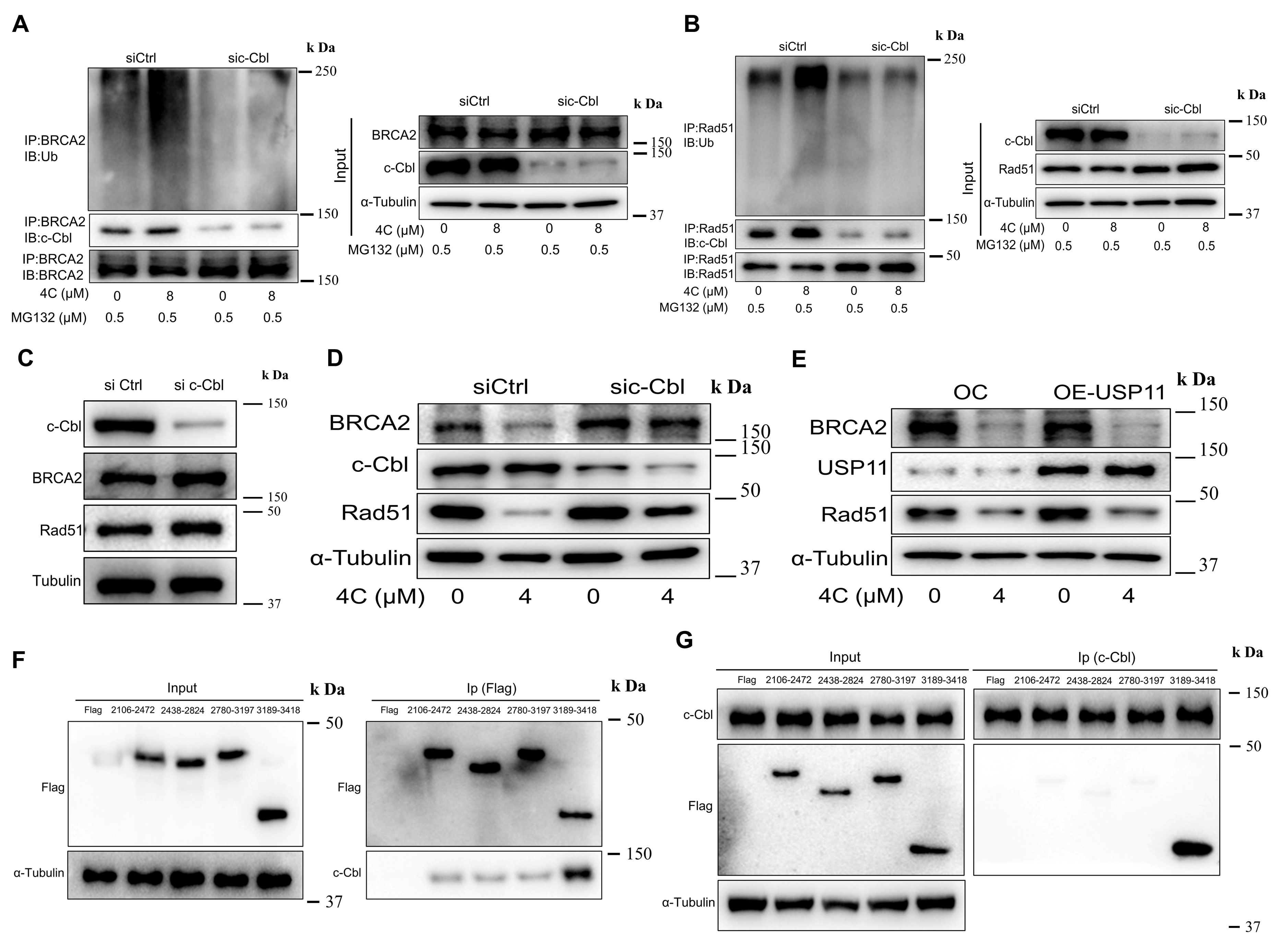


**SFig.4. EGFR stabilized BRCA2/Rad51 via competitive inhibition of Cbl-mediated ubiquitination.** (A, B) OVCAR3 were transfected with siCtrl or sic-Cbl. After 48h, cells were starved for 6h and treated with **4C** for 12h and then lysed to immunoprecipitation using anti-BRCA2 (A) or anti-Rad51 (B) antibody, followed by Western blot with Ub. (C) Cells were transfected with sic-Cbl. After 72h, cells were lysed for protein extraction, and the levels of the indicated proteins were analyzed by WB. (D, E) OVCAR3 cells were transfected with USP11 plasmid or sic-Cbl. After 48h, cells were starved for 6h and treated with **4C** for 24h, and the changes of the indicated proteins were analyzed by WB. (F, G) OVCAR3 cells transfected with Flag-BRCA2 (2106-2472), Flag-BRCA2 (2438-2824), Flag-BRCA2 (2780-3197), Flag-BRCA2 (3189-3418), or empty vector were lysed to immunoprecipitation using anti-Flag (F) or anti-Cbl (G) antibody, followed by WB with indicated antibodies.

**SFig. 5**


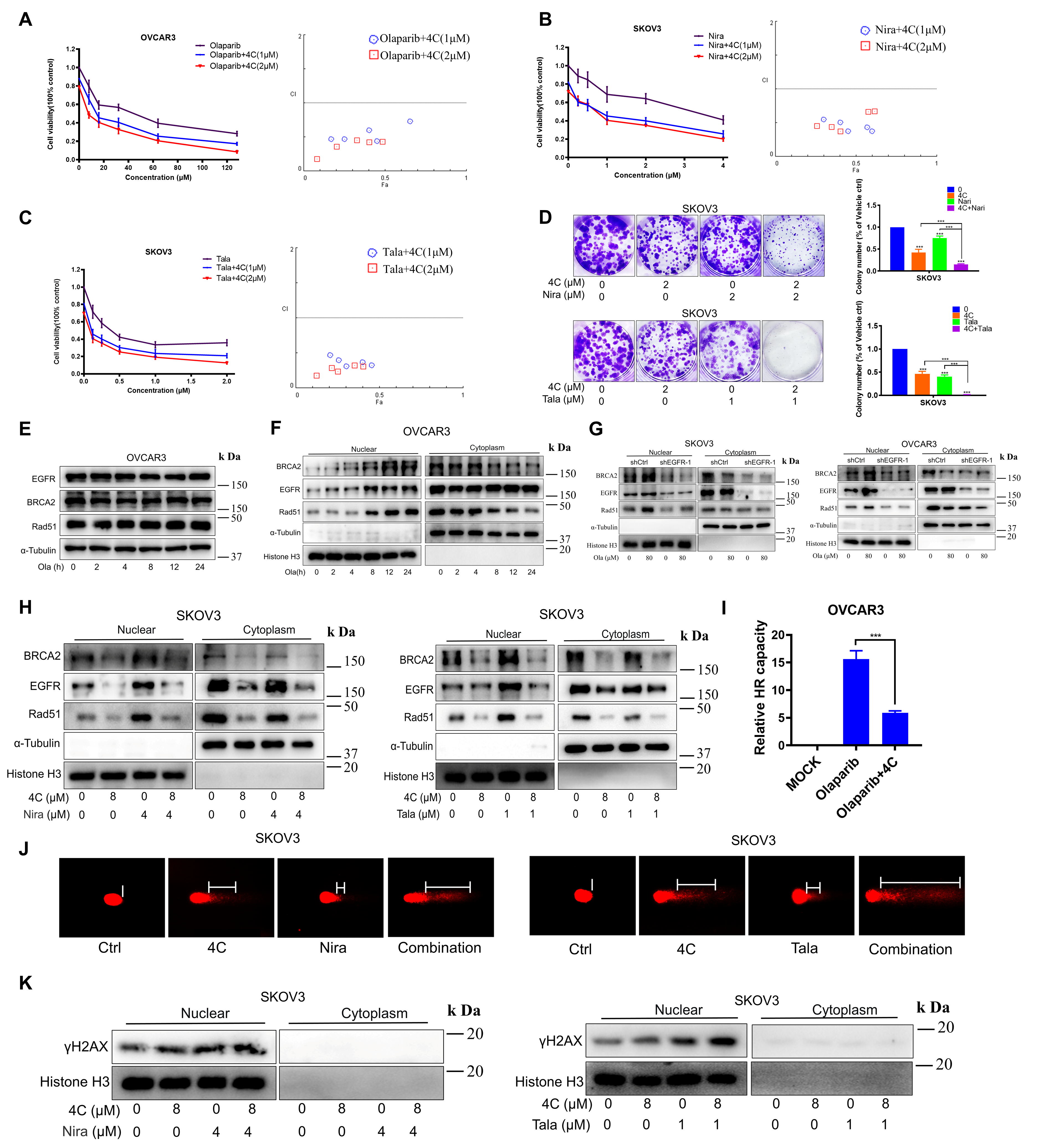


**SFig.5. The effect and mechanism of 4C combined with PARPi leads to "synthetic lethality" in BRCA wild-type OC.** (A-D) The cell viability after **4C** and PARPi (Olaparib, Niraparib, Talazopanib) treatment were evaluated by MTT and Colony formation assay. The combination index (CI) was calculated using CompuSyn software. (E) WB analysis of indicated proteins after Olaparib (80µM) treatment for indicated times. (F) OVCAR3 cells were treated with Olaparib and cell lysates were collected and separated into nuclear and cytoplasmic fractions. These fractions were then detected by WB. (G) Cells were treated with Olaparib and cell lysates were separated into nuclear and cytoplasmic fractions. These fractions were then detected by WB. (H) SKOV3 cells were treated with **4C** and PARPi (Niraparib or Talazopanib) and cell lysates were separated into nuclear and cytoplasmic fractions. These fractions were then detected by WB. (I) OVCAR3 were treated with Olaparib (80μM) and **4C** (8μM) for 24 h and HR was analyzed using HR assay. (J, K) Cells were treated with PARPi (Niraparib or Talazoparib)) and **4C** for 24h and the degree of DNA damage was measured by comet assay and WB. Ola, Olaparib. Nira, Niraparib. Tala, Talazoparib. (n=3, ***P<0.001).

**SFig. 6**


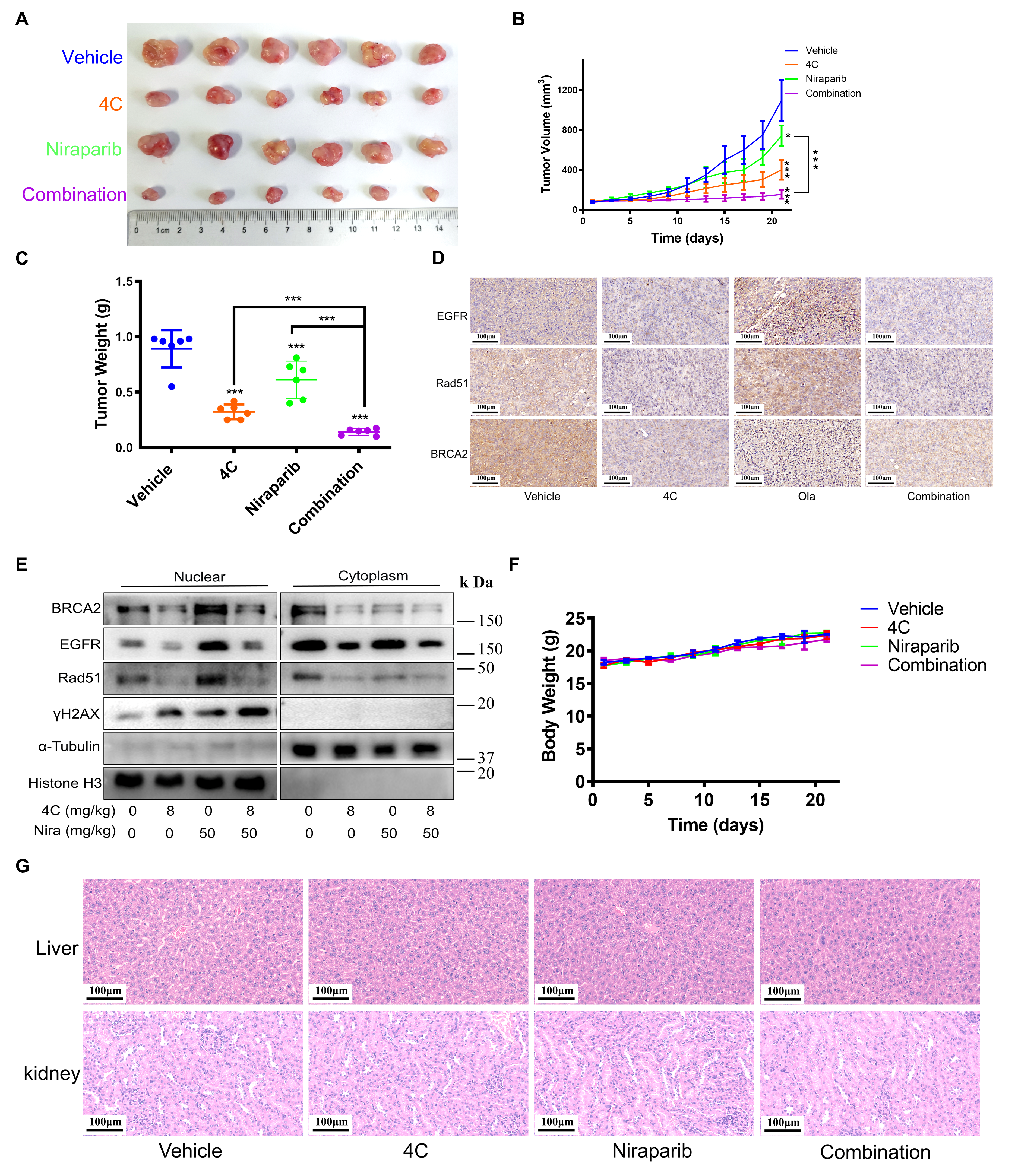


**SFig.6. The effect and mechanism of 4C combined with PARPi leads to "synthetic lethality" in vivo of BRCA wild-type OC.** (A-C) SKOV3 cells were inoculated subcutaneously into the flank of mice. When the tumor volume reached 70–100 mm^3^, mice were treated with **4C** (8 mg/kg i.p. five times a week) and Niraparib (50 mg/kg i.g. five times a week) alone or in combination. Tumor images (A), tumor volumes (B) and tumor weight (C) were then assessed (6 mice/group), *P<0.05, ***P<0.001. (D) The expression of indicated proteins in tumor tissues after treated with **4C** and Olaparib alone or in combination were detected by immunohistochemistry. (E) Tumor tissues after treated with **4C** and Niraparib alone or in combination separated into nuclear and cytoplasmic fractions. These fractions were then detected by western blot with the indicated antibodies. (F) The changes of body weight in each group of mice. (G) Representative images of HE analysis for liver and kidney organs of each group mice. Nira, Niraparib. Data are representative of three independent experiments except for animal experiments. Error bars represent means ± SD from triplicate experiments (*P<0.05, ***P<0.001).

**SFig. 7**


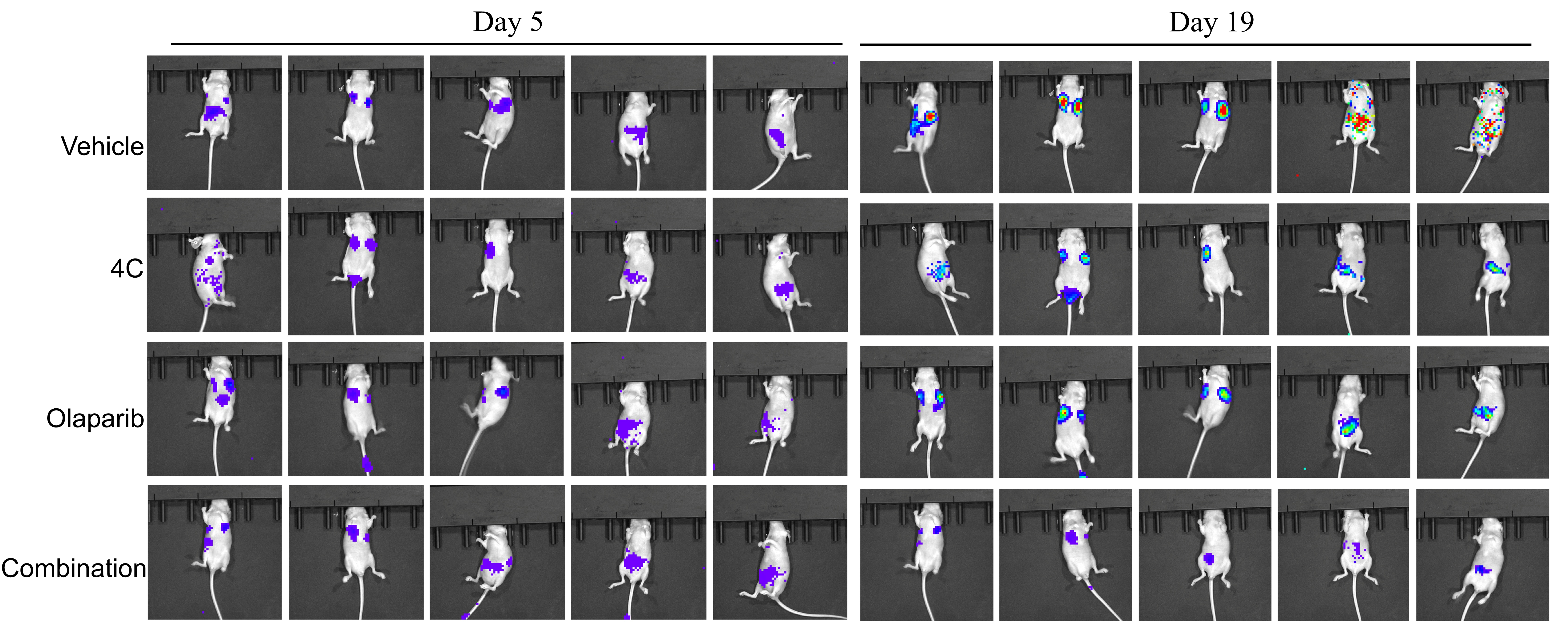


**SFig.7. The effect of 4C combined with olaparib on ovarian cancer migration.** Establishment of metastasis model and treatment regimen: SKOV3-luc cells (2 × 10⁶) were injected via tail vein to establish a tumor metastasis model. Metastasis formation was monitored by bioluminescence imaging (BLI) 5 days post-injection (150 mg/kg D-luciferin, i.p.), and mice were assigned to groups based on baseline imaging. Mice received daily treatments with 4C or Olaparib. After 14 days of treatment, metastatic lesions were re-evaluated by BLI to assess the effect of the 4C and Olaparib combination.

**Supplementary Table 1. Clinical characteristics of the patients**

| **Case 1** | Age | 56 |
| --- | --- | --- |
|  | Classification | Highly differentiated serous carcinoma |
|  | Staging | Ⅲc |
| **Case 2** | Age | 52 |
|  | Classification | poorly differentiated adenocarcinoma |
|  | Staging | / |
| **Case 3** | Age | 47 |
|  | Classification | High-grade serous ovarian cancer |
|  | Staging | ⅢB |
| **Case 4** | Age | 55 |
|  | Classification | High-grade serous ovarian cancer |
|  | Staging | Ⅲc |

**Supplementary Table 2. Chemicals and primary antibodies used in this study**

| **Name** | **Supplier** | **Cat no.** |
| --- | --- | --- |
| Olaparib | Selleck | S1060 |
| Niraparib | Selleck | S2741 |
| Talazoparib | Selleck | S7048 |
| Osimertinib | Selleck | S7297 |
| Chloroquine | Medchemexpress | HY-17589AS |
| MG132 | Selleck | S2619 |
| Cycloheximide | Selleck | S7418 |
| LIPO6000 Transfection Reagent | Beyotime | C0526 |
| EGFR | Cell Signaling Technology | 4267 |
| p-EGFR | Cell Signaling Technology | 3777 |
| α-Tubulin | Proteintech | 11224-1-AP |
| Histone H3 | Proteintech | 17168-1-AP |
| BRCA2 | Abcam | AB123491 |
| Rad51 | Cell Signaling Technology | 8875 |
| Flag | Proteintech | 66008-4-lg |
| Cbl | Cell Signaling Technology | 8447 |
| ATM | Abcame | ab199726 |
| p-H2AX | Cell Signaling Technology | 9718 |
| Ubiquitin | Santa Cruz biotechnology | sc-8017 |

**Supplementary Table 3. Primers used in this study**

| Gene | Forward Primer | Reverse Primer |
| --- | --- | --- |
| EGFR | GAATTCGATGATCAACTCACGG | ACCCATATGTACCATCGATGTC |
| BRCA2 | GTCTTTCCACAGCCAGGCAGTC | GAGAACACGCAGAGGGAACTTGG |
| Rad51 | GCCCTTTACAGAACAGACTACT | TTGAGCTACCACCTGATTAGTG |
| GAPDH | CAAGGTCATCCATGACAACTTTG | GTCCACCACCCTGTTGCTGTAG |

**Supplementary Table 4. The targeting oligos of EGFR, c-Cbl and ATM.**

| siRNA | 5'-3' |
| --- | --- |
| EGFR | CATCAGTGGCGATCTCCACAT |
| c-Cbl | CCUCUCUUCCAAGCACUGA |
| ATM | GTGATAGATAACAAGGATA |

**Supplementary Table 5. The targeting oligos of EGFR.**

| Sh EGFR-1 | CATCAGTGGCGATCTCCACAT |
| --- | --- |
| Sh EGFR-2 | CGCAAAGTGTGTAACGGAATA |
