# Supplementary material for: Repression of EGFR by new biguanide 4C potentiated ovarian cancer to PARP inhibitors through down-regulation of BRCA2 and Rad51
Source: Cell Death Dis. 2026 Mar 18;17(1):317. doi: 10.1038/s41419-026-08556-w (PMC13039286; doi:10.1038/s41419-026-08556-w)

Figure 1L

EGFR p-EGFR α-Tubulin

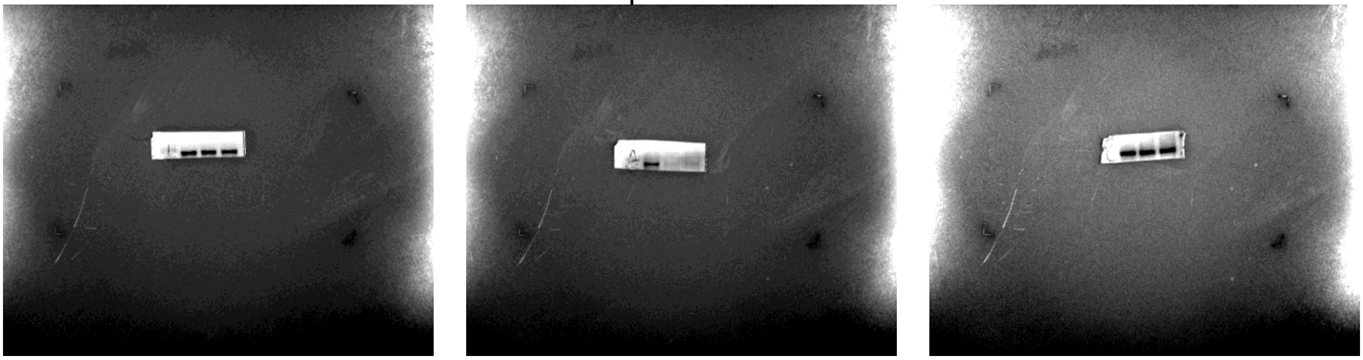

Figure 1O

EGFR p-EGFR α-Tubulin

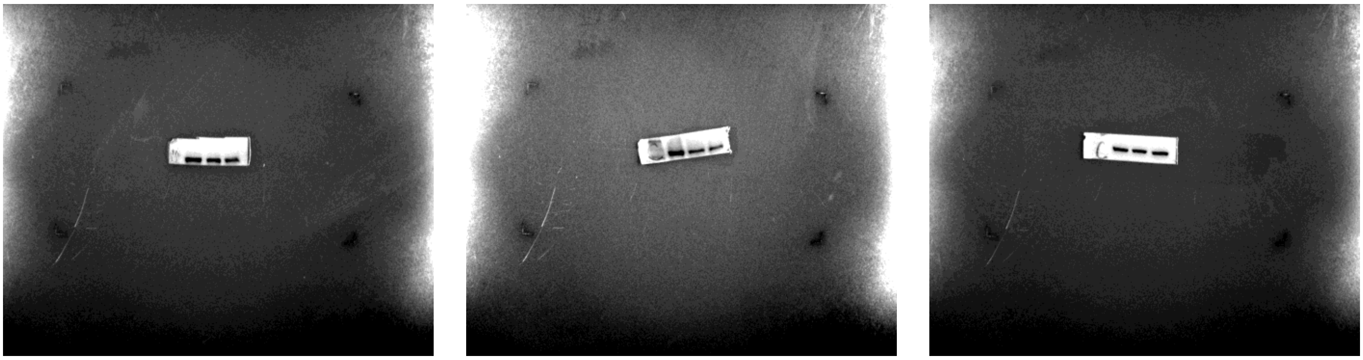

Figure 2I

EGFR

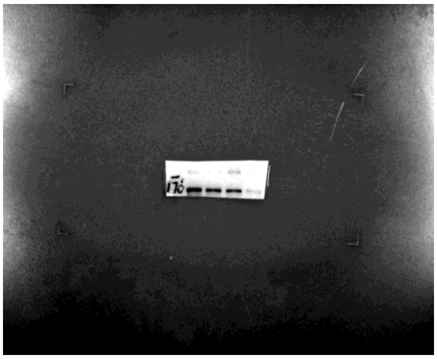

p-EGFR

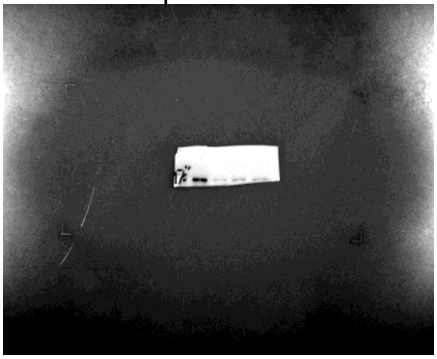

α-Tubulin

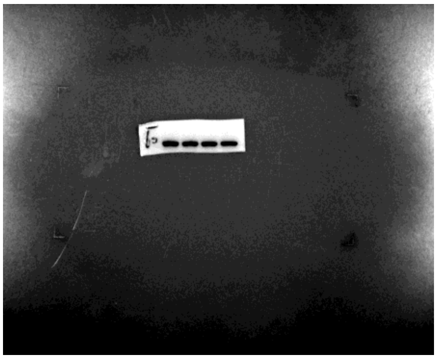

Figure 2K

EGFR

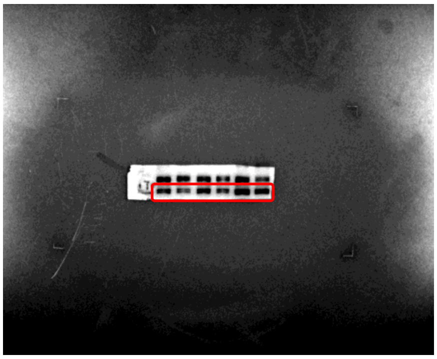

α-Tubulin

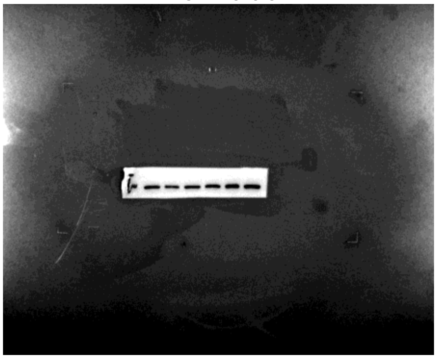

Figure 2L

Ub

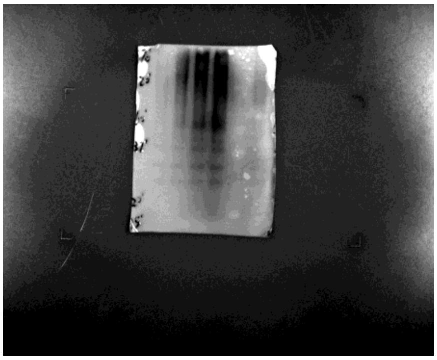

EGFR (IP)

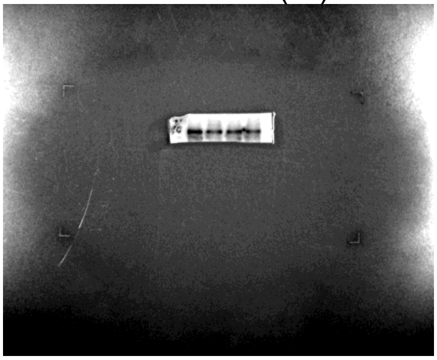

EGFR (Input)

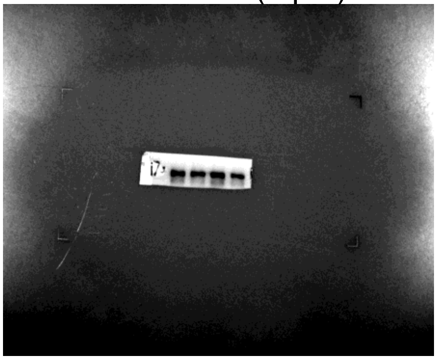

α-Tubulin

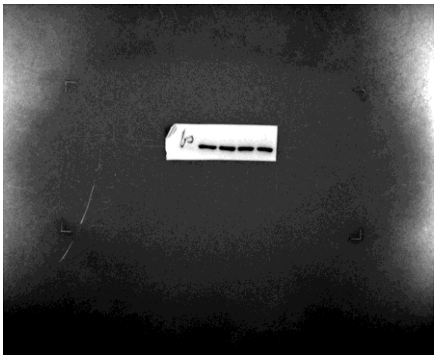

Figure 3C

BRCA2

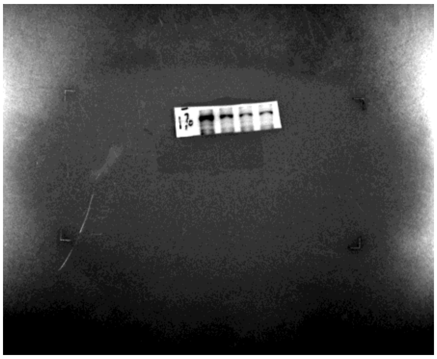

Rad51

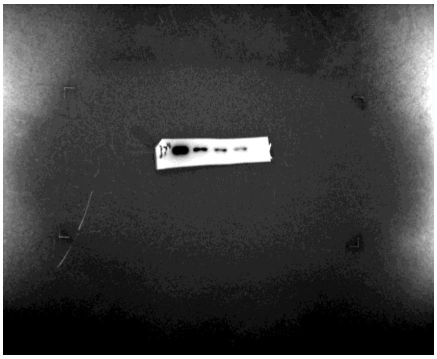

α-Tubulin

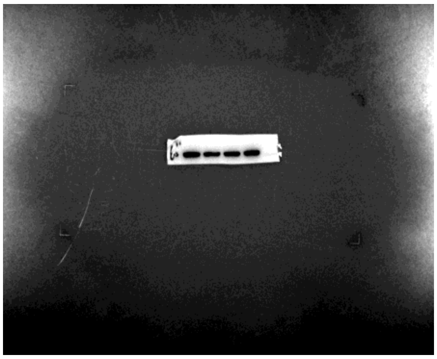

Figure 3D

BRCA2

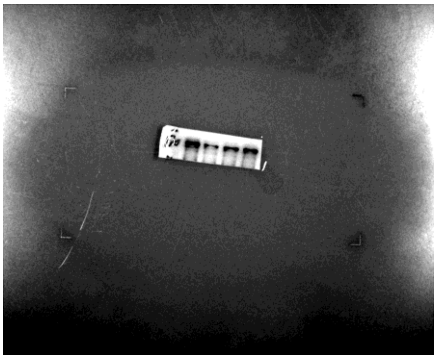

EGFR

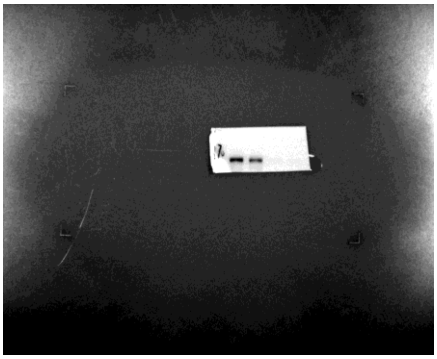

Rad51

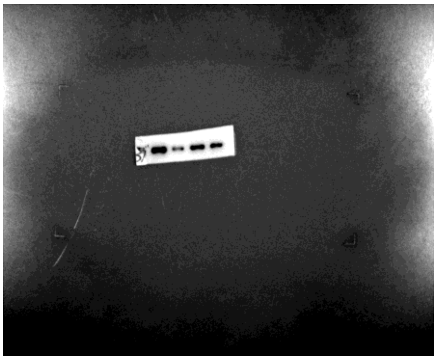

α-Tubulin

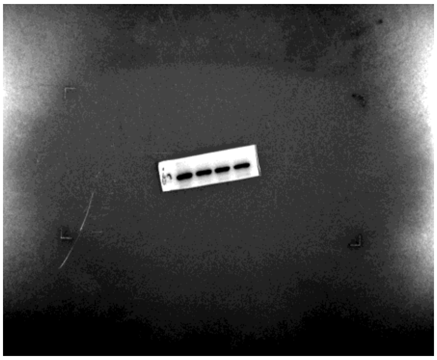

Figure 3E

BRCA2

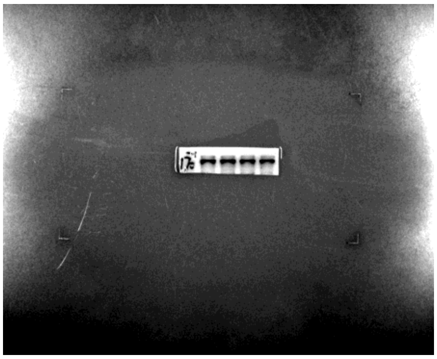

p-EGFR

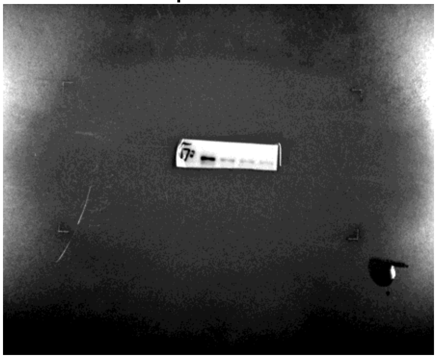

Rad51

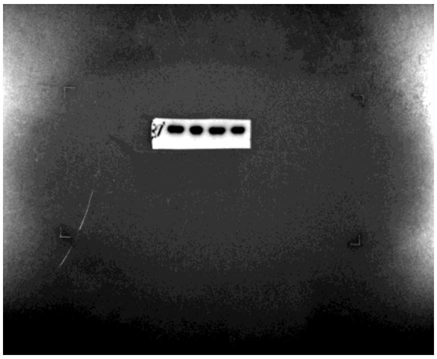

α-Tubulin

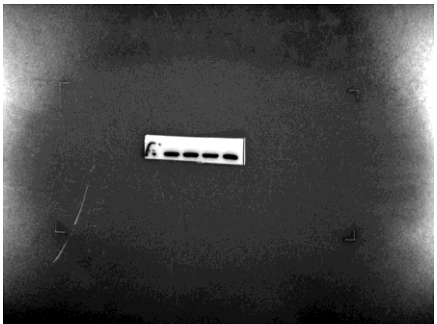

Figure 3G

BRCA2

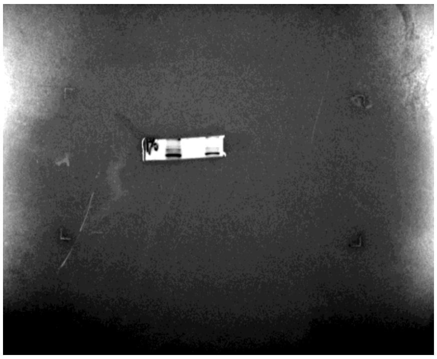

Rad51

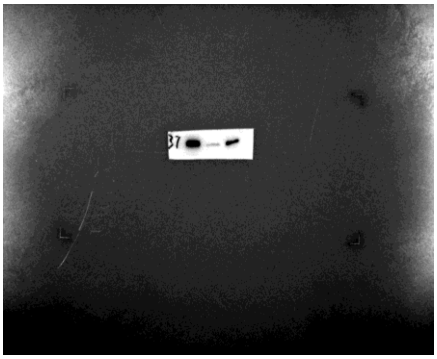

EGFR

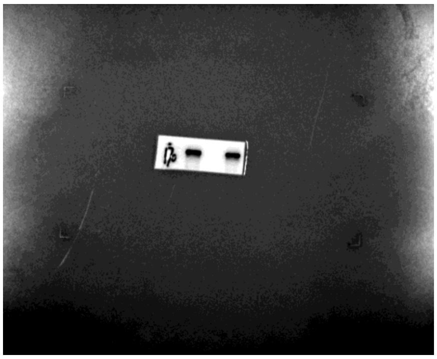

Figure 3H

Flag (Input)

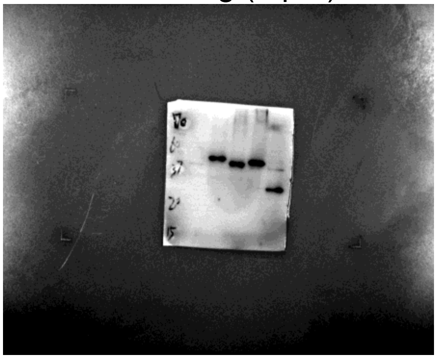

α-Tubulin

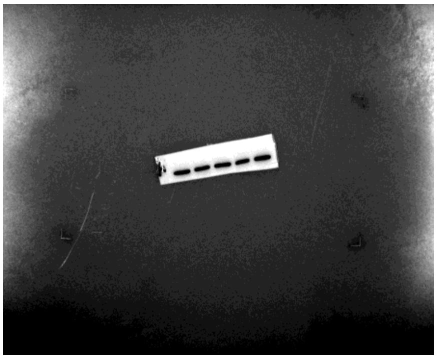

Flag (IP)

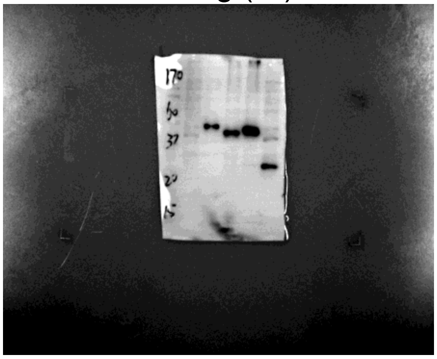

EGFR

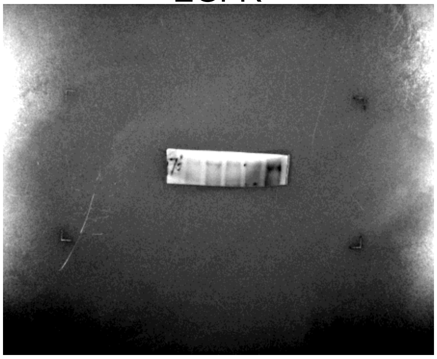

Figure 3I

EGFR (Input)

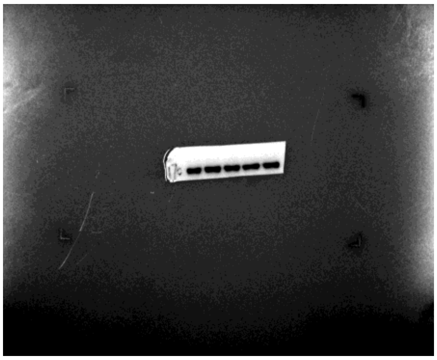

Flag (Input)

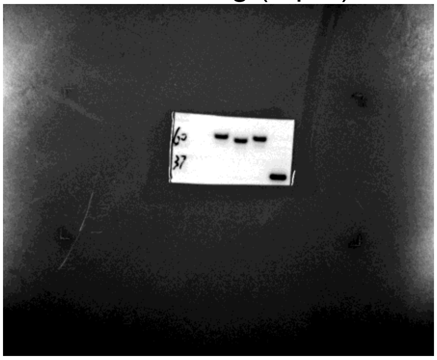

α-Tubulin

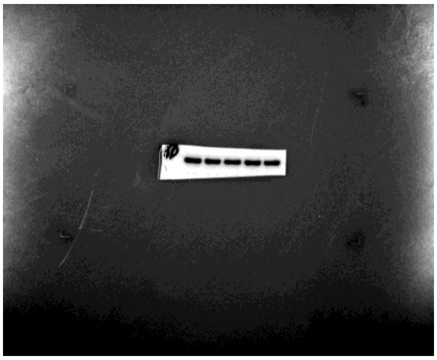

EGFR (IP)

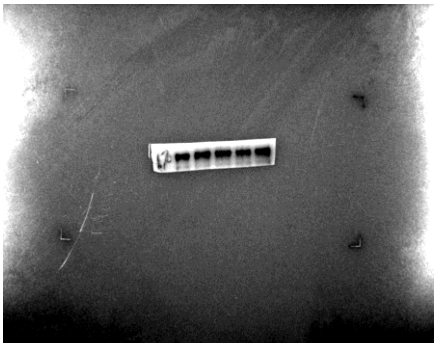

Flag (IP)

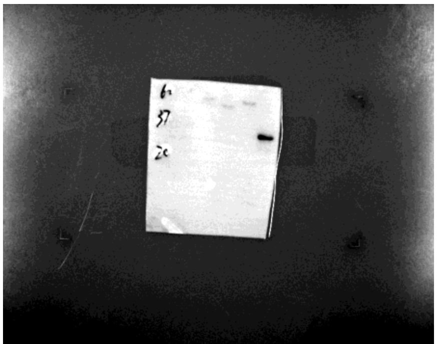

Figure 3J

EGFR

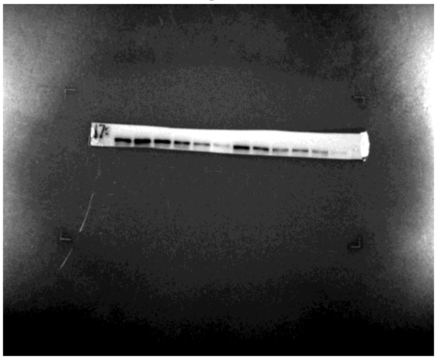

BRCA2

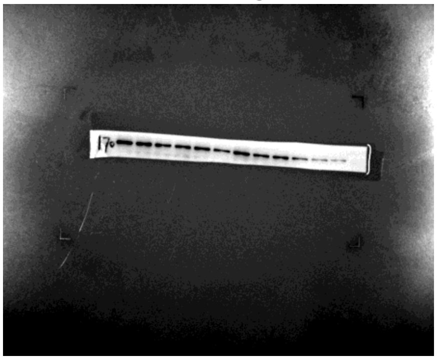

Rad51

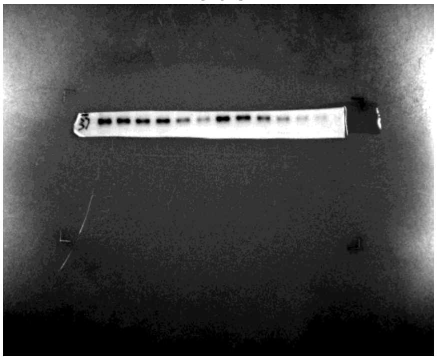

γH2AX

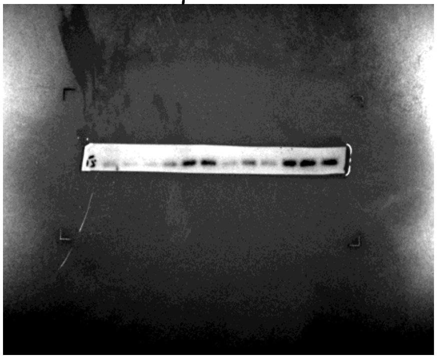

α-Tubulin

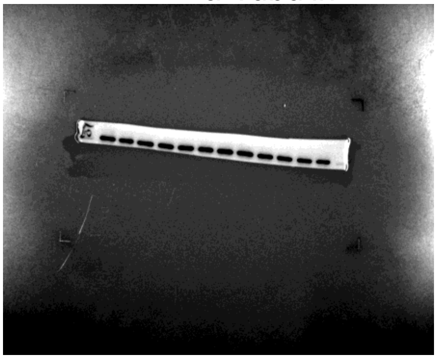

Figure 3I

EGFR

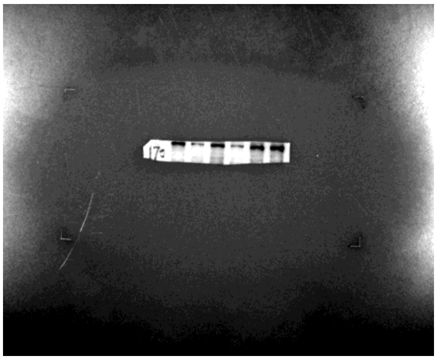

Rad51

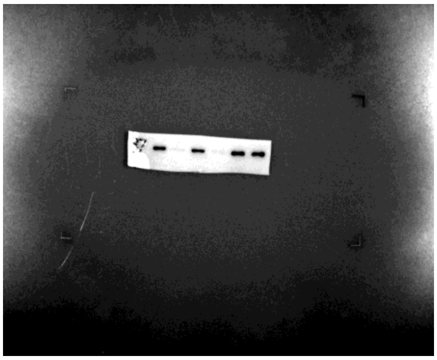

α-Tubulin

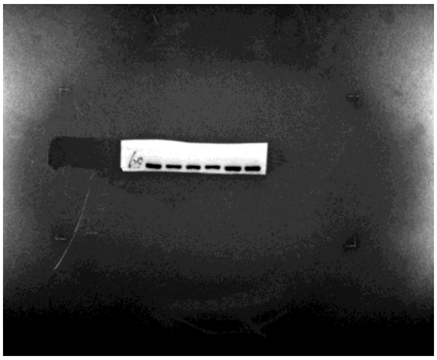

Figure 3L

Ub (BRCA2)

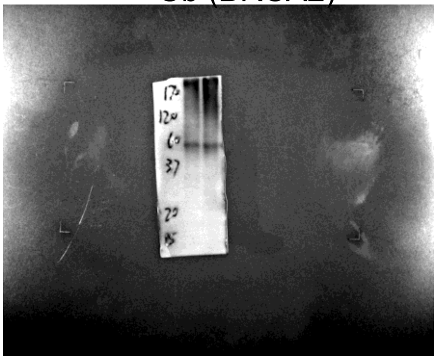

BRCA2 (IP)

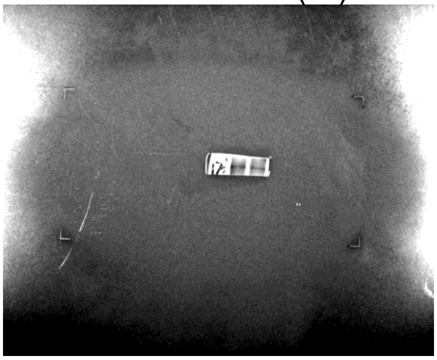

BRCA2 (Input)

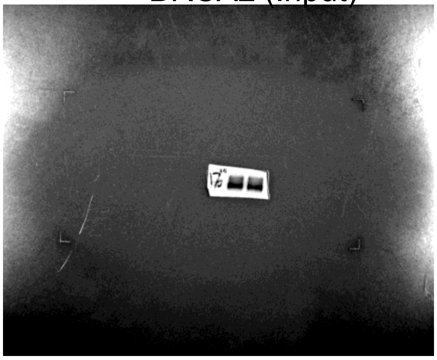

α-Tubulin

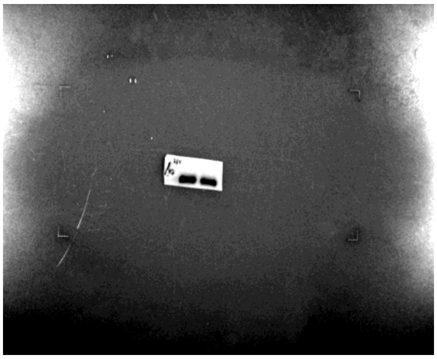

Ub (Rad51)

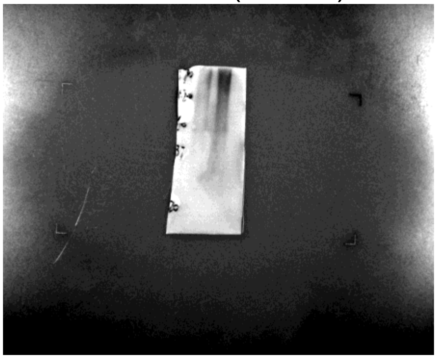

Rad51 (IP)

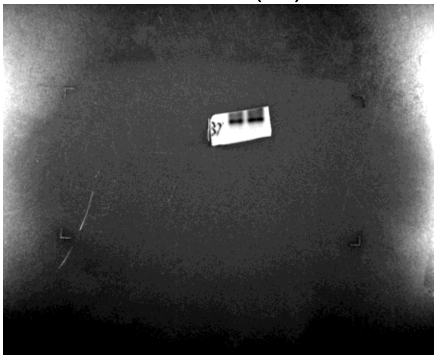

Rad51 (Input)

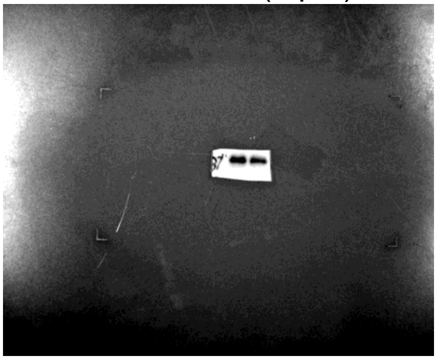

α-Tubulin

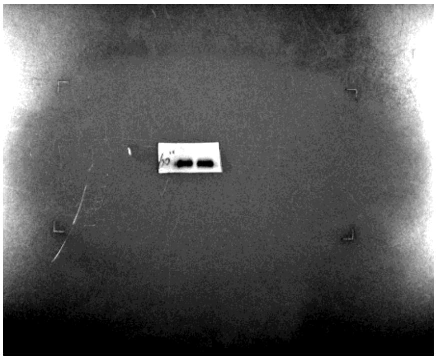

Figure 3M

EGFR

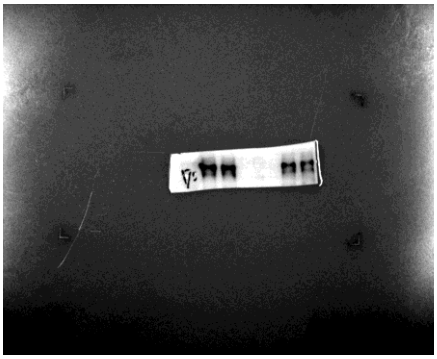

Flag

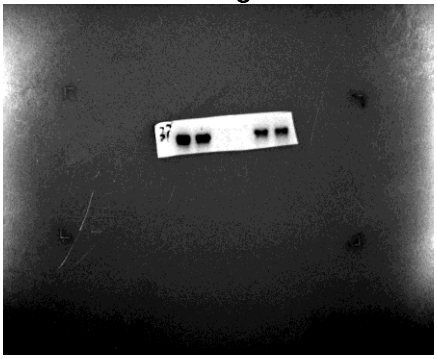

Figure 3N

Ub (IP)

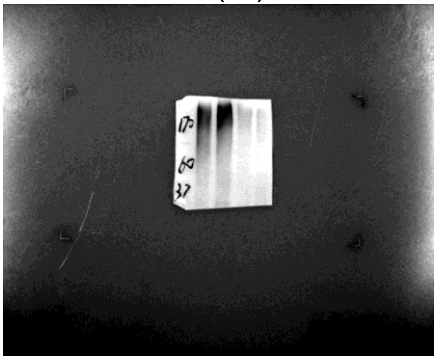

Flag (IP)

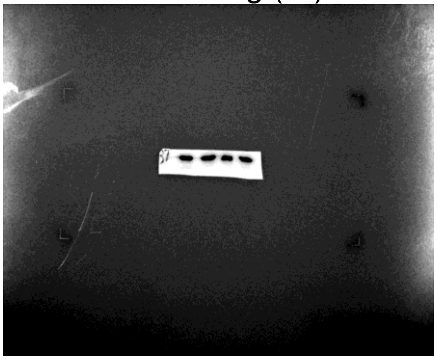

Flag (Input)

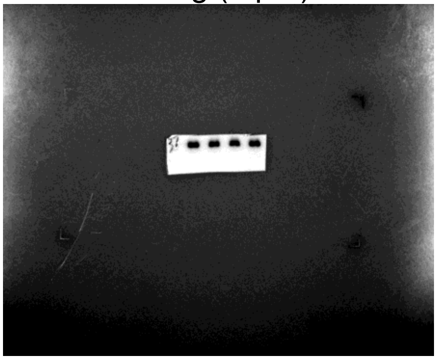

α-Tubulin

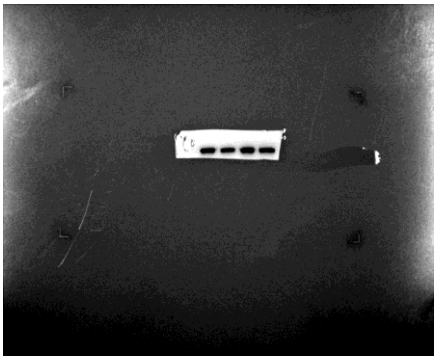

Figure 4B

Ub (BRCA2)

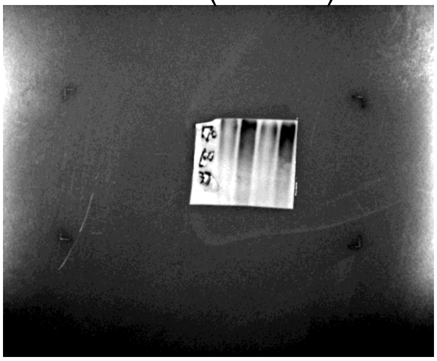

USP11 (IP)

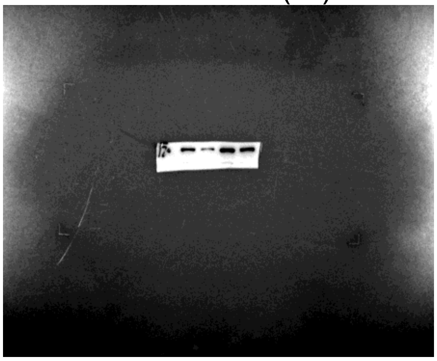

BRCA2 (IP)

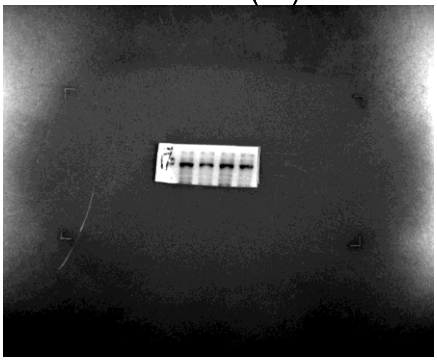

BRCA2 (Input)

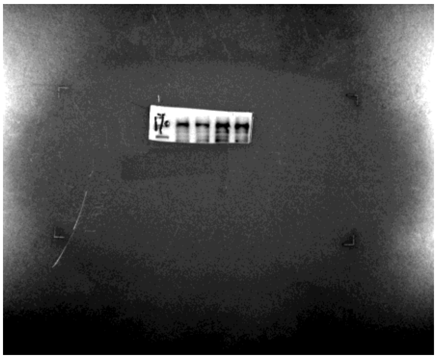

USP11 (Input)

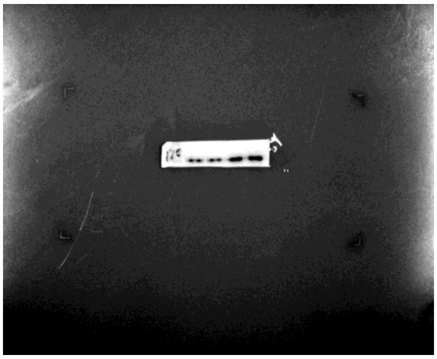

α-Tubulin

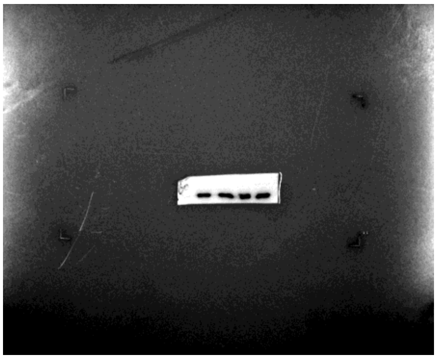

Figure 4C

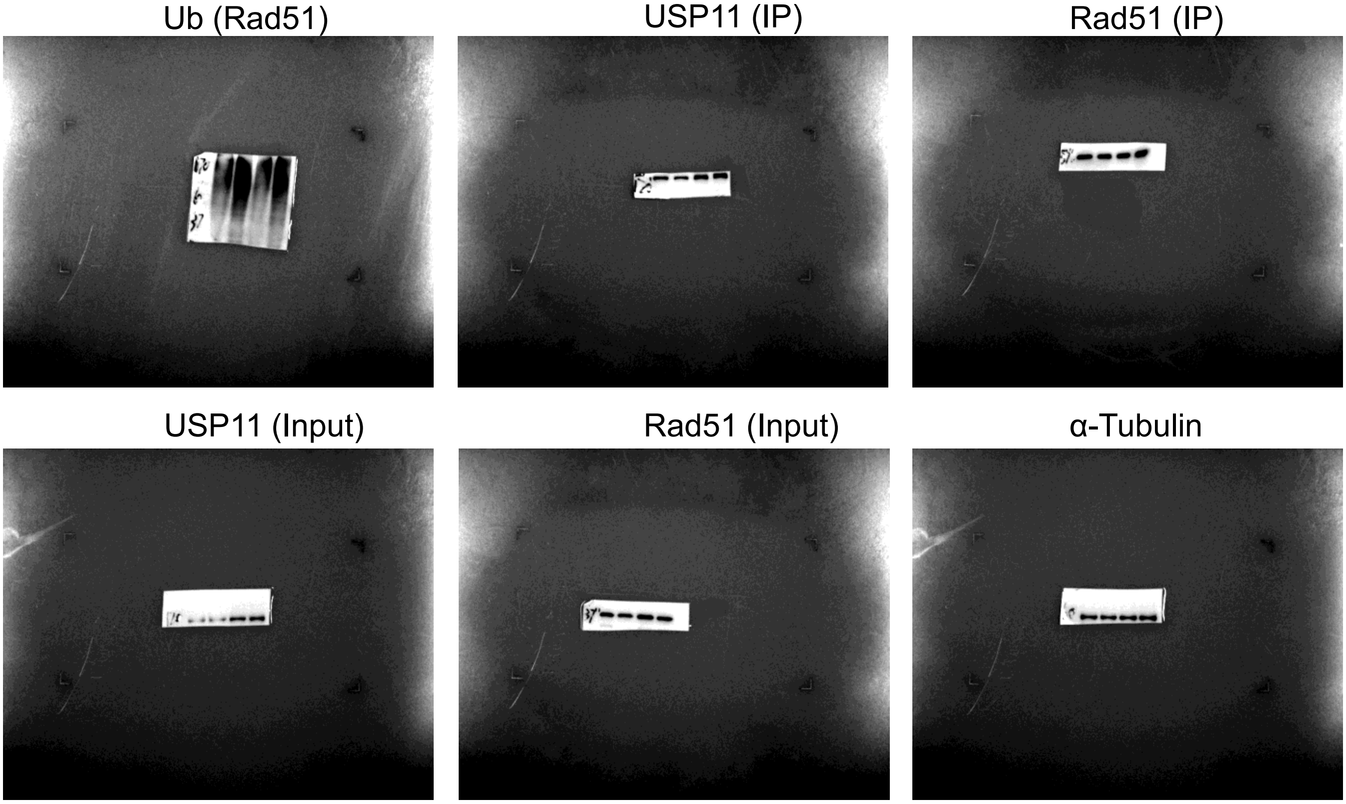

Figure 4D

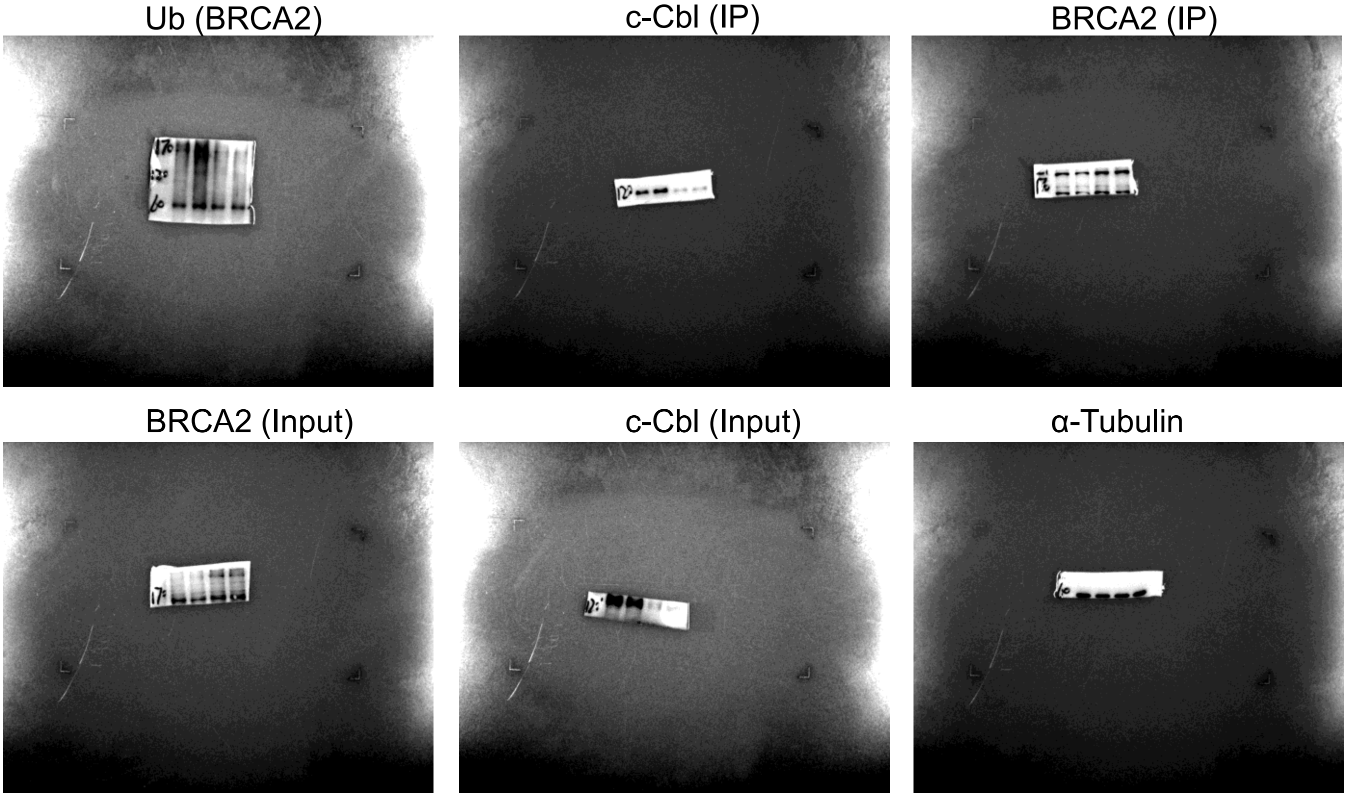

Figure 4E

Ub (Rad51)

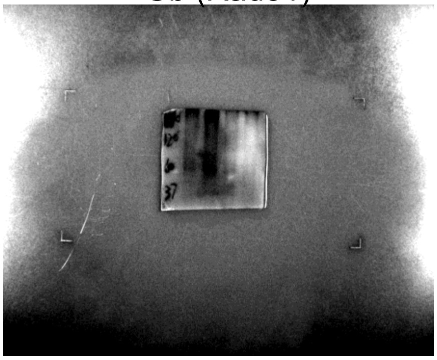

c-Cbl (IP)

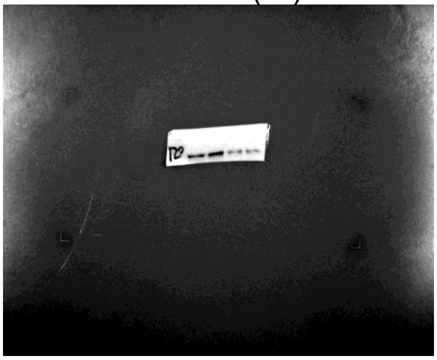

Rad51 (IP)

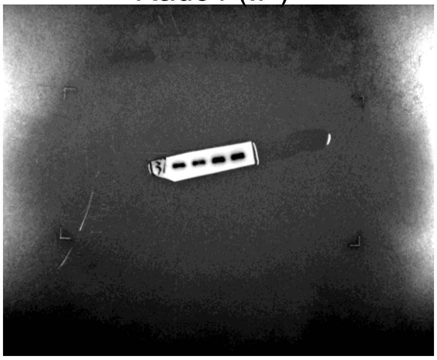

c-Cbl (Input)

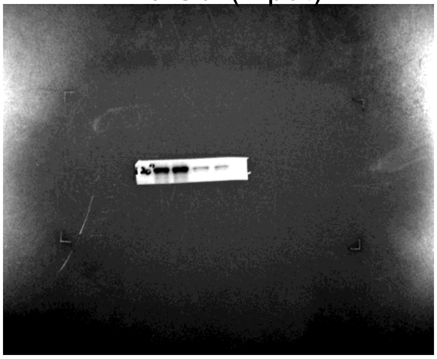

Rad51 (Input)

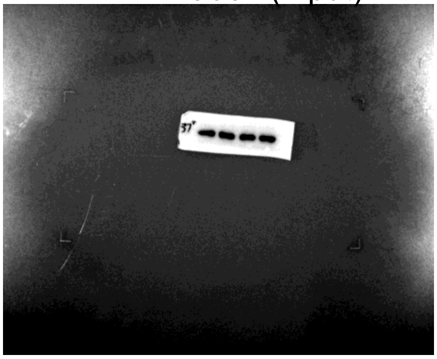

α-Tubulin

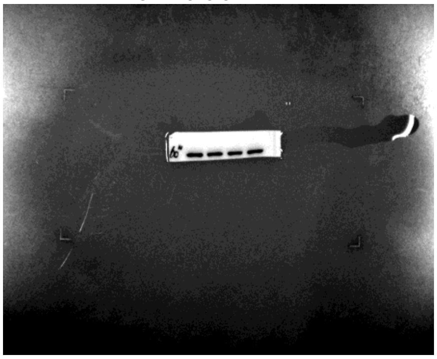

Figure 4F

BRCA2

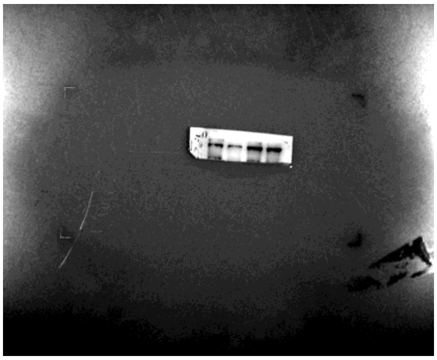

c-Cbl

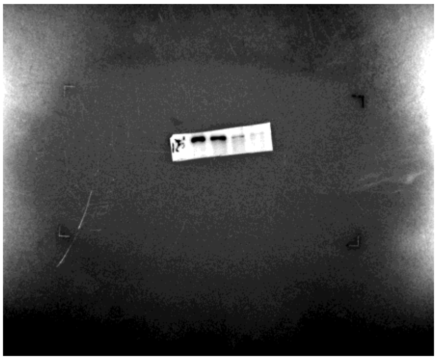

Rad51

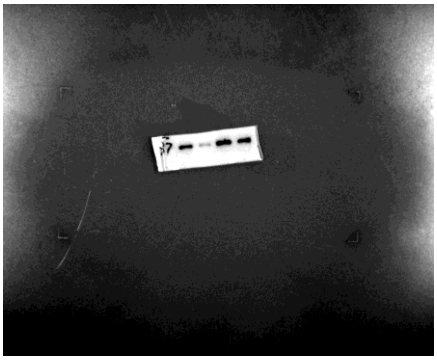

α-Tubulin

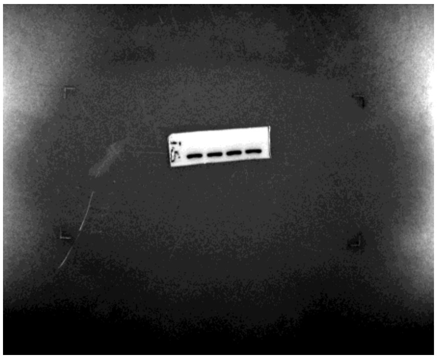

Figure 4G

BRCA2

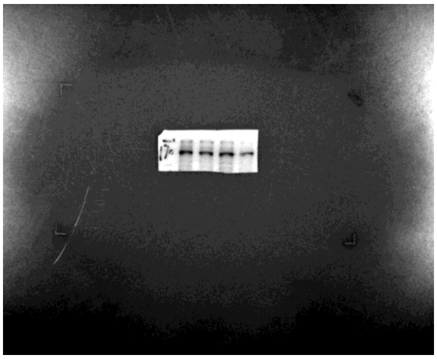

USP11

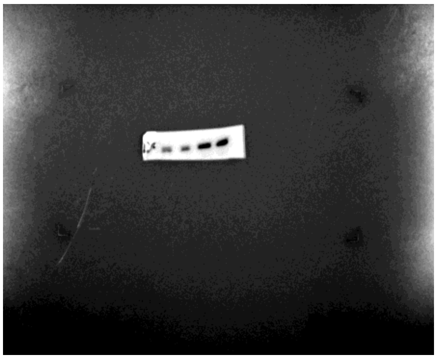

Rad51

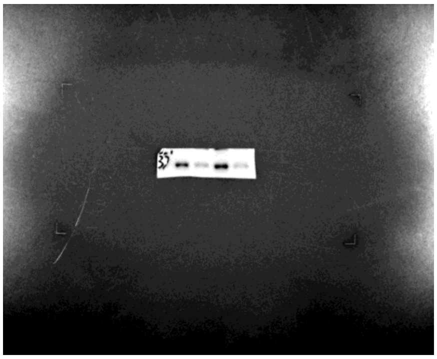

α-Tubulin

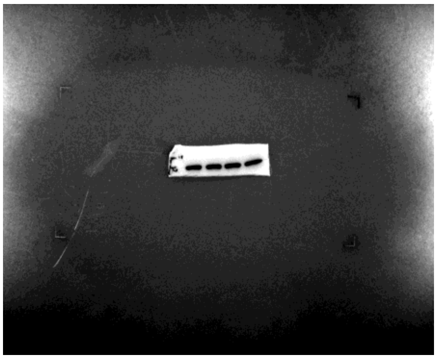

Figure 4J

Flag (Input)

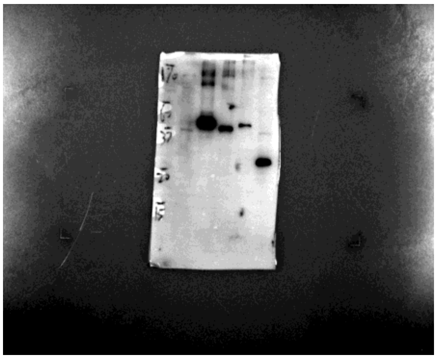

α-Tubulin

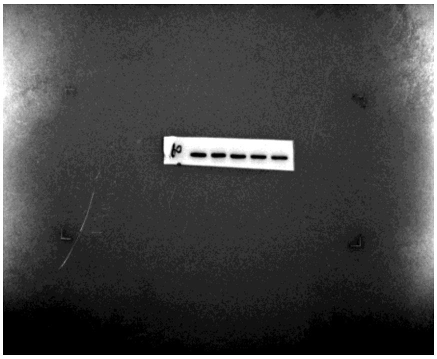

Flag (IP)

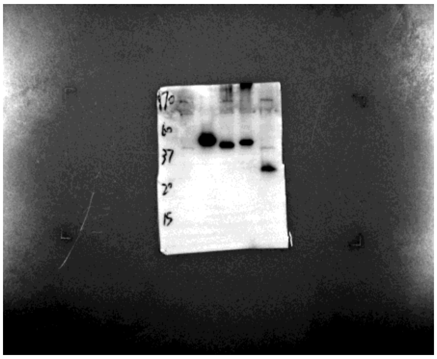

c-Cbl

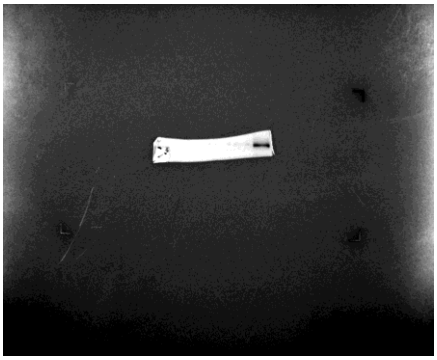

Figure 4J

c-Cbl (Input)

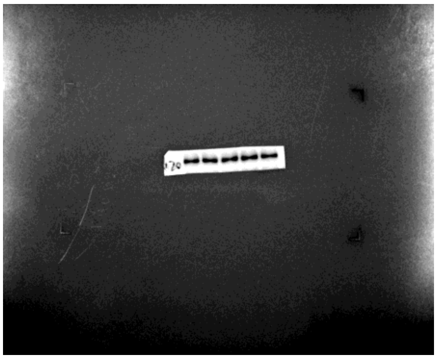

Flag (Input)

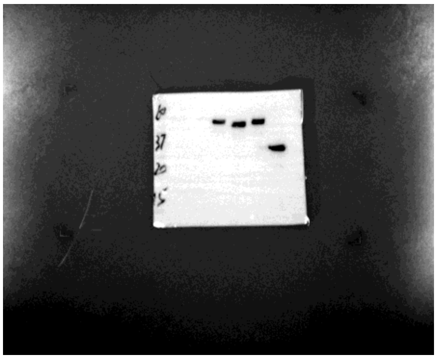

α-Tubulin

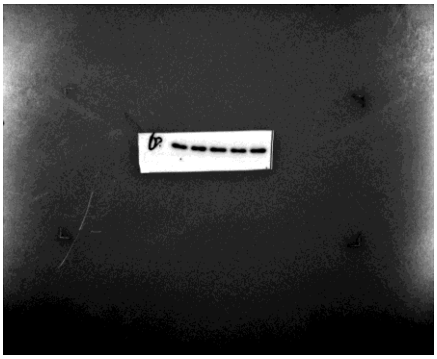

c-Cbl (IP)

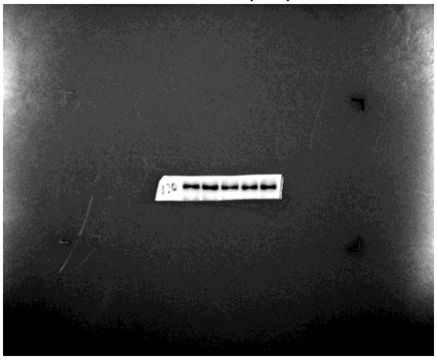

Flag (IP)

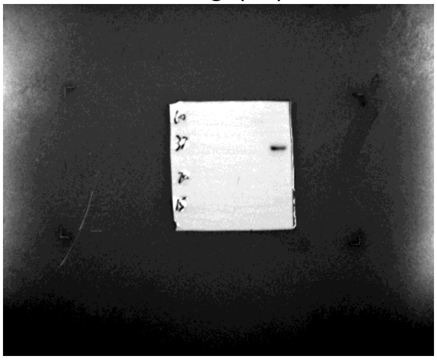

Figure 5D

EGFR

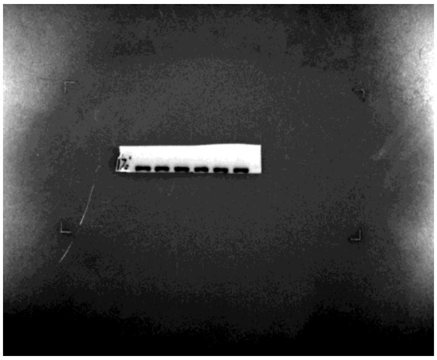

BRCA2

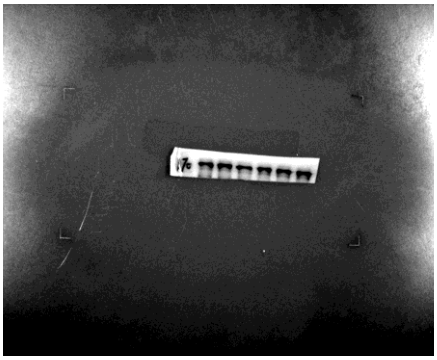

Rad51

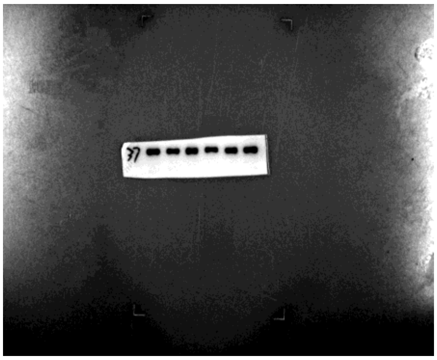

α-Tubulin

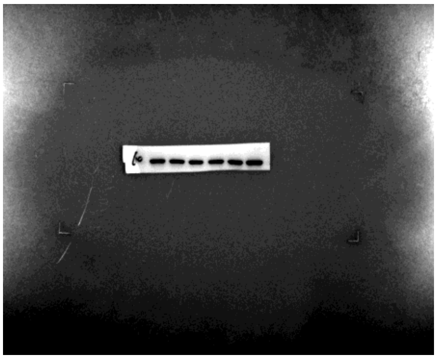

Figure 5E

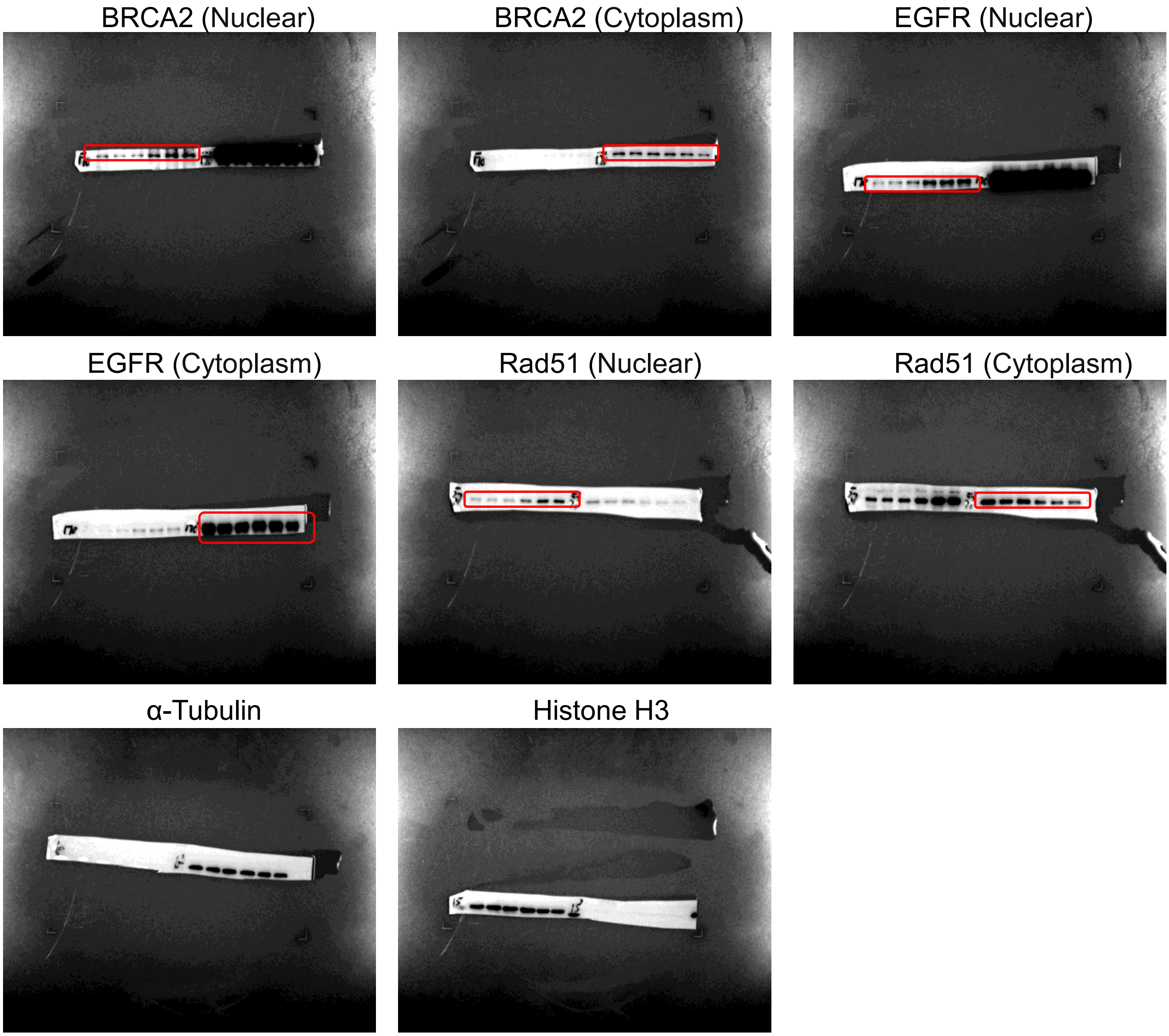

Figure 5F

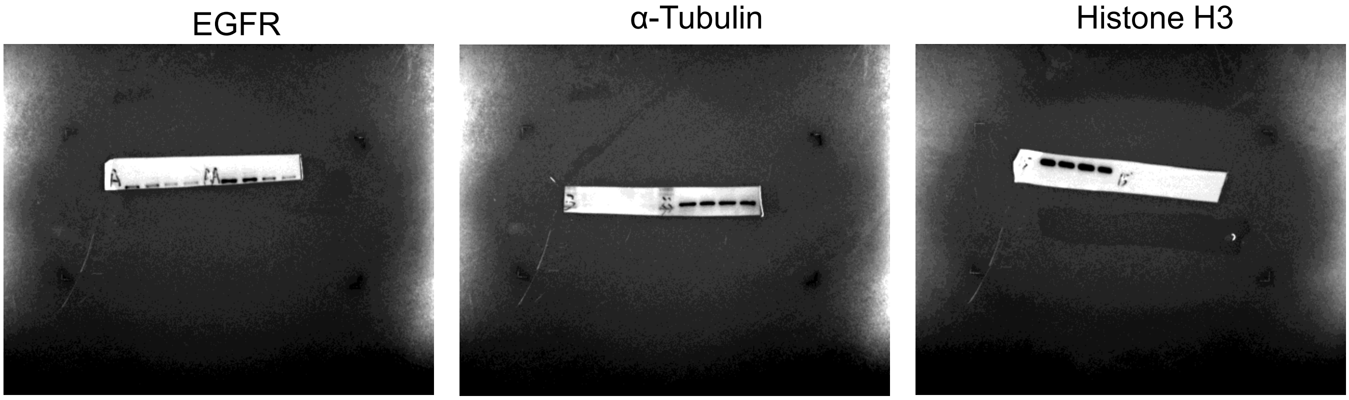

Figure 5G

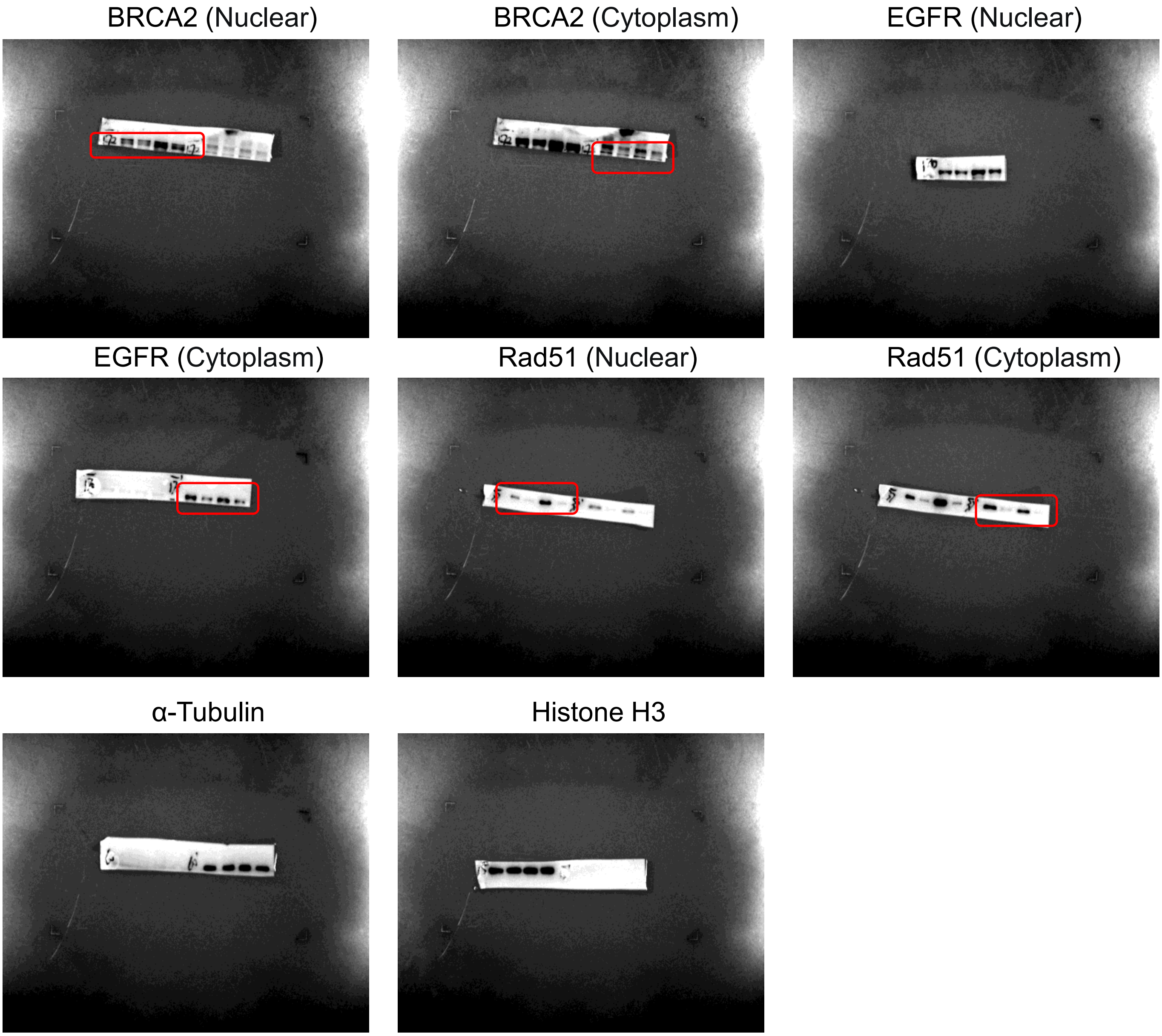
BRCA2 (Nuclear)
BRCA2 (Cytoplasm)
EGFR (Nuclear)
EGFR (Cytoplasm)
Rad51 (Nuclear)
Rad51 (Cytoplasm)
α-Tubulin
Histone H3

Figure 5K

γH2AX

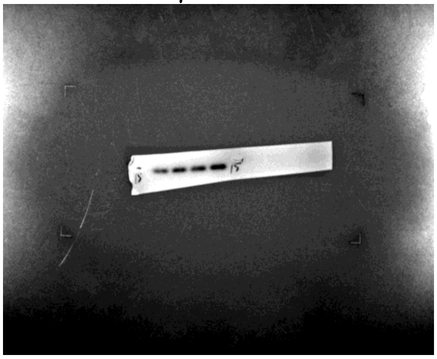

Histone H3

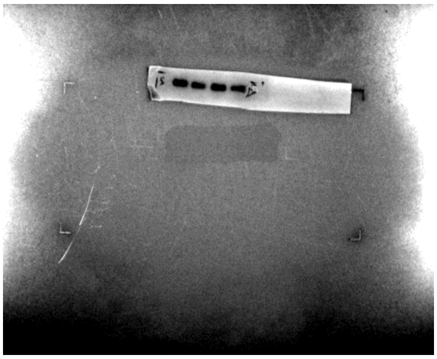

Figure 6A

ATM (Nuclear)

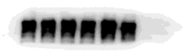

ATM (Cytoplasm)

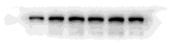

α-Tubulin

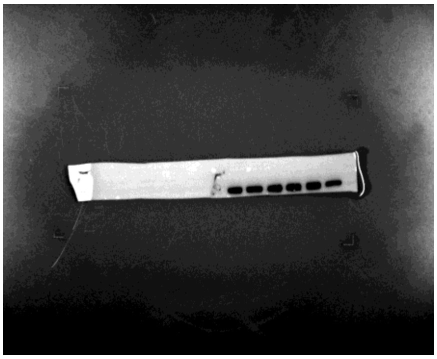

Histone H3

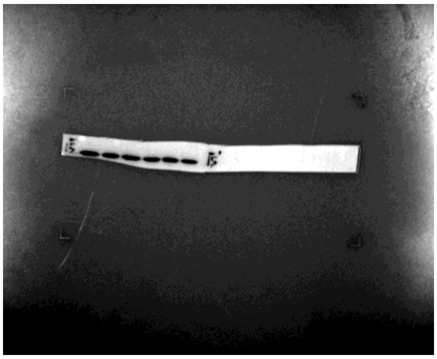

Figure 6B

ATM

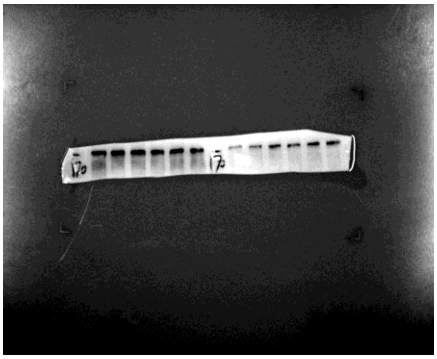

α-Tubulin

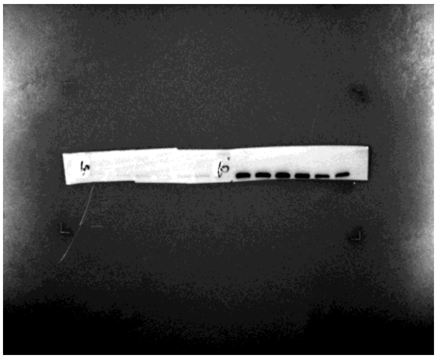

Histone H3

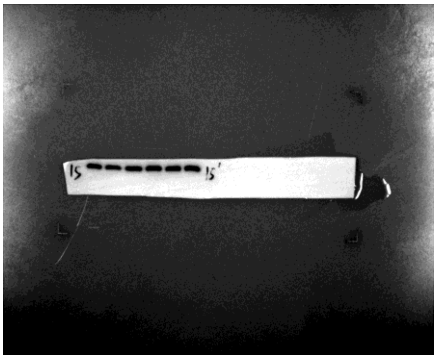

Figure 6C

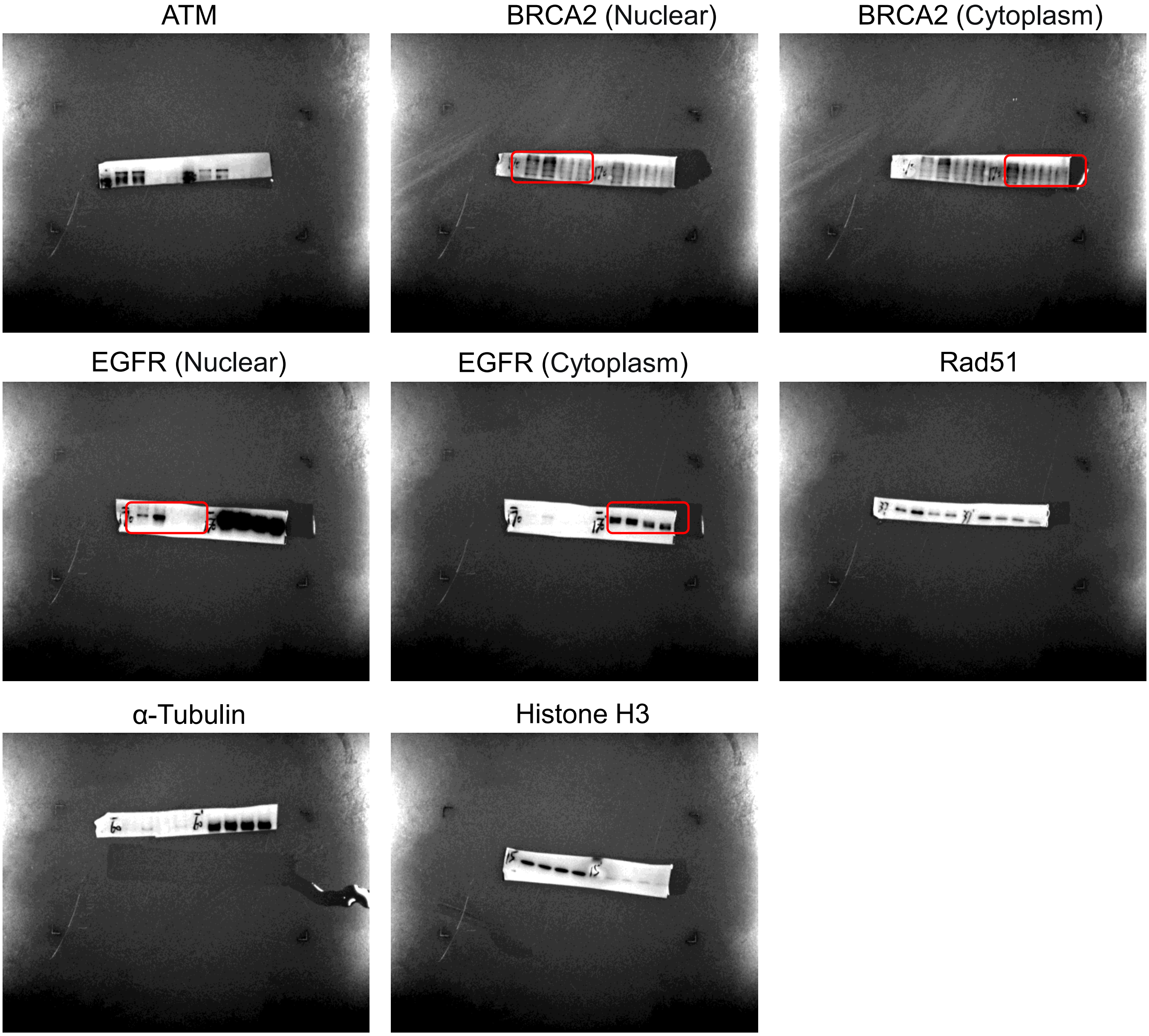
ATM
BRCA2 (Nuclear)
BRCA2 (Cytoplasm)
EGFR (Nuclear)
EGFR (Cytoplasm)
Rad51
α-Tubulin
Histone H3

Figure 6D

ATM

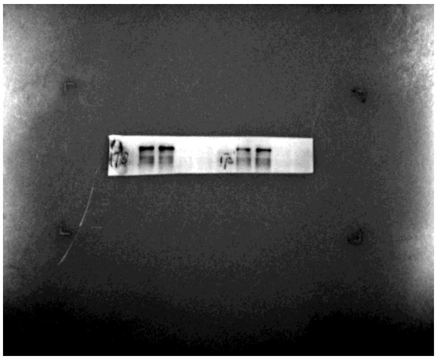

BRCA2 (Nuclear)

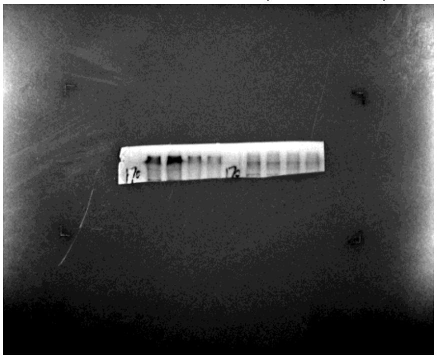

BRCA2 (Cytoplasm)

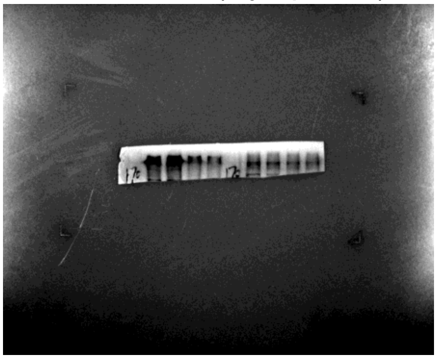

EGFR (Nuclear)

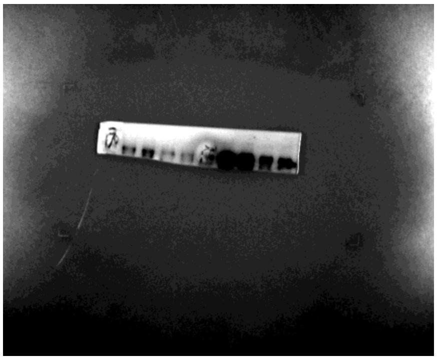

EGFR (Cytoplasm)

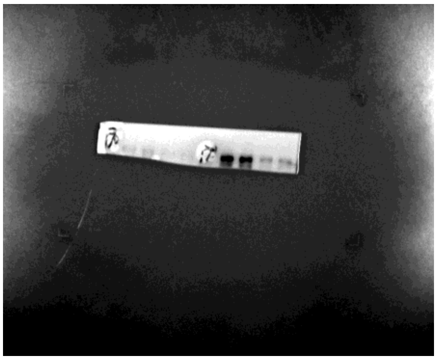

Rad51

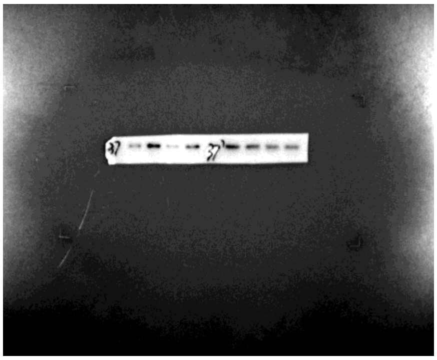

α-Tubulin

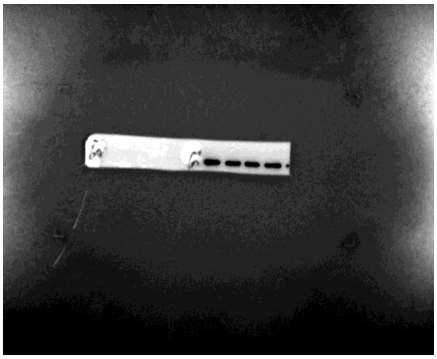

Histone H3

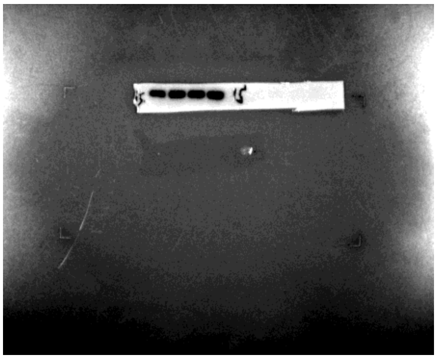

Figure 6E

ATM

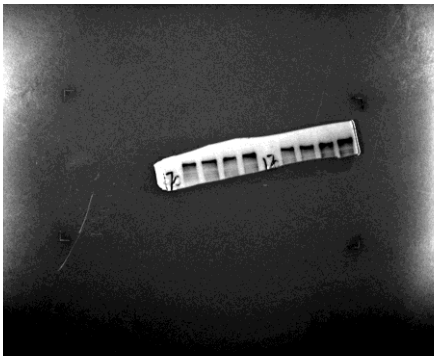

α-Tubulin

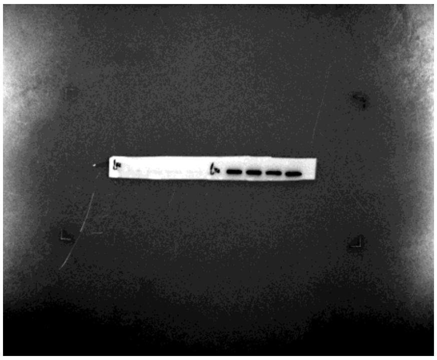

Histone H3

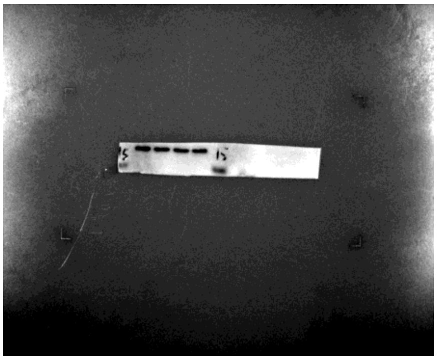

Figure 6F

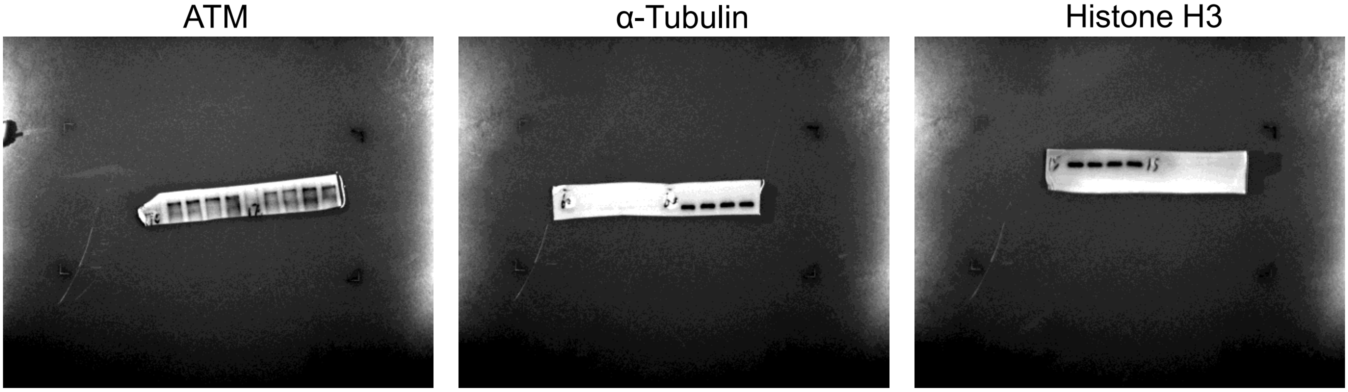

Figure 7D

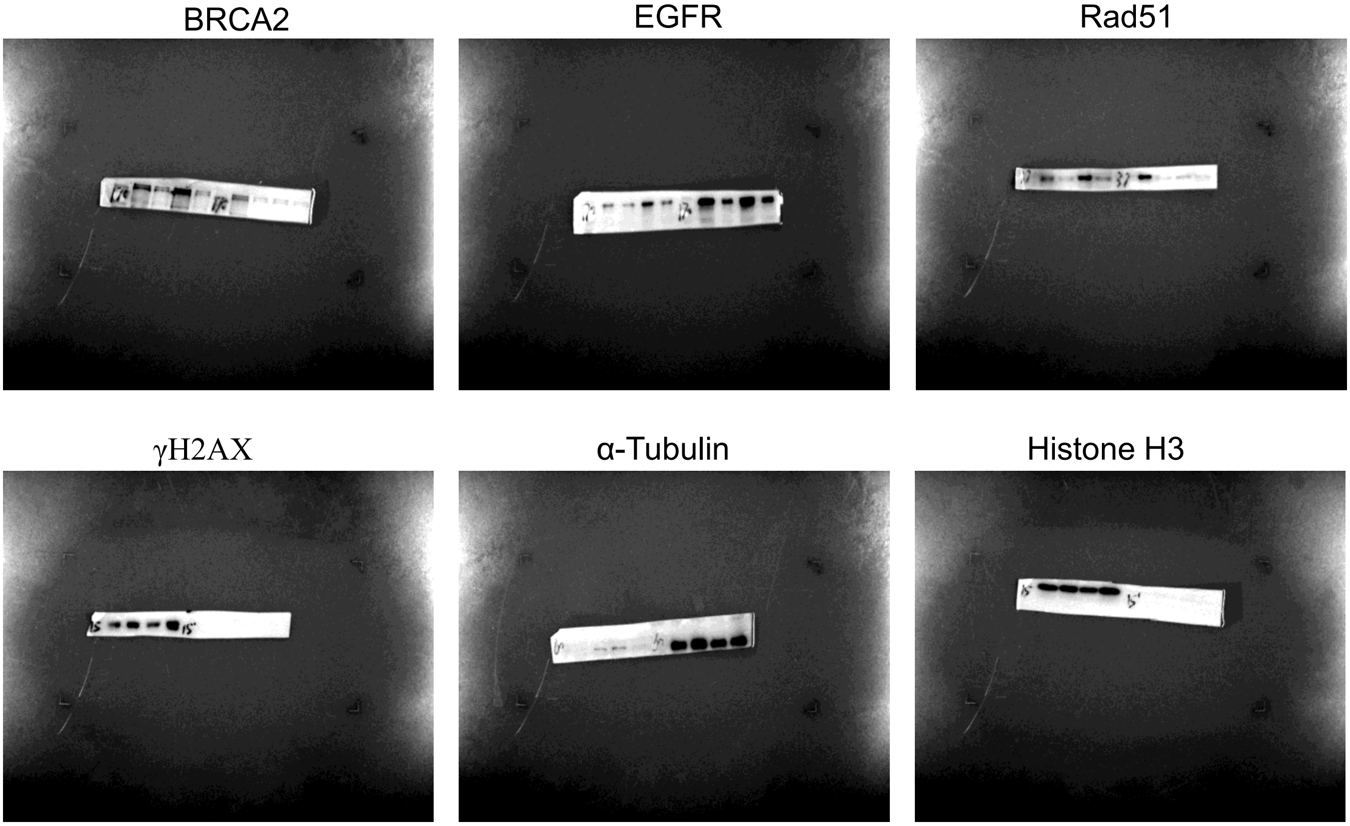

Supplementary Figure 1B

EGFR (Case 1)

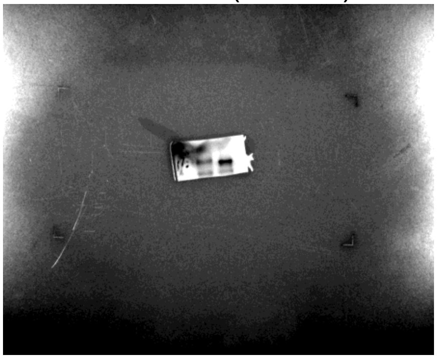

α-Tubulin (Case 1)

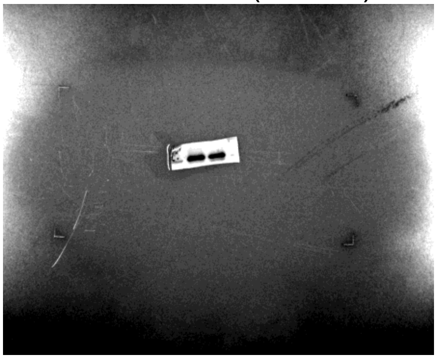

EGFR (Case 2)

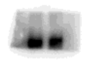

α-Tubulin (Case 2)

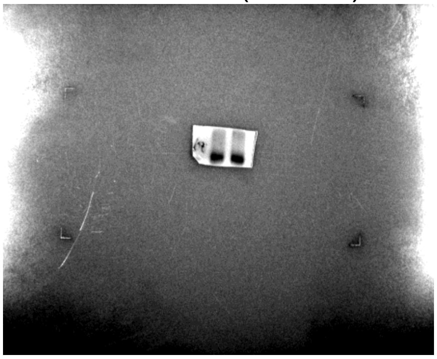

EGFR (Case 3)

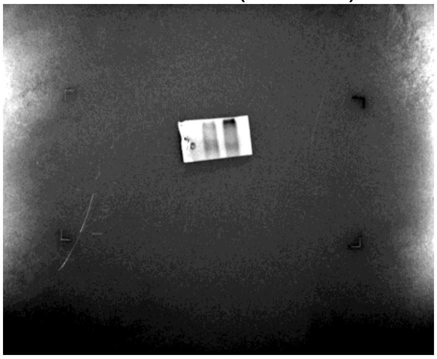

α-Tubulin (Case 3)

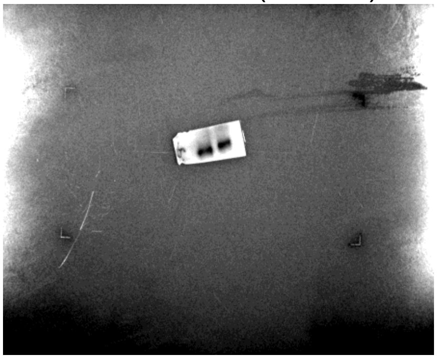

EGFR (Case 4)

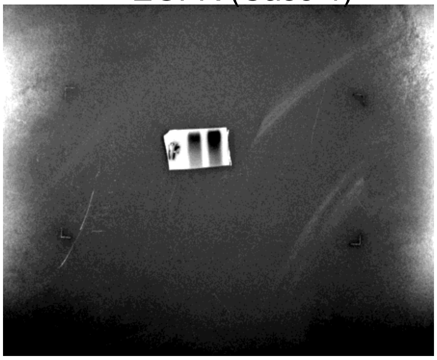

α-Tubulin (Case 4)

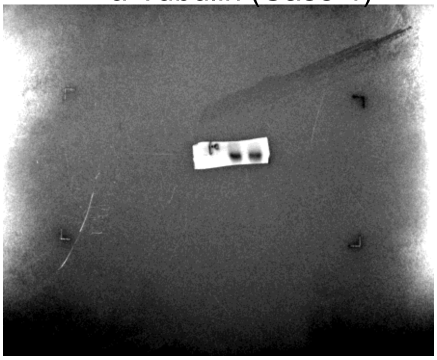

Supplementary Figure 1D

EGFR

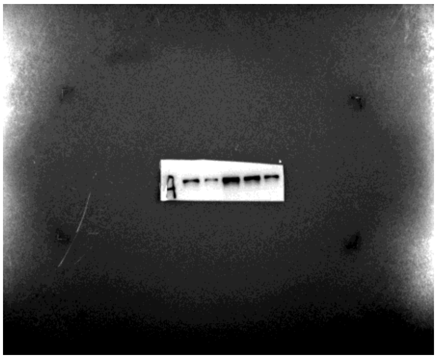

α-Tubulin

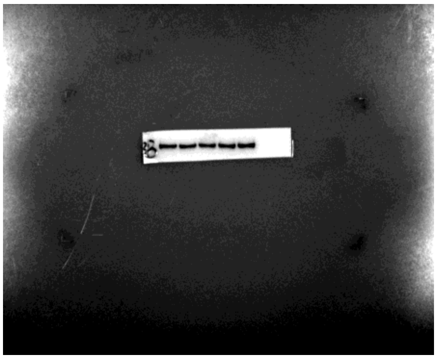

Supplementary Figure 1F

EGFR (SKOV3)

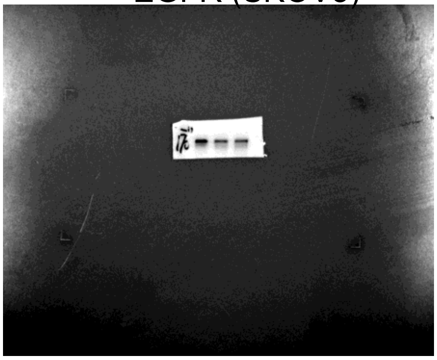

α-Tubulin (SKOV3)

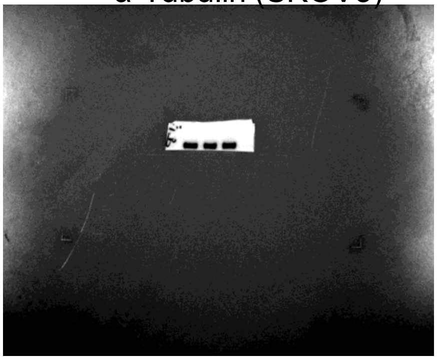

EGFR (OVCAR3)

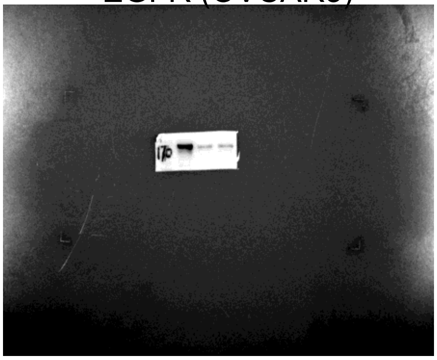

α-Tubulin (OVCAR3)

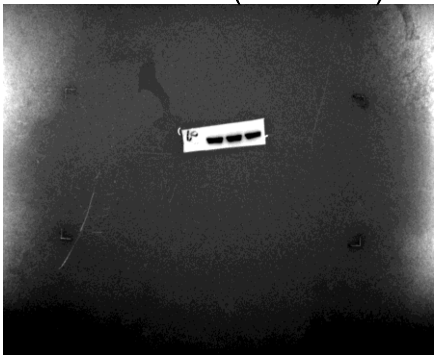

A2780

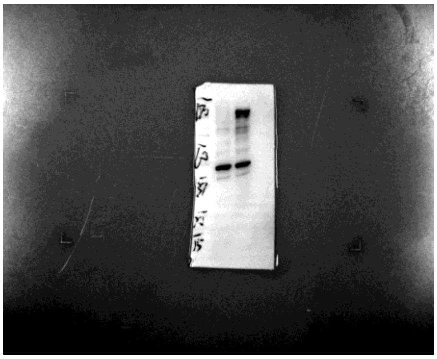

Supplementary Figure 2E

EGFR

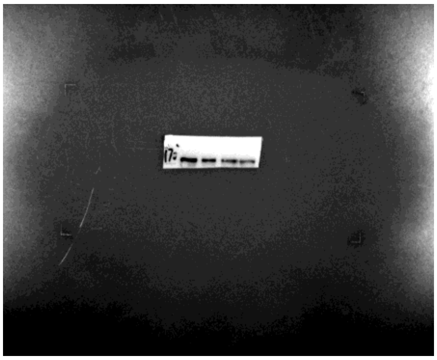

p-EGFR

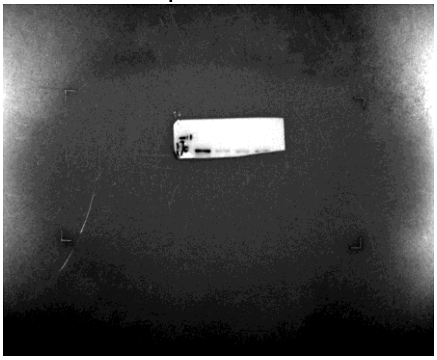

α-Tubulin

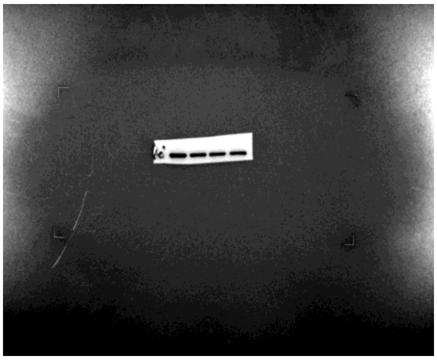

Supplementary Figure 2G

EGFR

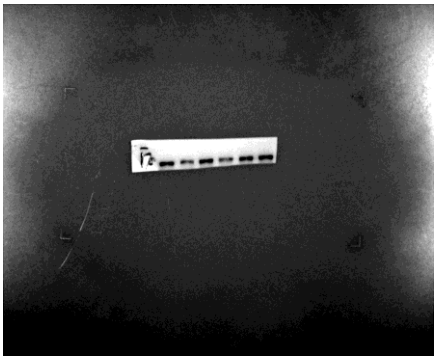

α-Tubulin

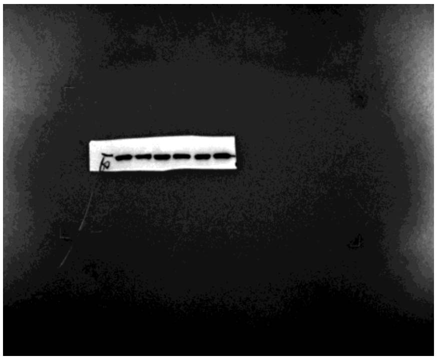

Supplementary Figure 2H

Ub

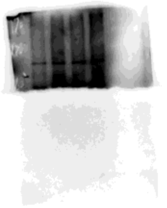

EGFR (IP)

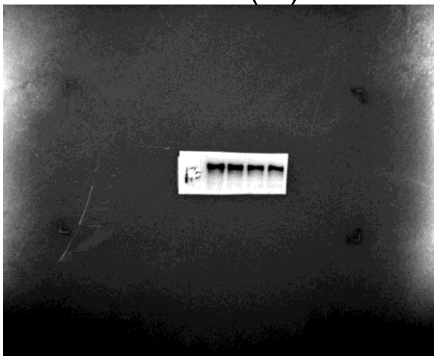

EGFR (Input)

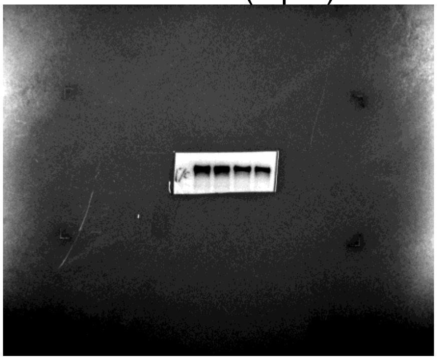

α-Tubulin

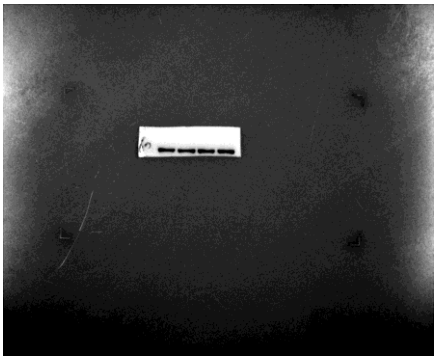

Supplementary Figure 2J

HER2

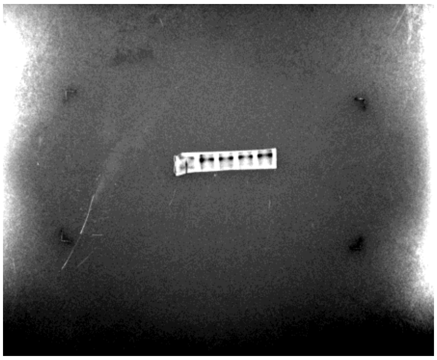

IGF1R

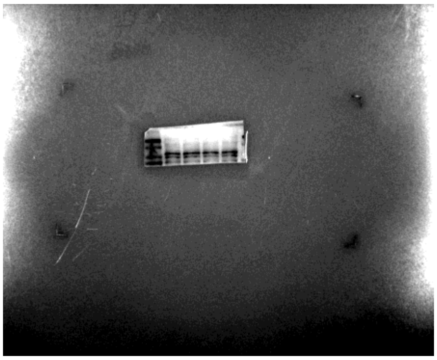

α-Tubulin

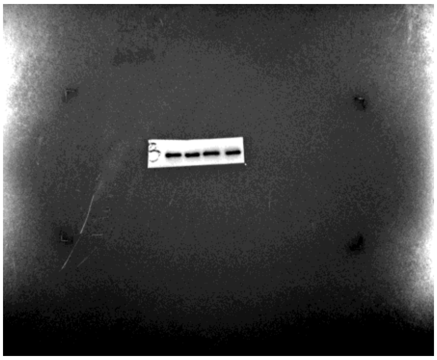

Supplementary Figure 2K

EGFR

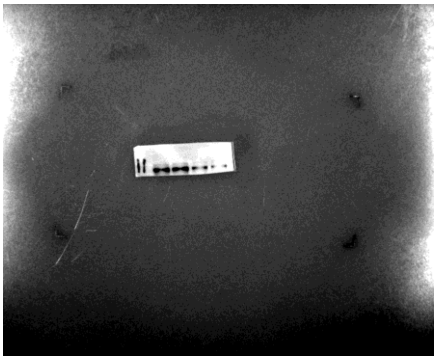

p-EGFR

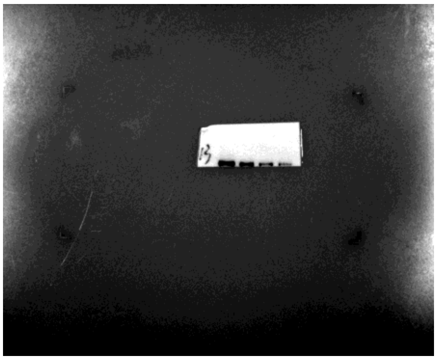

α-Tubulin

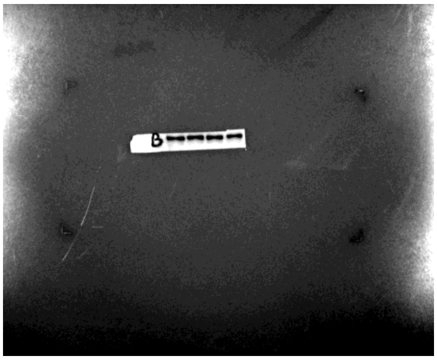

Supplementary Figure 3B

BRCA2

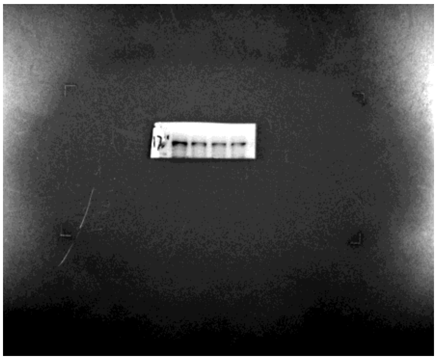

Rad51

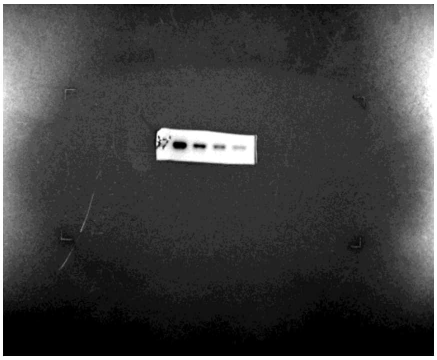

α-Tubulin

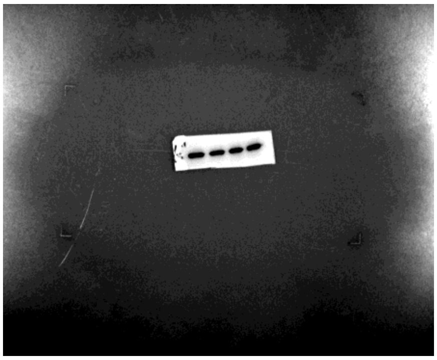

Supplementary Figure 3C

BRCA2

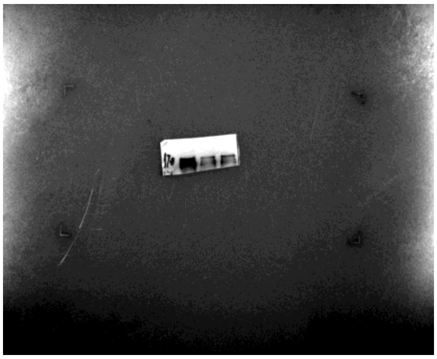

EGFR

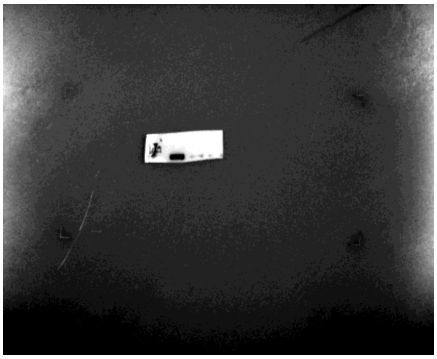

Rad51

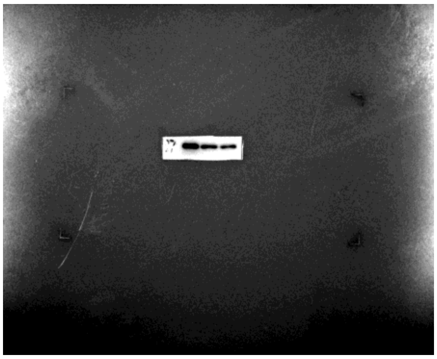

α-Tubulin

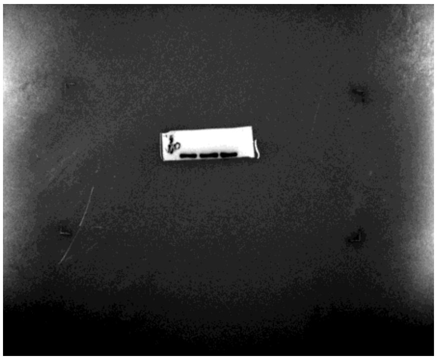

Supplementary Figure 3D

BRCA2

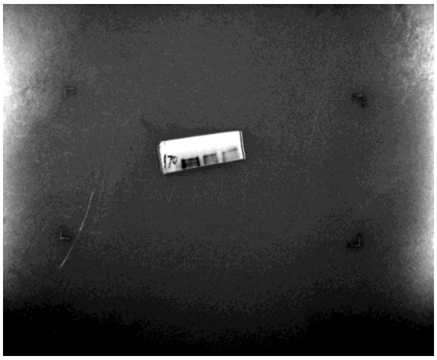

EGFR

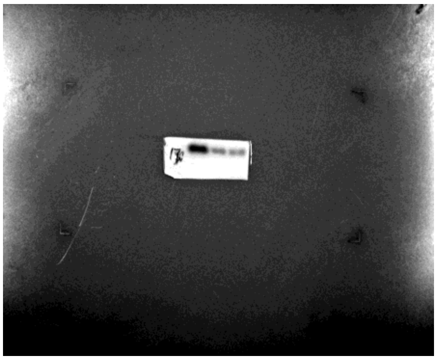

Rad51

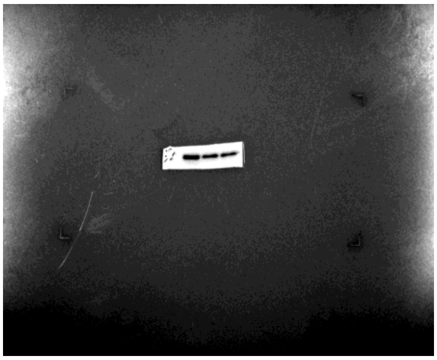

α-Tubulin

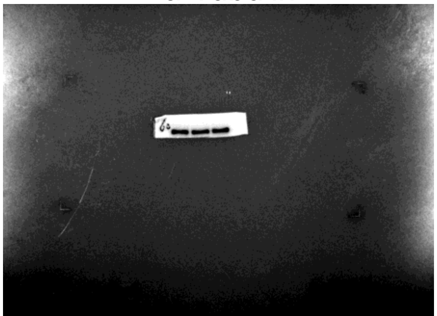

Supplementary Figure 3E

BRCA2

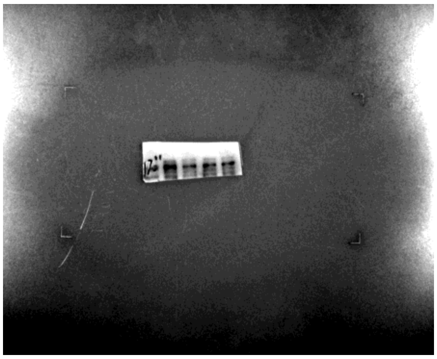

EGFR

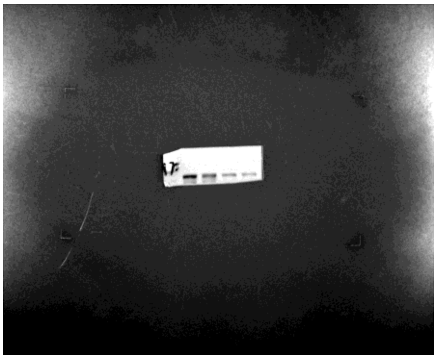

Rad51

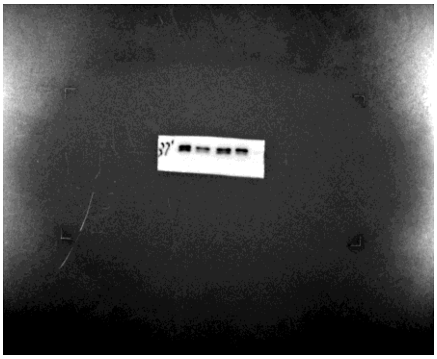

α-Tubulin

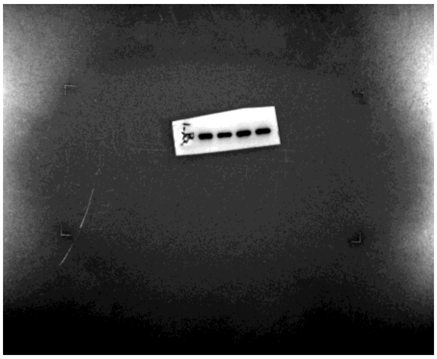

Supplementary Figure 3F

BRCA2

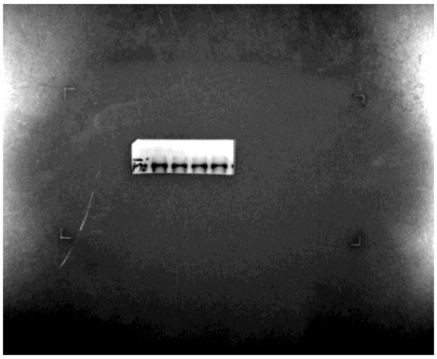

p-EGFR

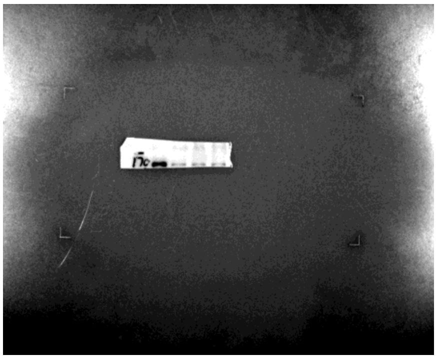

Rad51

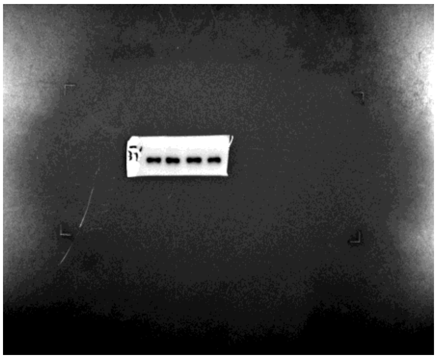

α-Tubulin

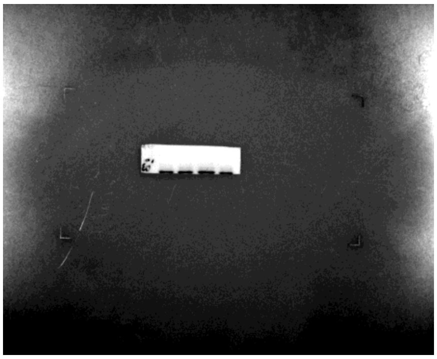

Supplementary Figure 3G

EGFR

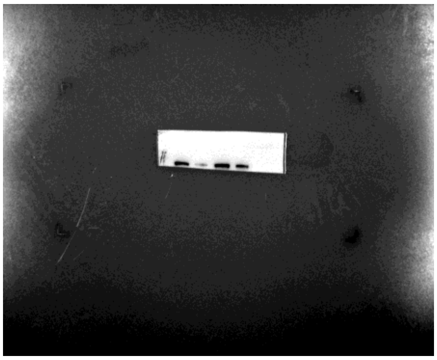

BRCA2

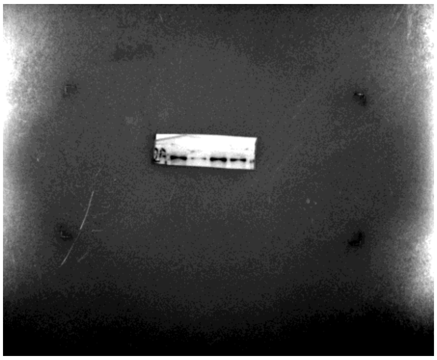

Rad51

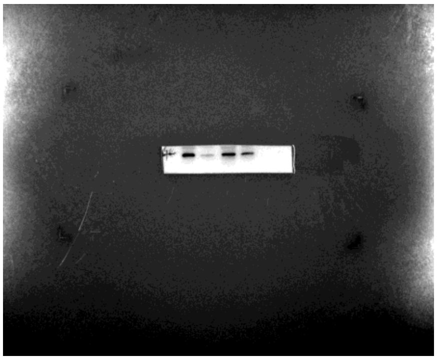

α-Tubulin

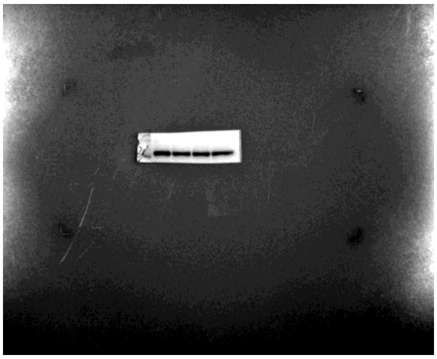

Supplementary Figure 3I

BRCA2

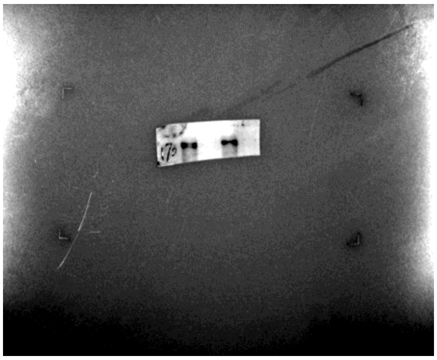

Rad51

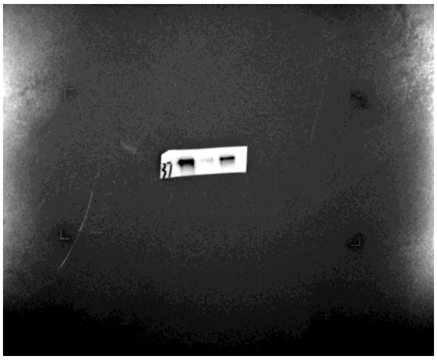

EGFR

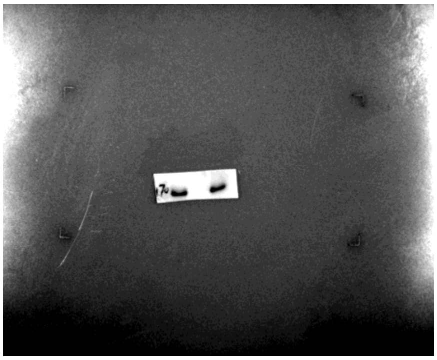

Supplementary Figure 3J

Flag (Input)

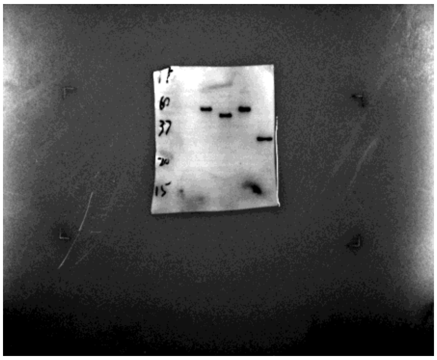

α-Tubulin

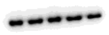

Flag (IP)

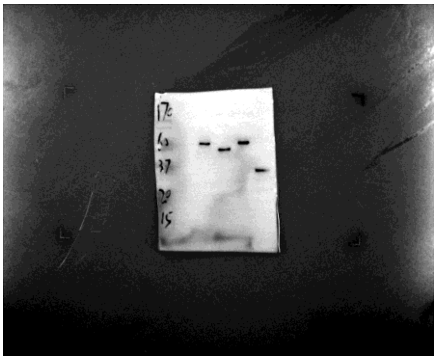

EGFR

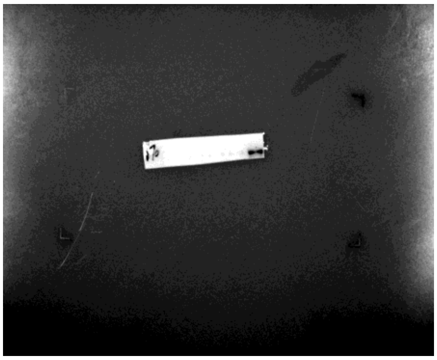

Supplementary Figure 3K

EGFR (Input)

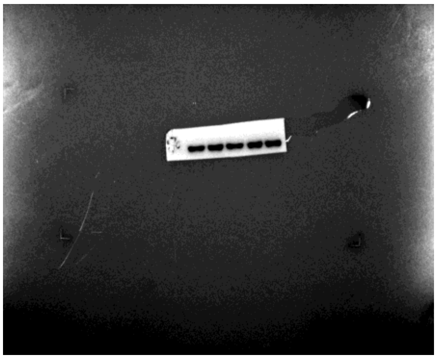

Flag (Input)

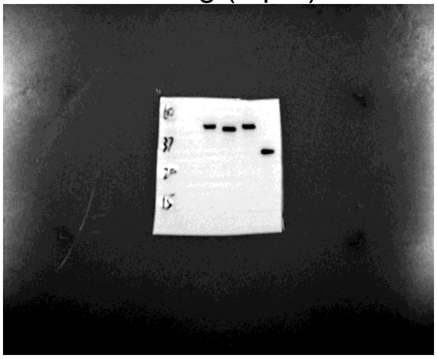

α-Tubulin

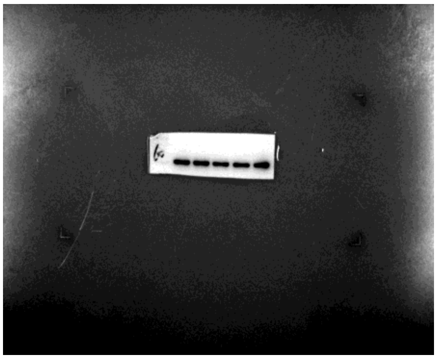

EGFR (IP)

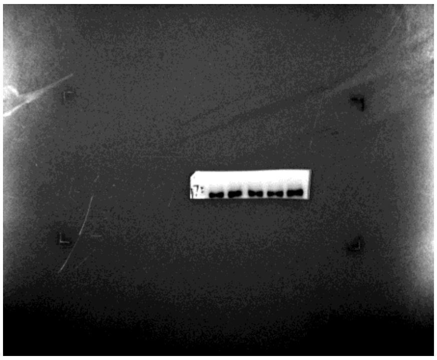

Flag (IP)

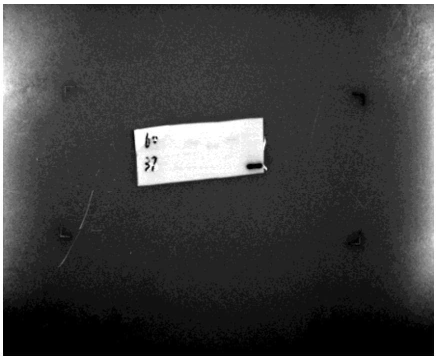

Supplementary Figure 3L

EGFR

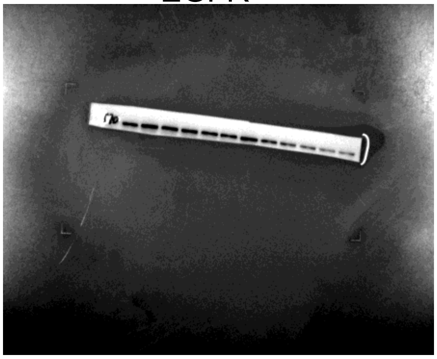

BRCA2

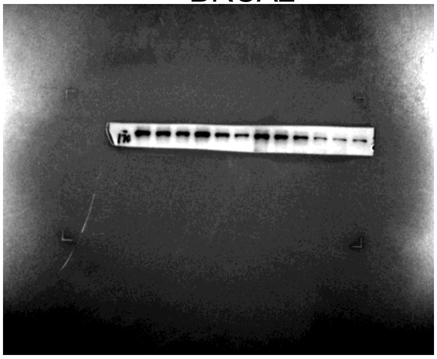

Rad51

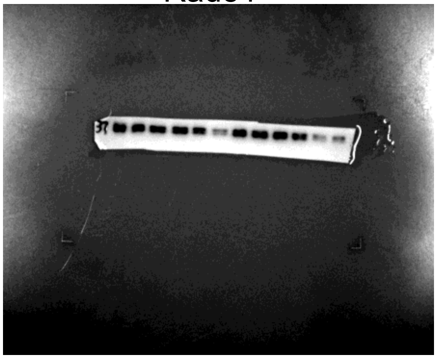

γH2AX

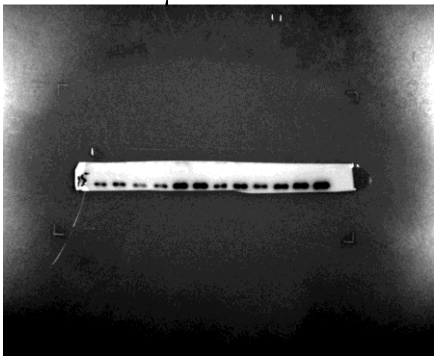

α-Tubulin

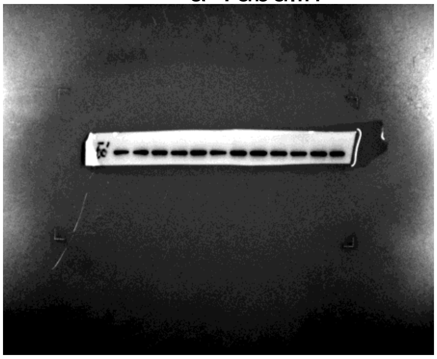

Supplementary Figure 3M

EGFR

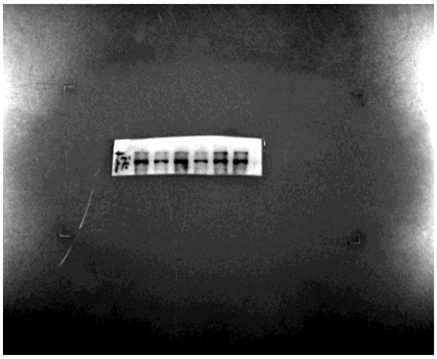

Rad51

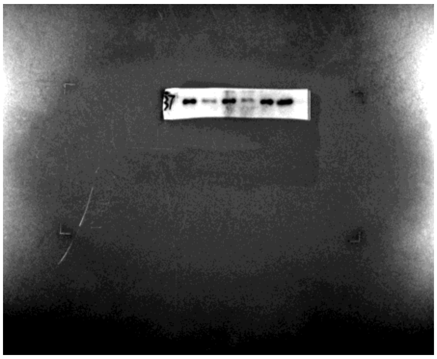

α-Tubulin

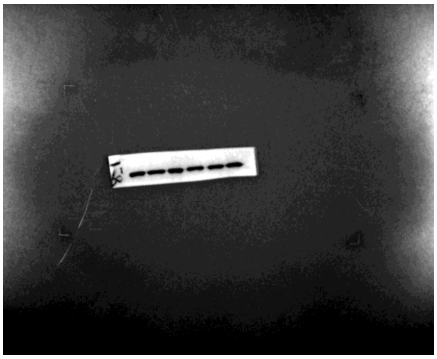

Supplementary Figure 3N

EGFR

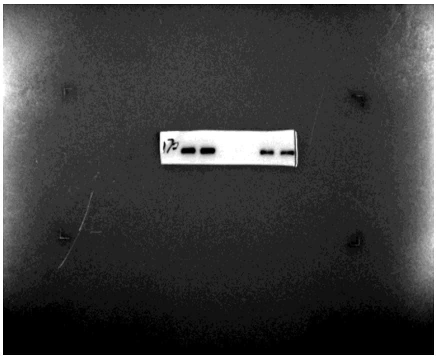

Flag

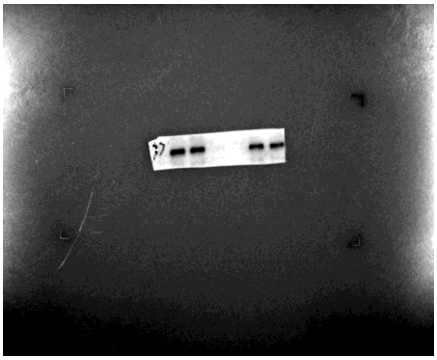

Supplementary Figure 3O

Ub (IP)

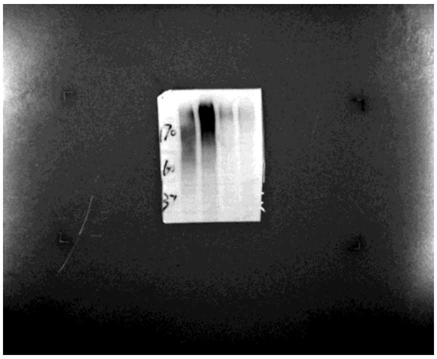

Flag (IP)

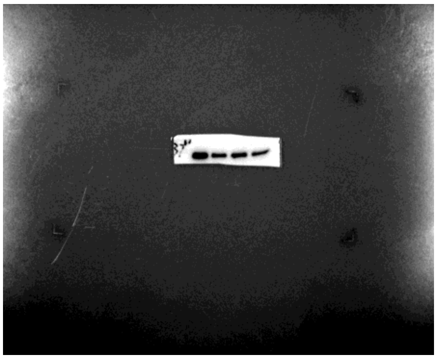

Flag (Input)

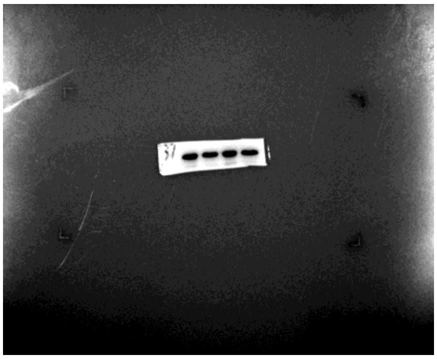

α-Tubulin

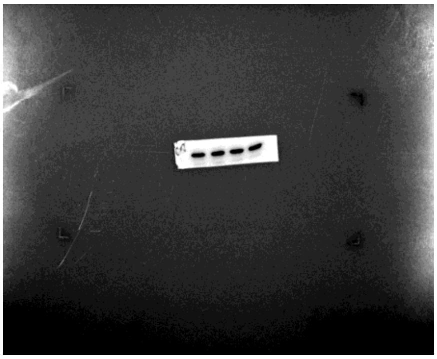

Supplementary Figure 4A

Ub (BRCA2)

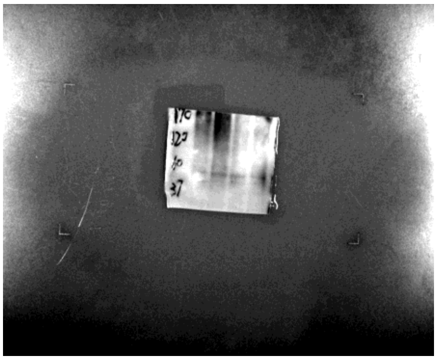

c-Cbl (IP)

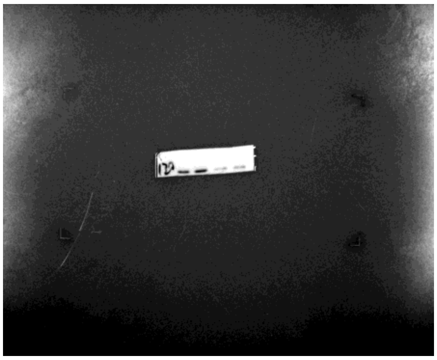

BRCA2 (IP)

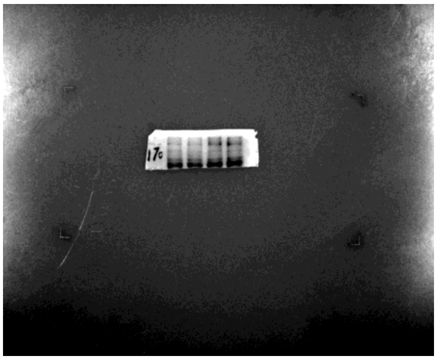

BRCA2 (Input)

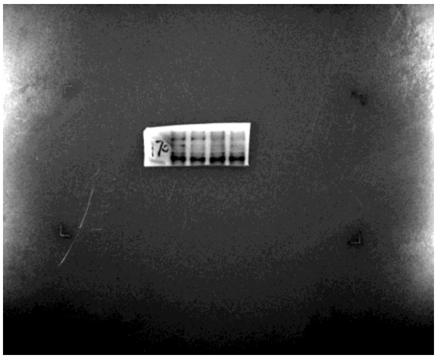

c-Cbl (Input)

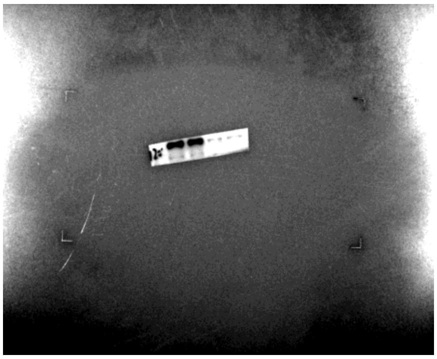

α-Tubulin

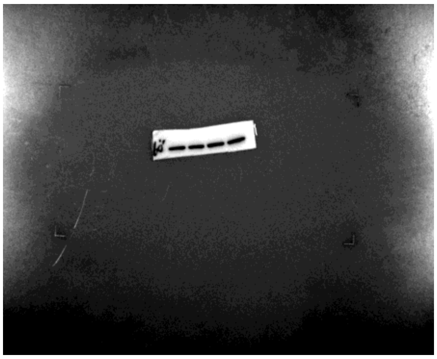

Supplementary Figure 4B

Ub (Rad51)

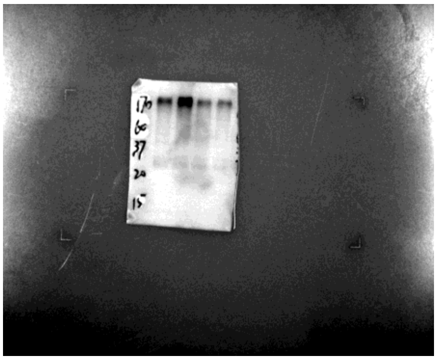

c-Cbl (IP)

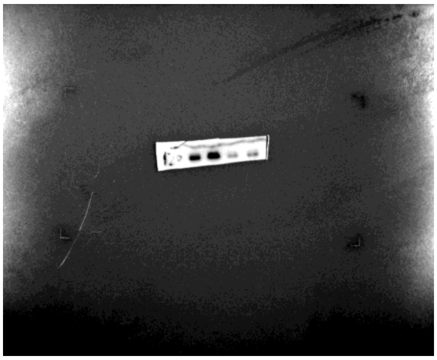

Rad51 (IP)

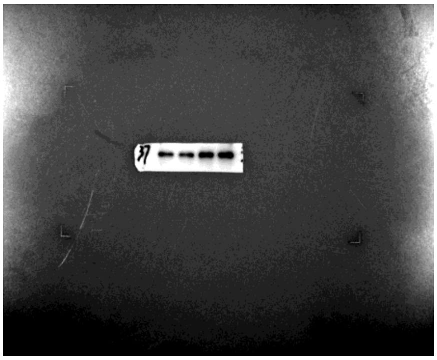

c-Cbl (Input)

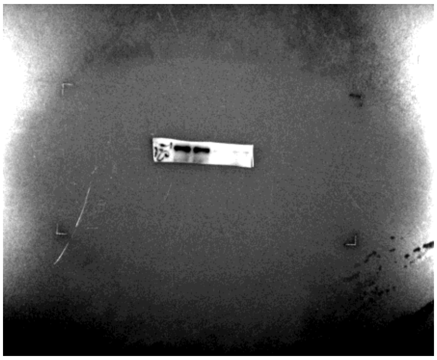

Rad51 (Input)

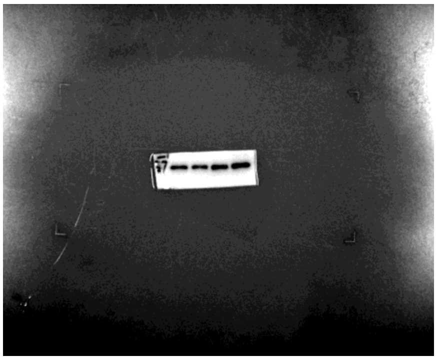

α-Tubulin

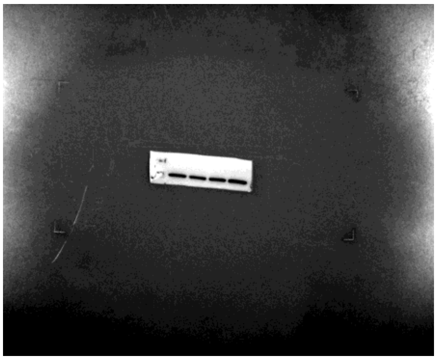

Supplementary Figure 4C

c-Cbl

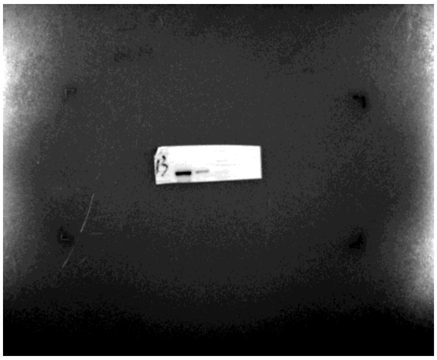

BRCA2

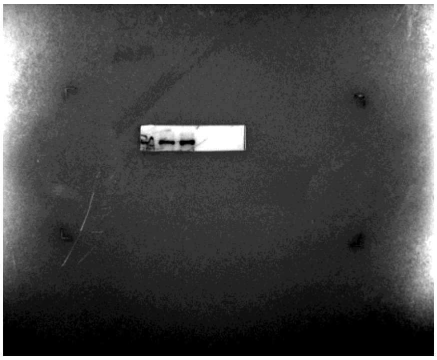

Rad51

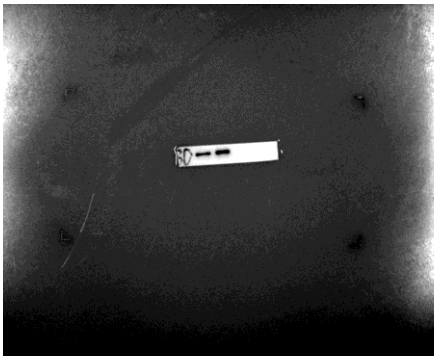

α-Tubulin

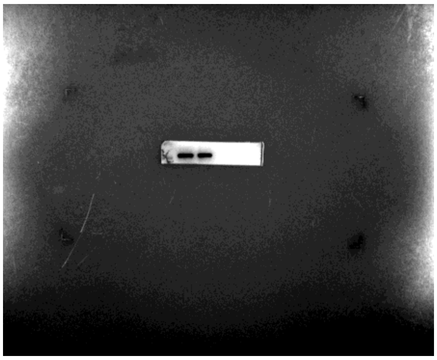

Supplementary Figure 4D

BRCA2

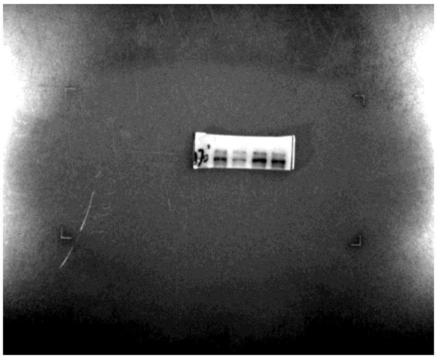

c-Cbl

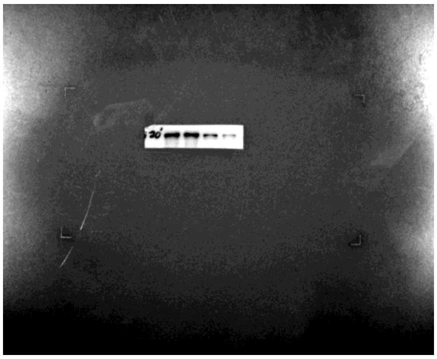

Rad51

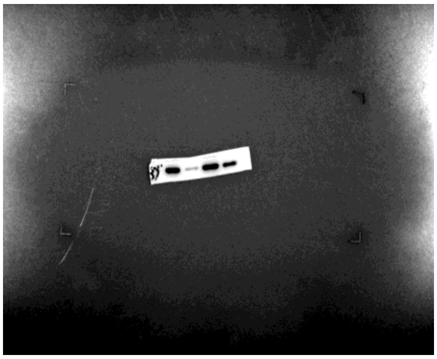

α-Tubulin

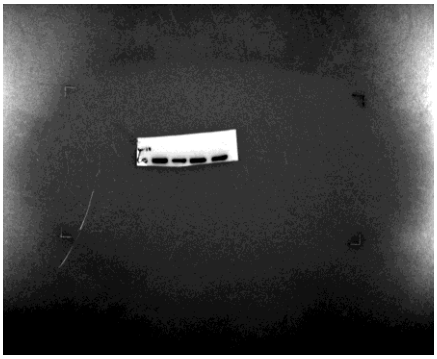

Supplementary Figure 4E

BRCA2

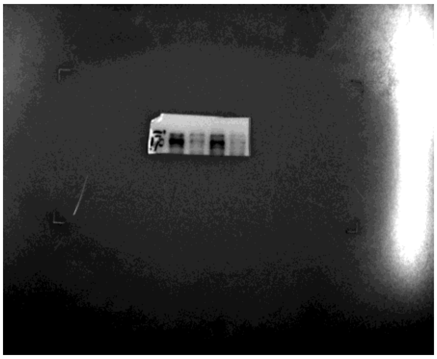

USP11

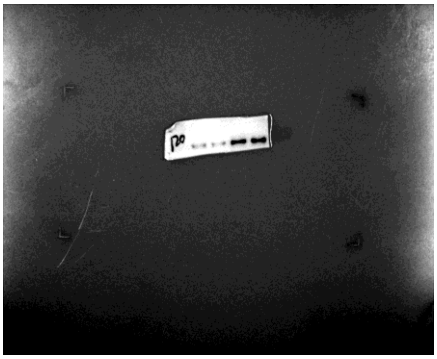

Rad51

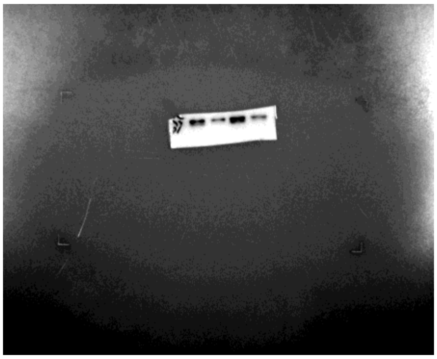

α-Tubulin

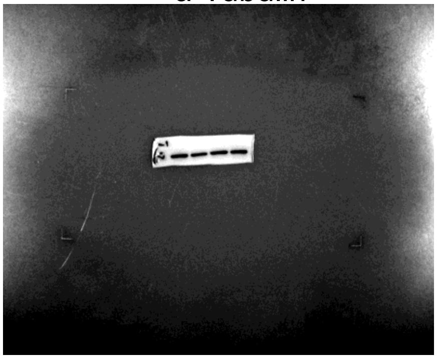

Supplementary Figure 4F

Flag (Input)

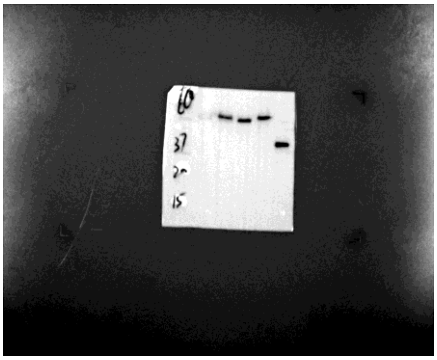

α-Tubulin

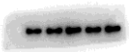

Flag (IP)

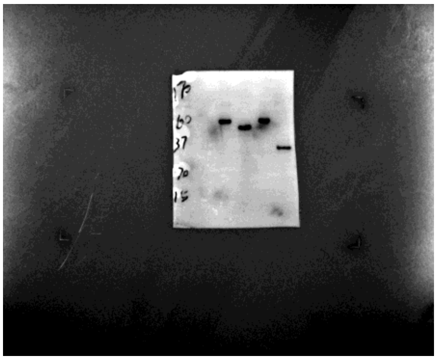

c-Cbl

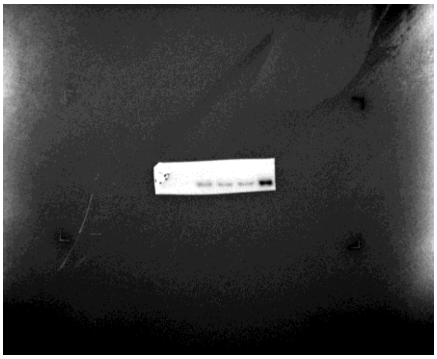

Supplementary Figure 4G

c-Cbl (Input)

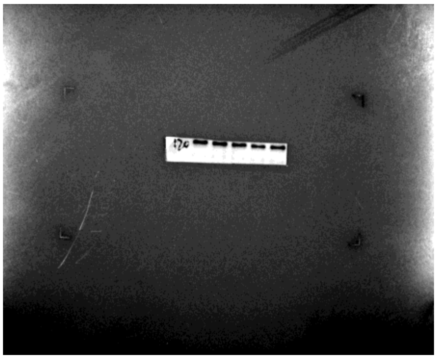

Flag (Input)

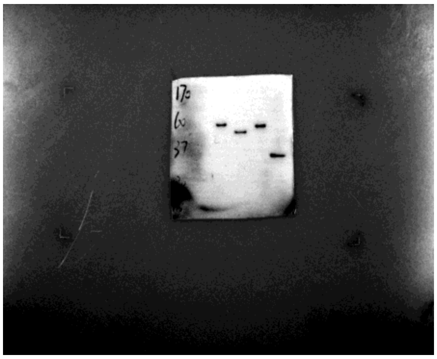

α-Tubulin

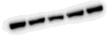

c-Cbl (IP)

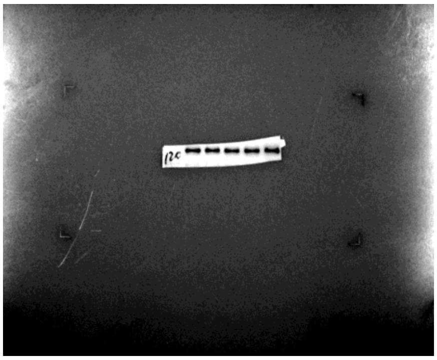

Flag (IP)

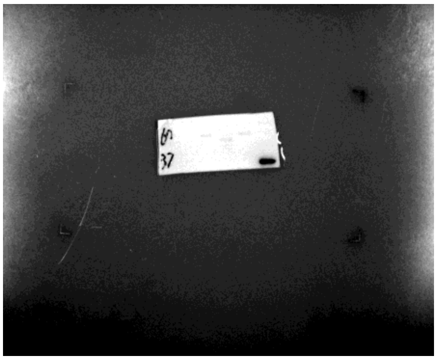

Supplementary Figure 5E

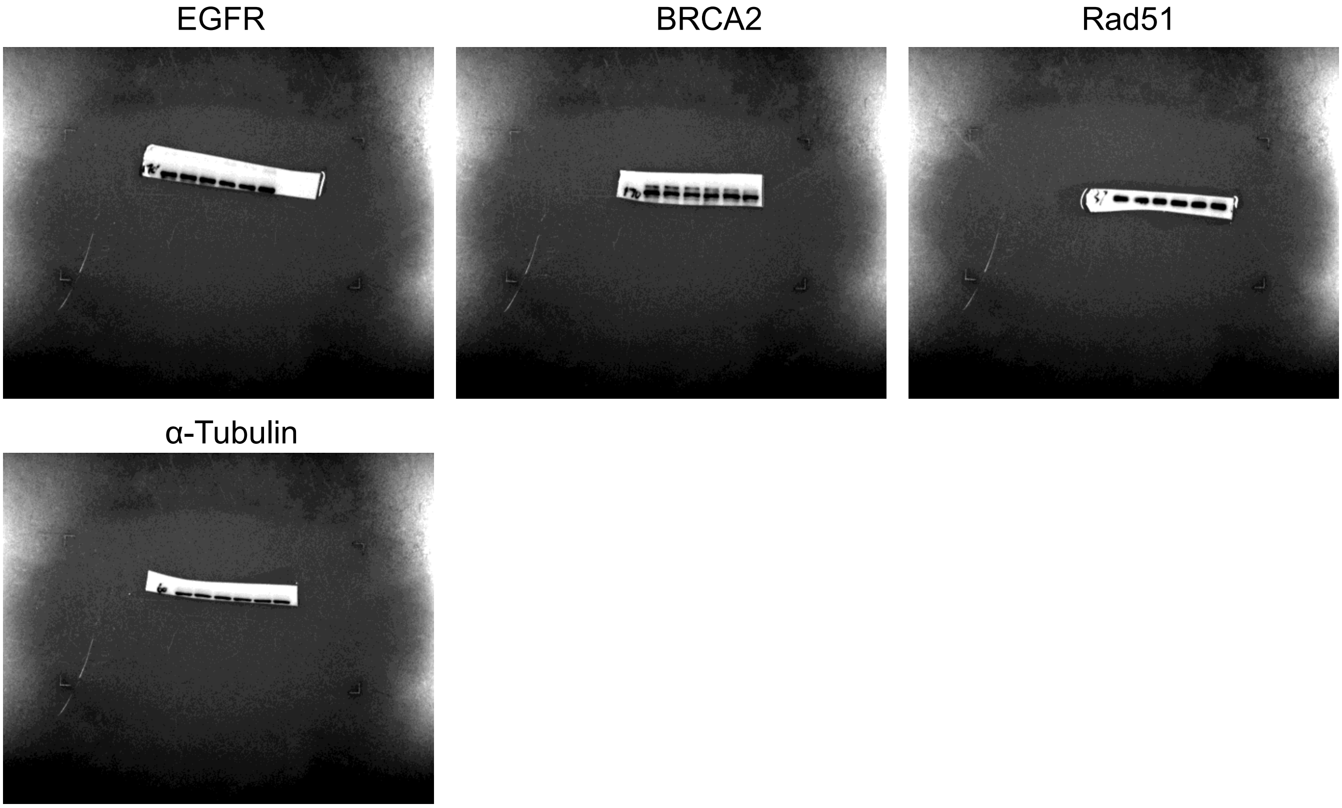

Supplementary Figure 5F

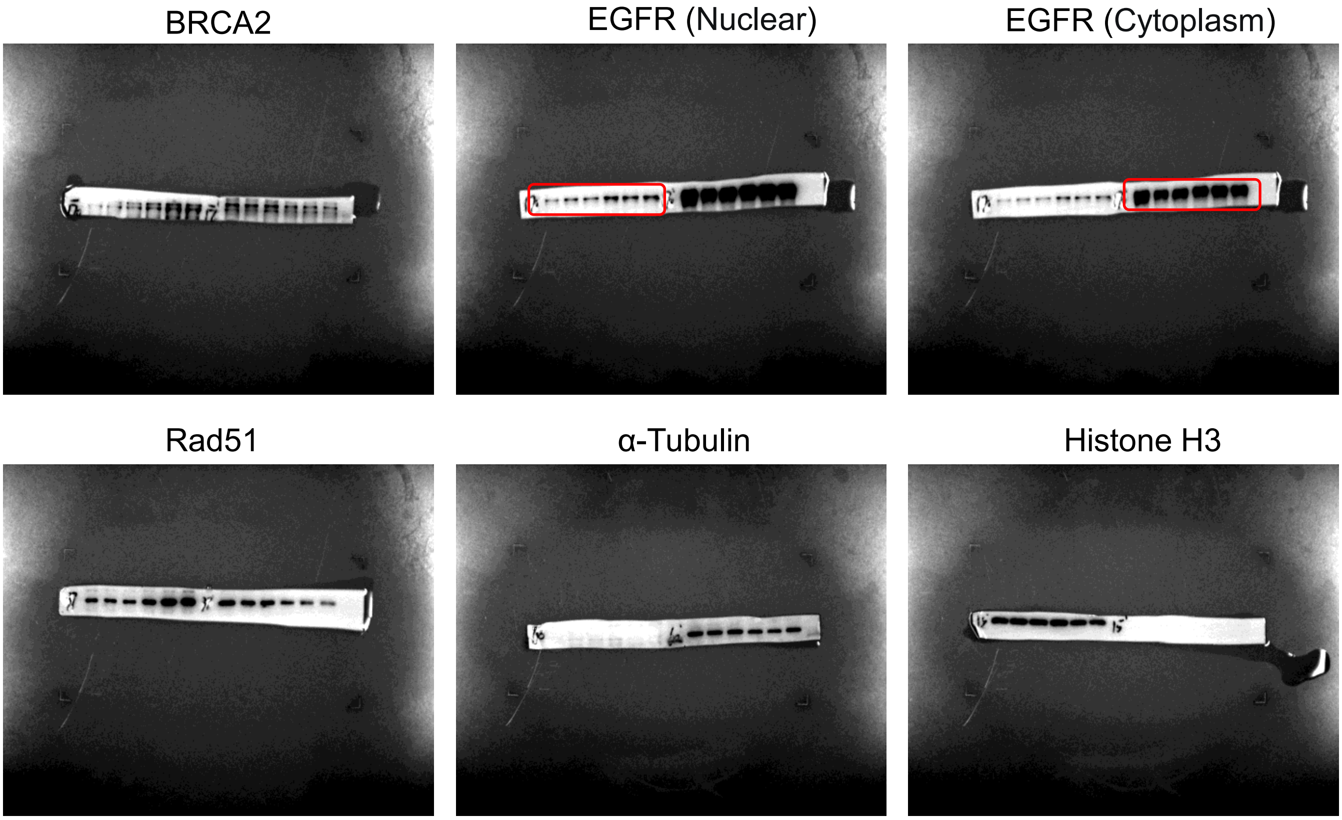

Supplementary Figure 5G (SKOV3)

BRCA2 (Nuclear)

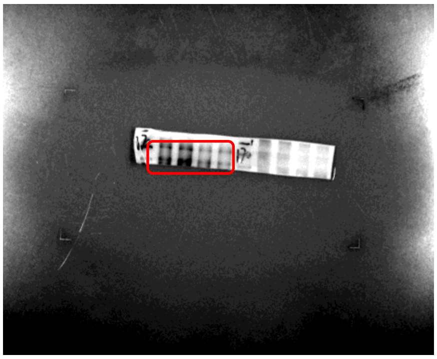

BRCA2 (Cytoplasm)

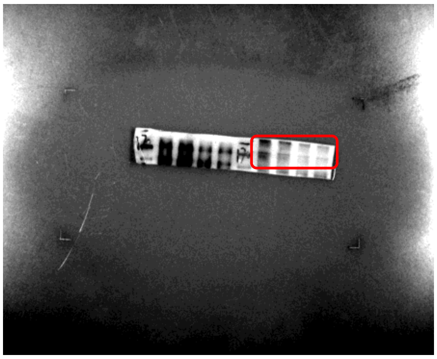

EGFR (Nuclear)

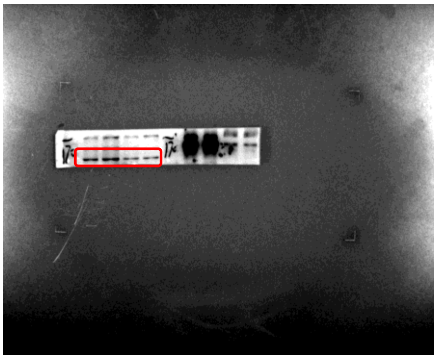

EGFR (Cytoplasm)

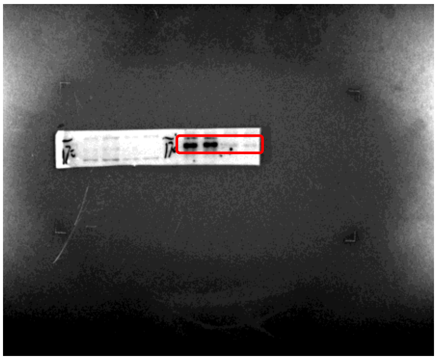

Rad51

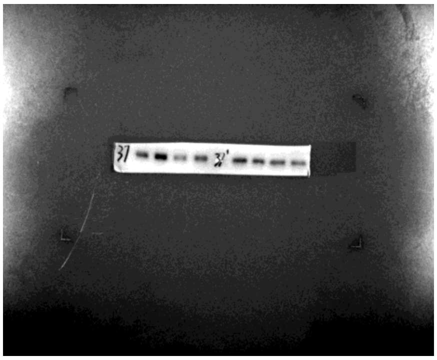

α-Tubulin

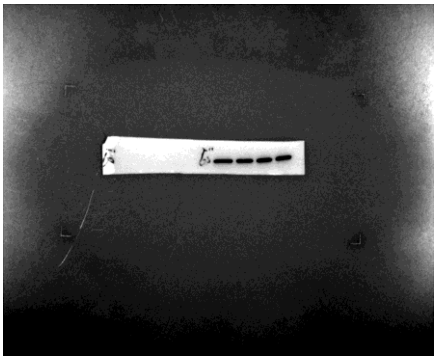

Histone H3

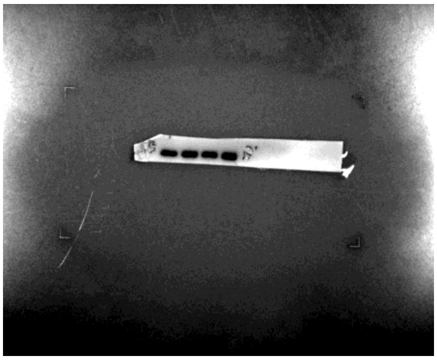

Supplementary Figure 5G (OVCAR3)

BRCA2 (Nuclear)

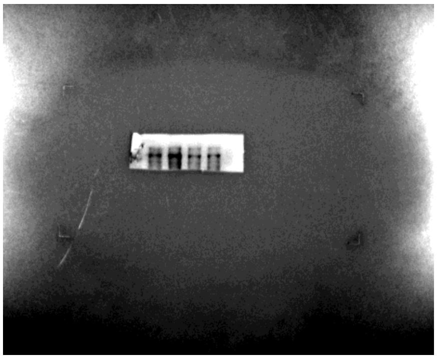

BRCA2 (Cytoplasm)

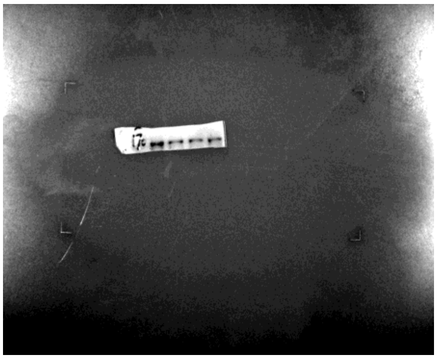

EGFR (Nuclear)

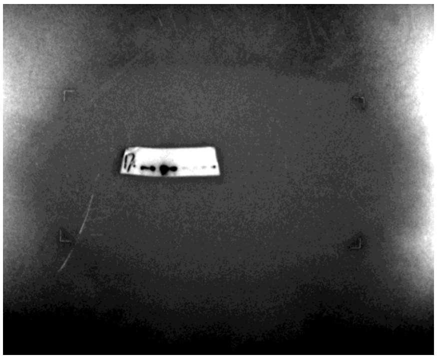

EGFR (Cytoplasm)

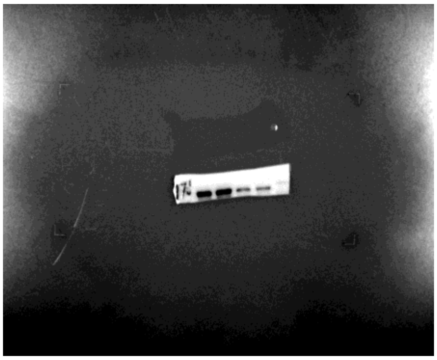

Rad51 (Nuclear)

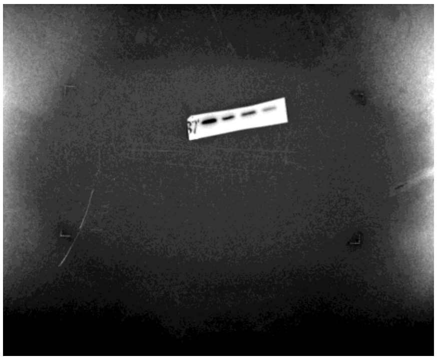

Rad51 (Cytoplasm)

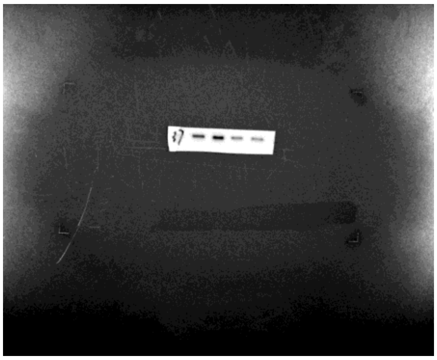

α-Tubulin

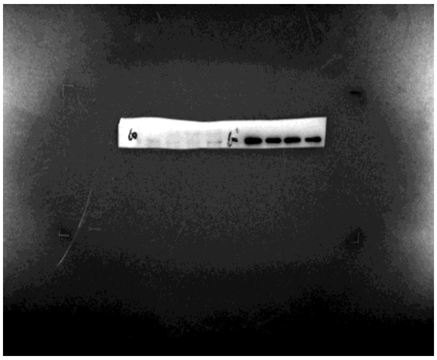

Histone H3

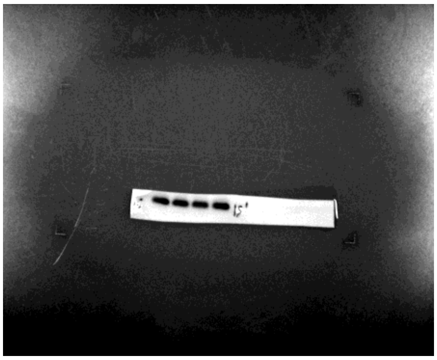

Supplementary Figure 5H (4C+Nira)

BRCA2

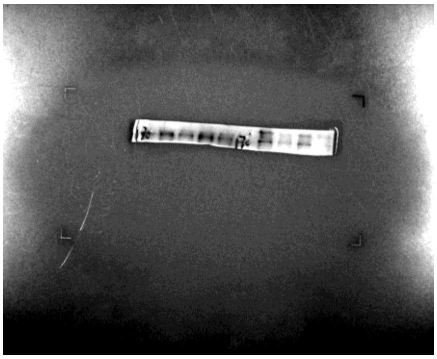

EGFR (Nuclear)

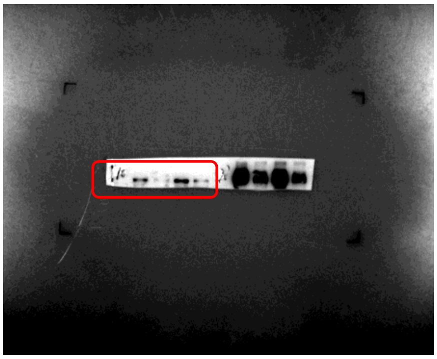

EGFR (Cytoplasm)

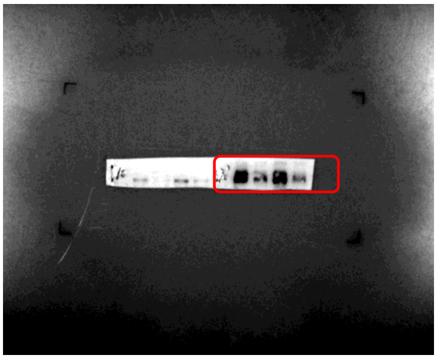

Rad51

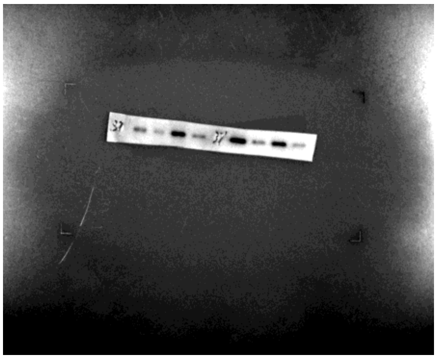

α-Tubulin

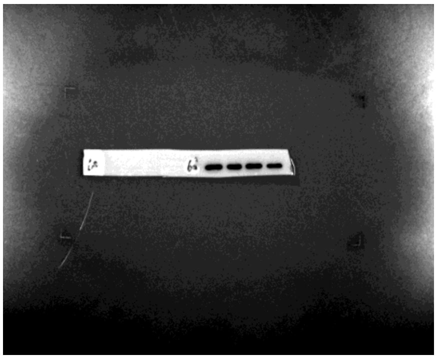

Histone H3

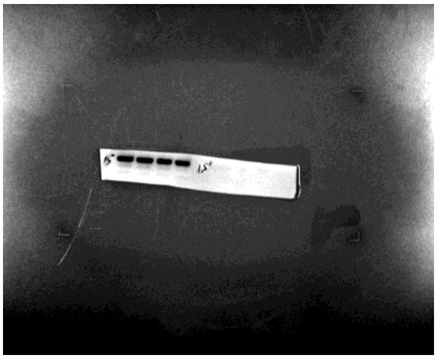

Supplementary Figure 5H (4C+Tala)

BRCA2

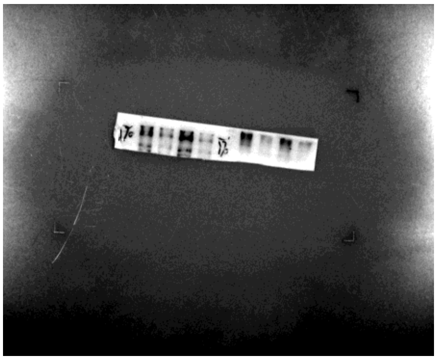

EGFR (Nuclear)

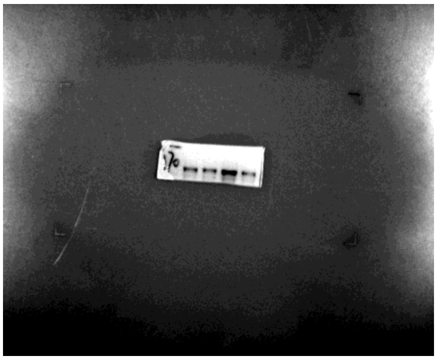

EGFR (Cytoplasm)

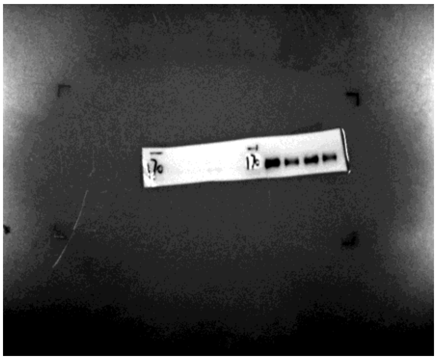

Rad51

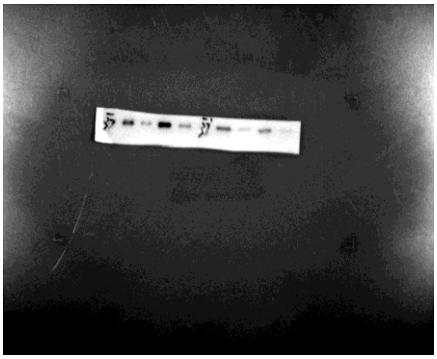

α-Tubulin

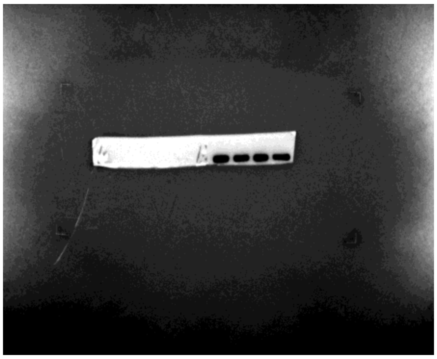

Histone H3

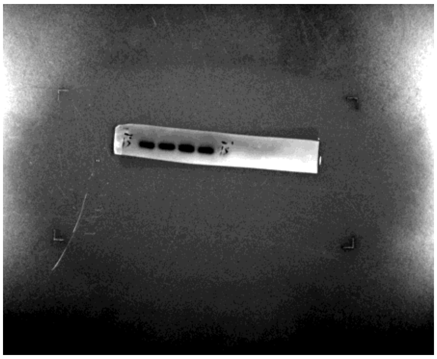

Supplementary Figure 5K (4C+Nira)

γH2AX

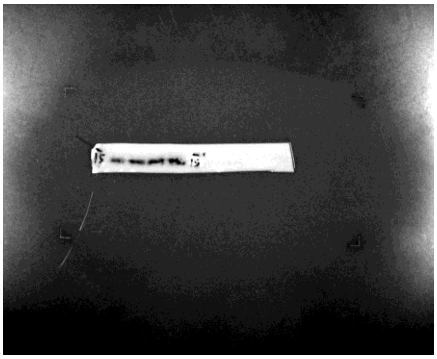

Histone H3

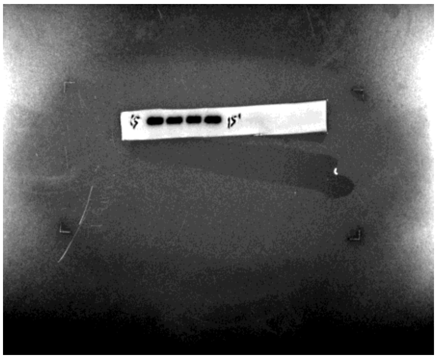

Supplementary Figure 5K (4C+Tala)

γH2AX

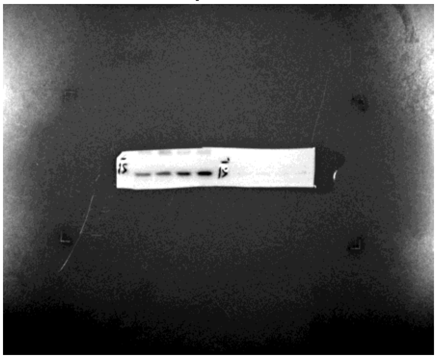

Histone H3

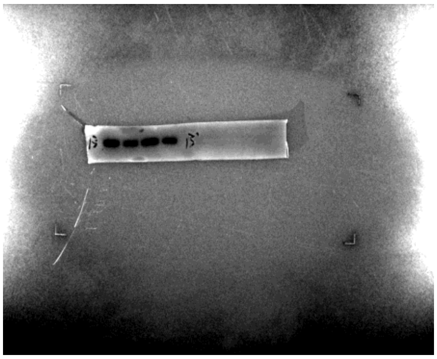

Supplementary Figure 6E

BRCA2

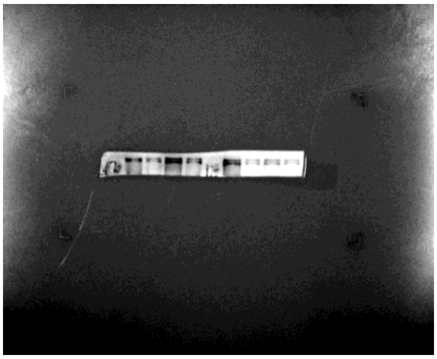

EGFR

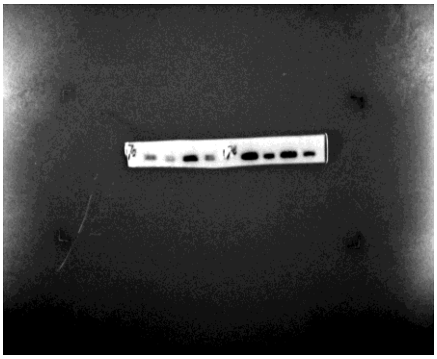

Rad51

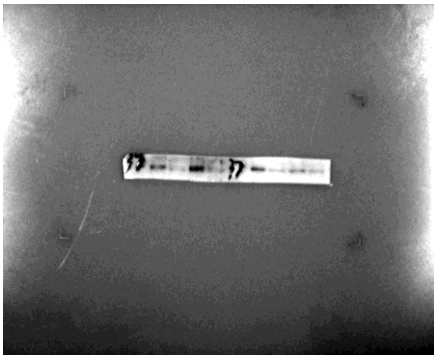

γH2AX

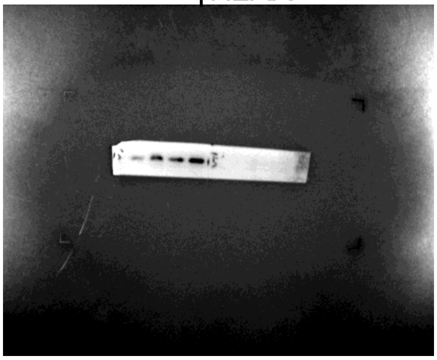

α-Tubulin

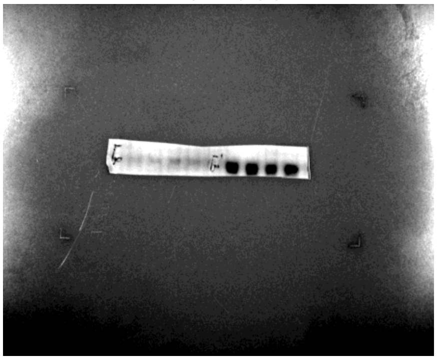

Histone H3

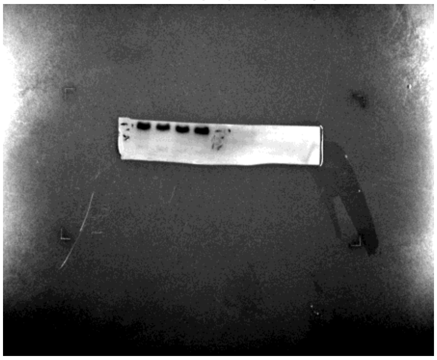

Supplement: Supplementary file 2 — Original Data File [file 41419_2026_8556_MOESM2_ESM.pdf]
